# Decomposition of the total effect for two mediators: A natural mediated interaction effect framework

**DOI:** 10.1515/jci-2020-0017

**Published:** 2022-03-19

**Authors:** Xin Gao, Li Li, Li Luo

**Affiliations:** Department of Mathematics and Statistics, University of New Mexico, Albuquerque, NM, 87131, USA; Comprehensive Cancer Center, University of New Mexico, Albuquerque, NM, 87131, USA; Department of Mathematics and Statistics, University of New Mexico, Albuquerque, NM, 87131, USA; Comprehensive Cancer Center, University of New Mexico, Albuquerque, NM, 87131, USA; Department of Internal Medicine, University of New Mexico, Albuquerque, NM, 87131, USA

**Keywords:** causal inference, interaction, mediation, causally sequential mediators, 62P10

## Abstract

Mediation analysis has been used in many disciplines to explain the mechanism or process that underlies an observed relationship between an exposure variable and an outcome variable via the inclusion of mediators. Decompositions of the total effect (TE) of an exposure variable into effects characterizing mediation pathways and interactions have gained an increasing amount of interest in the last decade. In this work, we develop decompositions for scenarios where two mediators are causally sequential or non-sequential. Current developments in this area have primarily focused on either decompositions without interaction components or with interactions but assuming no causally sequential order between the mediators. We propose a new concept called natural mediated interaction (MI) effect that captures the two-way and three-way interactions for both scenarios and extends the two-way MIs in the literature. We develop a unified approach for decomposing the TE into the effects that are due to mediation only, interaction only, both mediation and interaction, neither mediation nor interaction within the counterfactual framework. Finally, we compare our proposed decomposition to an existing method in a non-sequential two-mediator scenario using simulated data, and illustrate the proposed decomposition for a sequential two-mediator scenario using a real data analysis.

## 1 Introduction

Mediation analysis has become the technique of choice to identify and explain the mechanism that underlies an observed relationship between an exposure or treatment variable and an outcome variable via the inclusion of intermediate variables, known as mediators. Decompositions of the total effect (TE) of the exposure into effects characterizing mediation pathways and interactions help researchers understand the effects through different mechanisms and have gained much attention in the literature and application in the last decade [1–10]. In our motivating example, we are interested in the effects of drinking alcohol on systolic blood pressure (SBP) via the mediators, body mass index (BMI), and gamma-glutamyl transferase (GGT), and their interaction effects. Besides, the mediator BMI is previously reported to affect GGT and not vice versa, and hence the two mediators are causally sequential [3]. Current developments in this area for scenarios considering two mediators have primarily focused on either decomposition without interaction components, or decomposition allowing interactions but assuming no causally sequential order between the mediators [3,4,9]. Daniel et al. [3] and Steen et al. [4] discussed the decompositions in a general framework with causally sequential mediators; however, their decompositions do not include interaction components. Bellavia and Valeri [9] proposed a decomposition with components describing interactions, but they assumed these mediators are causally non-sequential. Taguri et al. [10] also considered scenarios with multiple mediators that are causally non-ordered, in which they developed a novel component termed “mediated interaction” (MI).

In this work, we develop decomposition methods for the scenarios when the two mediators are causally sequential and the interaction effects among the mediators and exposure possibly exist. Our approach also applies to a non-sequential two-mediator scenario. We present a unified approach for decomposing the TE into the components that are due to mediation only, interaction only, both mediation and interaction, neither mediation nor interaction within the counterfactual framework. Our decomposition methods are motivated by vanderWeele’s four-way decomposition [7] of the TE with one mediator, where the interaction effects include a reference interaction effect for interaction only and an MI effect for both mediation and interaction. VanderWeele [7] emphasized that these additive interaction terms are often considered of the greatest public health importance [11,12]. We also propose a new concept called natural MI effect for describing the two-way and three-way interactions in two-mediator scenarios that extend the MI from VanderWeele’s work [7]. Since the causal structures are more complex with two mediators, the decompositions have multiple terms for mediation only, interaction only, and both mediation and interaction. Identifiability issues appear in the presence of time-varying confounders, which will be naturally introduced by the mediators in a sequential structure [13,14]. We lay out the identification assumptions and provide identifiable counterfactual formulas in our proposed decomposition [15].

When the two mediators are casually non-sequential, our decomposition uses a different approach from what was proposed by Bellavia and Valeri [9]. For example, their population-averaged MI effect between *A* and *M*_1_ is evaluated with *M*_2_ fixed at a certain level while our natural MI effect between *A* and *M*_1_ provides a natural interpretation and is essentially a weighted MI effect where the weights are determined by the distribution of *M*_2_ in the population.

The rest of the article is organized as follows: Section 2 reviews VanderWeele’s four-way decomposition; Section 3 presents decompositions of TE for two-mediator scenarios; Section 4 relates the components of our proposed decompositions to the traditional definitions; Section 5 lays out identification assumptions and gives the empirical and regression-based formulas for computing each component in the decomposition with two causally sequential mediators; Section 6 presents a simulation study and real data analysis; and Section 7 concludes the article with discussions.

## 2 Decomposition of the TE in a single-mediator scenario

### 2.1 Counterfactual definitions

**Figure 1:**
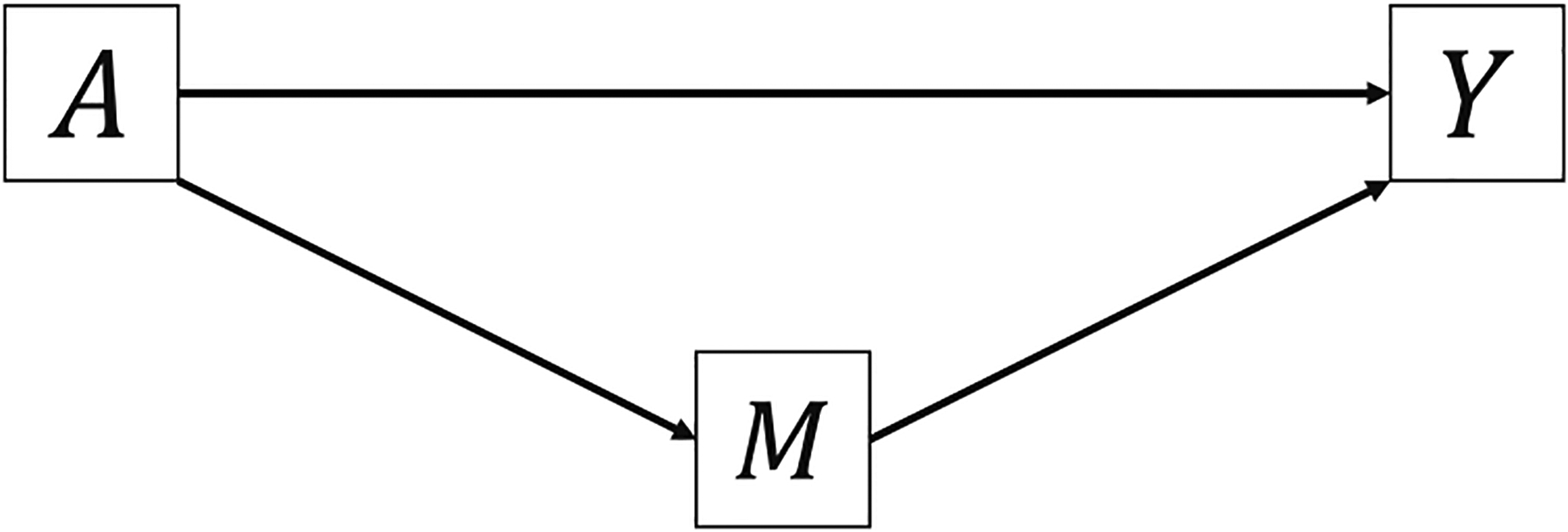
Directed acyclic graph of a single-mediator scenario.

**Figure 2:**
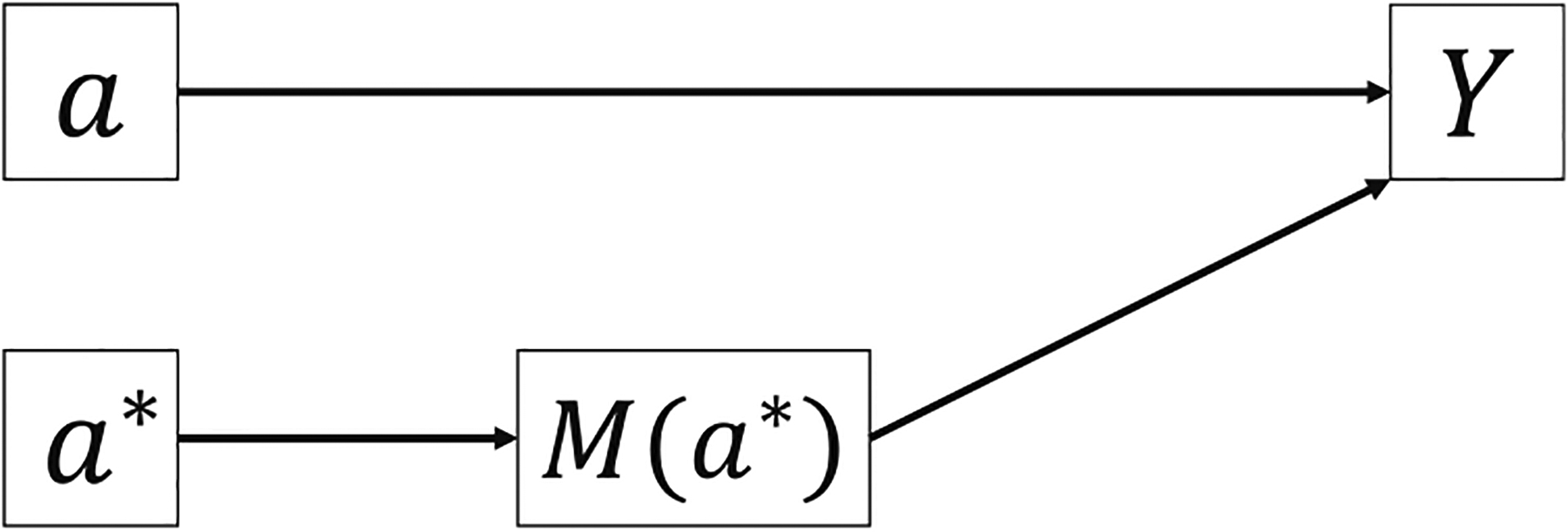
Graphical illustration of the nested counterfactual formula *Y*(*a*, *M*_1_(a*).

Consider a single-mediator scenario in Figure 1. Counterfactual formulas give the potential value of outcome *Y* or mediator *M* that would have been observed if the exposure *A* or mediator *M* were fixed at a certain level [8,16,17]. Let *Y*(*a*) denote the potential value of *Y* that would have been observed if the exposure *A* were fixed at a constant level *a* [8]. Similarly, *M*(*a*) denotes the potential value of *M* that would have been observed if *A* were fixed at *a* and *Y*(*a*, *m*) denotes the potential value of *Y* that would have been observed if *A* and *M* were fixed at *a* and *m*, respectively [8]. A nested counterfactual formula *Y*(*a*, *M*(a*)) denotes the potential value of *Y* that would have been observed if the exposure were fixed at *a* and the mediator *M* were set to what would have been observed or potential value when the exposure were fixed at a* (Figure 2) [8].

### 2.2 Two-way decomposition

The TE of the exposure *A* for an individual is defined as the difference between *Y*(*a*) and *Y*(a*) [8], where *a* and a* are the treatment and reference level of the exposure *A*, respectively. The classical decomposition of the TE has two components: natural direct effect (NDE) and natural indirect effect (NIE) [8,17,18]. NDE represents the causal effect along the direct path from *A* to *Y* and NIE represents the causal effect along the indirect path from *A* through *M* to *Y*. The effects are defined using the following formulas:

$$\text{TE}=Y(a)-Y\left(a^{*}\right)\;=Y(a,M(a))-Y\left(a^{*},M\left(a^{*}\right)\right)\;=Y(a,M(a))-Y\left(a,M\left(a^{*}\right)\right)+Y\left(a,M\left(a^{*}\right)\right)-Y\left(a^{*},M\left(a^{*}\right)\right)\text{,}$$

$$\text{NDE}=Y\left(a,M\left(a^{*}\right)\right)-Y\left(a^{*},M\left(a^{*}\right)\right),$$

$$\text{NIE}=Y(a,M(a))-Y\left(a,M\left(a^{*}\right)\right).$$

The second equality of TE follows by the composition axiom [8,15] and the third equality of TE follows by subtracting and adding the same counterfactual formula *Y*(*a*, *M*(a*)). NDE is the difference in the potential value of outcome when *A* goes from a* to *a* and *M* is at its potential value *M*(a*). NIE is the difference in the potential value of outcome had *M* gone from *M*(a*) to *M*(*a*) while *A* is at its treatment level *a*. In the literature, NDE and NIE are also referred to as pure direct effect (PDE) and total indirect effect (TIE) [16], respectively. Furthermore, NDE also corresponds to a path-specific effect proposed by Pearl [17].

### 2.3 Four-way decomposition with interactions

VanderWeele [7] proposed a four-way decomposition in a single-mediator scenario where the exposure interacts with the mediator. The TE of the exposure on the outcome is decomposed into components due to mediation only, interaction only, both mediation and interaction, and neither mediation nor interaction. These four components are termed as pure indirect effect (PIE), reference interaction effect (INT_ref_(m*)), MI effect (INT_med_), and controlled direct effect (CDE(m*)), respectively, where m* is an arbitrarily chosen fixed reference level of the mediator *M*. At the individual level, the four components are expressed in the following general forms [7]:

$$\text{CDE}\left(m^{*}\right)=Y\left(a,m^{*}\right)-Y\left(a^{*},m^{*}\right),$$

$${\text{INT}}_{\text{ref}}\left(m^{*}\right)=\underset{m}{\sum }\left[Y(a,m)-Y\left(a^{*},m\right)-Y\left(a,m^{*}\right)+Y\left(a^{*},m^{*}\right)\right]\times I\left(M\left(a^{*}\right)=m\right),$$

$${\text{INT}}_{\text{med}}=\underset{m}{\sum }\left[Y(a,m)-Y\left(a^{*},m\right)-Y\left(a,m^{*}\right)+Y\left(a^{*},m^{*}\right)\right]\times \left[I(M(a)=m)-I\left(M\left(a^{*}\right)=m\right)\right],$$

$$\text{PIE}=\underset{m}{\sum }\left[Y\left(a^{*},m\right)-Y\left(a^{*},m^{*}\right)\right]\times \left[I(M(a)=m)-I\left(M\left(a^{*}\right)=m\right)\right].$$

The reference and MI effects can also be expressed in the form of the counterfactual formulas in our view:

$${\text{INT}}_{\text{ref}}\left(m^{*}\right)=Y\left(a,M\left(a^{*}\right)\right)-Y\left(a^{*},M\left(a^{*}\right)\right)-Y\left(a,m^{*}\right)+Y\left(a^{*},m^{*}\right),$$

$${\text{INT}}_{\text{med}}=Y(a,M(a))-Y\left(a^{*},M(a)\right)-Y\left(a,M\left(a^{*}\right)\right)+Y\left(a^{*},M\left(a^{*}\right)\right).$$

CDE measures the effect of *A* had *M* been fixed at level m*. INT_ref_(m*) measures the change in the effect of *A* had *M* gone from m* to *M*(a*). If *M*(a*) = m*, INT_ref_(m*) for the individual considered is reduced to zero. INT_med_ describes the change in the effect of *A* had *M* gone from *M*(a*) to *M*(*a*). When *A* has no effect on the mediator, *M*(a*) = *M*(*a*), and INT_med_ becomes zero. *PIE* describes the effect of *M* when *A* is set at a* and *M* goes from *M*(a*) to *M*(*a*).

When *A* and *M* are both binary with the conditions *a* = 1, a* = 0, and m* = 0, the counterfactual definitions of the components become:

CDE(0)=Y(1,0)−Y(0,0),

$${\text{INT}}_{\text{ref}}(0)=[Y(1,1)-Y(1,0)-Y(0,1)+Y(0,0)]\times M(0),$$

$${\text{INT}}_{\text{med}}=[Y(1,1)-Y(1,0)-Y(0,1)+Y(0,0)]\times [M(1)-M(0)],$$

PIE=[Y(0,1)−Y(0,0)]×[M(1)−M(0)],

where 1 is the treatment level and 0 is the reference level [7].

Both INT_ref_ and INT_med_ have an additive interaction [*Y*(1, 1) − *Y*(1, 0) − *Y*(0, 1) + *Y*(0, 0)] term, which will be non-zero for an individual if the joint effect of having both the exposure and the mediator present differs from the sum of the effects of having only the exposure or mediator present. The additive interaction effect is generally considered of great public health importance [11,12]. Provided the additive interaction exists, the difference between INT_ref_ and INT_med_ is that INT_ref_ is non-zero only if the mediator is present in the absence of exposure (i.e., *M*(0) = 1), whereas INT_med_ is non-zero only if the exposure has an effect on the mediator (i.e., *M*(1) − *M*(0) ≠ 0).

Based on the counterfactual formula form of MI INT_med_, we propose the natural MI effect and provide the following definition. The MI effect and natural MI effect are mathematically equivalent in a single-mediator scenario; we define it from a different perspective only for building up the concepts for scenarios with two mediators in Section 3.

**Definition 1**. We define the natural MI effect of *A* and *M* (NatINT_AM_) to be the MI effect (INT_med_) in a single-mediator scenario:

$${\text{NatINT}}_{\text{AM}}≔{\text{INT}}_{\text{med}}=Y(a,M(a))-Y\left(a^{*},M(a)\right)-Y\left(a,M\left(a^{*}\right)\right)+Y\left(a^{*},M\left(a^{*}\right)\right)\text{,}$$

where *M*(a*) and *M*(*a*) denote the potential values of *M* that would have occurred if *A* were fixed at a* and *a*, respectively.

## 3 Decomposition of the TE in two-mediator scenarios

When two mediators are considered, two-way interaction of the two mediators and three-way interaction of the exposure and the two mediators are likely to exist [7–9]. There may also be a causal sequence between the two mediators, i.e., there is a direct causal link between the two mediators. There is limited research on how to define interactions when the two mediators are causally sequential. We aim to develop interpretable interaction concepts and decomposition approaches for two-mediator scenarios.

### 3.1 Mediators causally non-sequential

**Figure 3:**
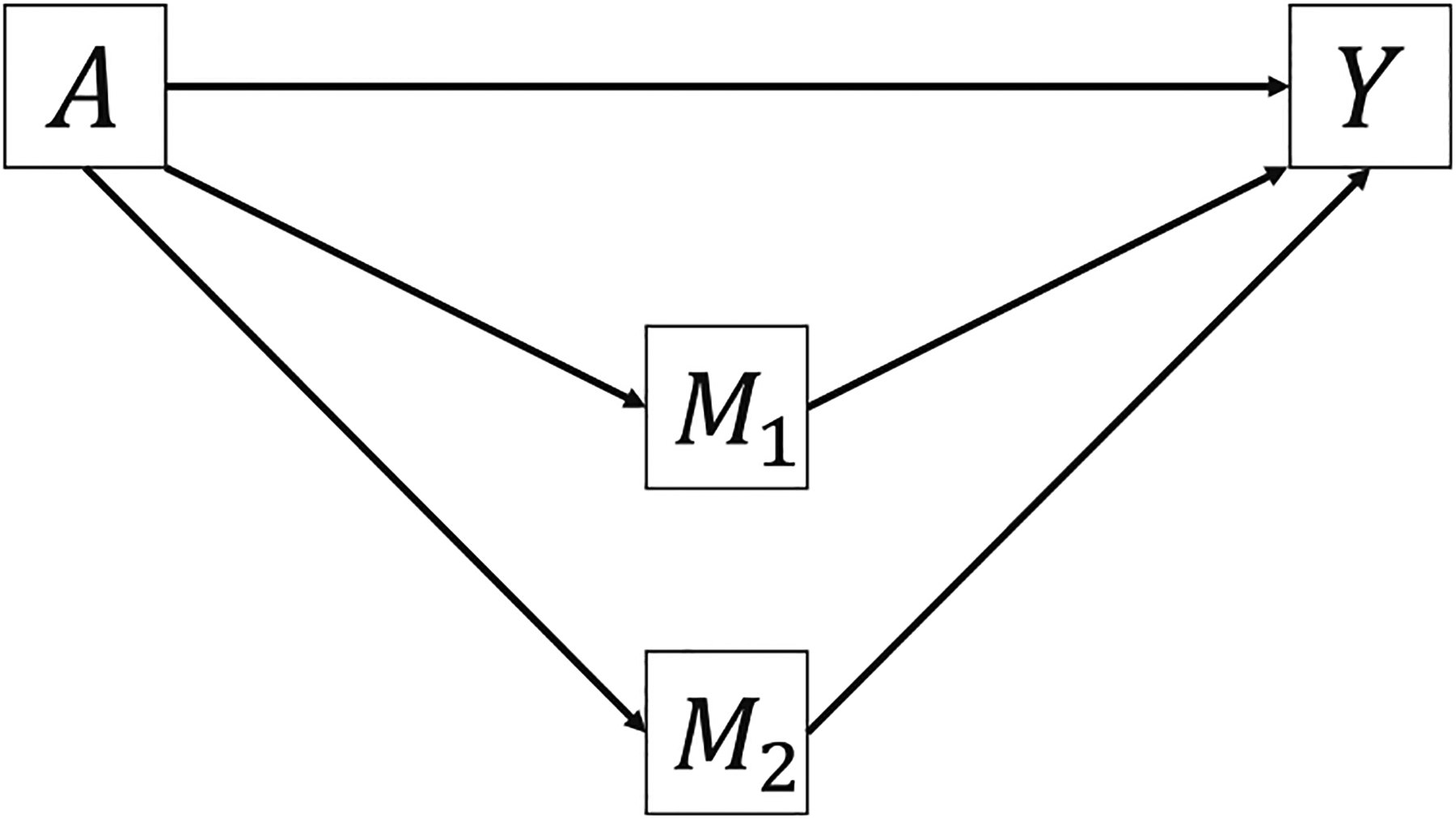
Directed acyclic graph with two non-sequential mediators.

We first consider the scenario when the two mediators are causally non-sequential, i.e., there is no direct causal link between the two mediators, which is shown in Figure 3. Below, we define two-way natural MI effects of *A* and *M*_1_, *A* and *M*_2_, *M*_1_ and *M*_2_, and a three-way natural MI effect of *A*, *M*_1_, and *M*_2_.

**Definition 2**. Natural MI effects in a causally non-sequential two-mediator scenario are defined as follows:

$${\text{NatINT}}_{AM_{1}}≔Y\left(a,M_{1}(a),M_{2}\left(a^{*}\right)\right)-Y\left(a^{*},M_{1}(a),M_{2}\left(a^{*}\right)\right)-Y\left(a,M_{1}\left(a^{*}\right),M_{2}\left(a^{*}\right)\right)+Y\left(a^{*},M_{1}\left(a^{*}\right),M_{2}\left(a^{*}\right)\right),$$

$${\text{NatINT}}_{AM_{2}}≔Y\left(a,M_{1}\left(a^{*}\right),M_{2}(a)\right)-Y\left(a^{*},M_{1}\left(a^{*}\right),M_{2}(a)\right)-Y\left(a,M_{1}\left(a^{*}\right),M_{2}\left(a^{*}\right)\right)+Y\left(a^{*},M_{1}\left(a^{*}\right),M_{2}\left(a^{*}\right)\right),$$

$${\text{NatINT}}_{M_{1}M_{2}}≔Y\left(a^{*},M_{1}(a),M_{2}(a)\right)-Y\left(a^{*},M_{1}\left(a^{*}\right),M_{2}(a)\right)-Y\left(a^{*},M_{1}(a),M_{2}\left(a^{*}\right)\right)+Y\left(a^{*},M_{1}\left(a^{*}\right),M_{2}\left(a^{*}\right)\right),$$

$${\text{NatINT}}_{AM_{1}M_{2}}≔Y\left(a,M_{1}(a),M_{2}(a)\right)-Y\left(a^{*},M_{1}(a),M_{2}(a)\right)-Y\left(a,M_{1}\left(a^{*}\right),M_{2}(a)\right)+Y\left(a^{*},M_{1}\left(a^{*}\right),M_{2}(a)\right)-Y\left(a,M_{1}(a),M_{2}\left(a^{*}\right)\right)+Y\left(a^{*},M_{1}(a),M_{2}\left(a^{*}\right)\right)+Y\left(a,M_{1}\left(a^{*}\right),M_{2}\left(a^{*}\right)\right)-Y\left(a^{*},M_{1}\left(a^{*}\right),M_{2}\left(a^{*}\right)\right).$$

${\text{NatINT}}_{AM_{1}}$, ${\text{NatINT}}_{AM_{2}}$, and ${\text{NatINT}}_{AM_{1}M_{2}}$ are components that capture the effects due to both mediation and interaction with the exposure. ${\text{NatINT}}_{M_{1}M_{2}}$ describes the effect due to mediation and interaction between the two mediators. When measuring the interaction between *A* and *M*_1_, *M*_2_ is not fixed but takes its potential value *M*_2_(a*) for each individual had the exposure been the reference level. Similarly, when measuring the interaction between *A* and *M*_2_, *M*_1_ is not fixed but takes its potential value *M*_1_(a*) for the individual. The three-way interaction ${\text{NatINT}}_{AM_{1}M_{2}}$ is similar to a three-way additive interaction. To demonstrate the similarity, we consider that *A* is binary with the conditions *a* = 1 and a* = 0; ${\text{NatINT}}_{AM_{1}M_{2}}$ becomes

$$Y\left(1,M_{1}(1),M_{2}(1)\right)-Y\left(0,M_{1}(1),M_{2}(1)\right)-Y\left(1,M_{1}(0),M_{2}(1)\right)+Y\left(0,M_{1}(0),M_{2}(1)\right)-Y\left(1,M_{1}(1),M_{2}(0)\right)+Y\left(0,M_{1}(1),M_{2}(0)\right)+Y\left(1,M_{1}(0),M_{2}(0)\right)-Y\left(0,M_{1}(0),M_{2}(0)\right).$$

The above three-way interaction measures the change in the two-way interaction between *A* and *M*_1_ when *M*_2_ goes from *M*_2_(0) to *M*_2_(1). It also measures the change in the interaction between *A* and *M*_2_ when *M*_1_ goes from *M*_1_(0) to *M*_1_(1) or the change in the interaction between *M*_1_ and *M*_2_ when *A* goes from 0 to 1.

In Supplementary material S1, we show that the TE can be decomposed into ten components at the individual level:

$$\text{TE}=\text{CDE}\left(m_{1}^{*},m_{2}^{*}\right)+{\text{INT}}_{\text{ref-}AM_{1}}\left(m_{1}^{*},m_{2}^{*}\right)+{\text{INT}}_{\text{ref-}AM_{2}}\left(m_{1}^{*},m_{2}^{*}\right)+{\text{INT}}_{\text{ref-}AM_{1}M_{2}}\left(m_{1}^{*},m_{2}^{*}\right)+{\text{NatINT}}_{AM_{1}}+{\text{NatINT}}_{AM_{2}}+{\text{NatINT}}_{AM_{1}M_{2}}+{\text{NatINT}}_{M_{1}M_{2}}+{\text{PIE}}_{M_{1}}+{\text{PIE}}_{M_{2}},$$

where $m_{1}^{*}$ and $m_{2}^{*}$ are fixed reference levels for *M*_1_ and *M*_2_, respectively, and

$$\text{CDE}\left(m_{1}^{*},m_{2}^{*}\right)=Y\left(a,m_{1}^{*},m_{2}^{*}\right)-Y\left(a^{*},m_{1}^{*},m_{2}^{*}\right),$$

$${\text{INT}}_{\text{ref-}AM_{1}}\left(m_{1}^{*},m_{2}^{*}\right)=Y\left(a,M_{1}\left(a^{*}\right),m_{2}^{*}\right)-Y\left(a^{*},M_{1}\left(a^{*}\right),m_{2}^{*}\right)-Y\left(a,m_{1}^{*},m_{2}^{*}\right)+Y\left(a^{*},m_{1}^{*},m_{2}^{*}\right),$$

$${\text{INT}}_{\text{ref-}AM_{2}}\left(m_{1}^{*},m_{2}^{*}\right)=Y\left(a,m_{1}^{*},M_{2}\left(a^{*}\right)\right)-Y\left(a^{*},m_{1}^{*},M_{2}\left(a^{*}\right)\right)-Y\left(a,m_{1}^{*},m_{2}^{*}\right)+Y\left(a^{*},m_{1}^{*},m_{2}^{*}\right),$$

$${\text{INT}}_{{\text{ref-}AM}_{1}M_{2}}\left(m_{1}^{*},m_{2}^{*}\right)=Y\left(a,M_{1}\left(a^{*}\right),M_{2}\left(a^{*}\right)\right)-Y\left(a^{*},M_{1}\left(a^{*}\right),M_{2}\left(a^{*}\right)\right)-Y\left(a,m_{1}^{*},M_{2}\left(a^{*}\right)\right)+Y\left(a^{*},m_{1}^{*},M_{2}\left(a^{*}\right)\right)-Y\left(a,M_{1}\left(a^{*}\right),m_{2}^{*}\right)+Y\left(a^{*},M_{1}\left(a^{*}\right),m_{2}^{*}\right)+Y\left(a,m_{1}^{*},m_{2}^{*}\right)-Y\left(a^{*},m_{1}^{*},m_{2}^{*}\right),$$

$${\text{PIE}}_{M_{1}}=Y\left(a^{*},M_{1}\left(a\right),M_{2}(a^{*})\right)-Y\left(a^{*},M_{1}\left(a^{*}\right),M_{2}\left(a^{*}\right)\right),$$

$${\text{PIE}}_{M_{2}}=Y\left(a^{*},M_{1}\left(a^{*}\right),M_{2}(a)\right)-Y\left(a^{*},M_{1}\left(a^{*}\right),M_{2}\left(a^{*}\right)\right).$$

Similar to the four-way decomposition, CDE denotes controlled direct effect due to neither mediation nor interaction, INT_ref_s denote reference interaction effects due to interactions only, and PIEs denote PIEs due to mediation only [7,16,17]. ${\text{NatINT}}_{M_{1}M_{2}}$ can be interpreted as the effect due to the mediation through both *M*_1_ and *M*_2_, and the interaction between *M*_1_ and *M*_2_. Since the interaction is not involved with the change in exposure *A*, the interpretation can be simply put as the effect due to the mediation through both *M*_1_ and *M*_2_ only. These ten components are displayed in Table 1 assuming that *A*, *M*_1_, and *M*_2_ are binary with *a* = 1, a* = 0, $m_{1}^{*}=0$, and $m_{2}^{*}=0$.

**Figure 4:**
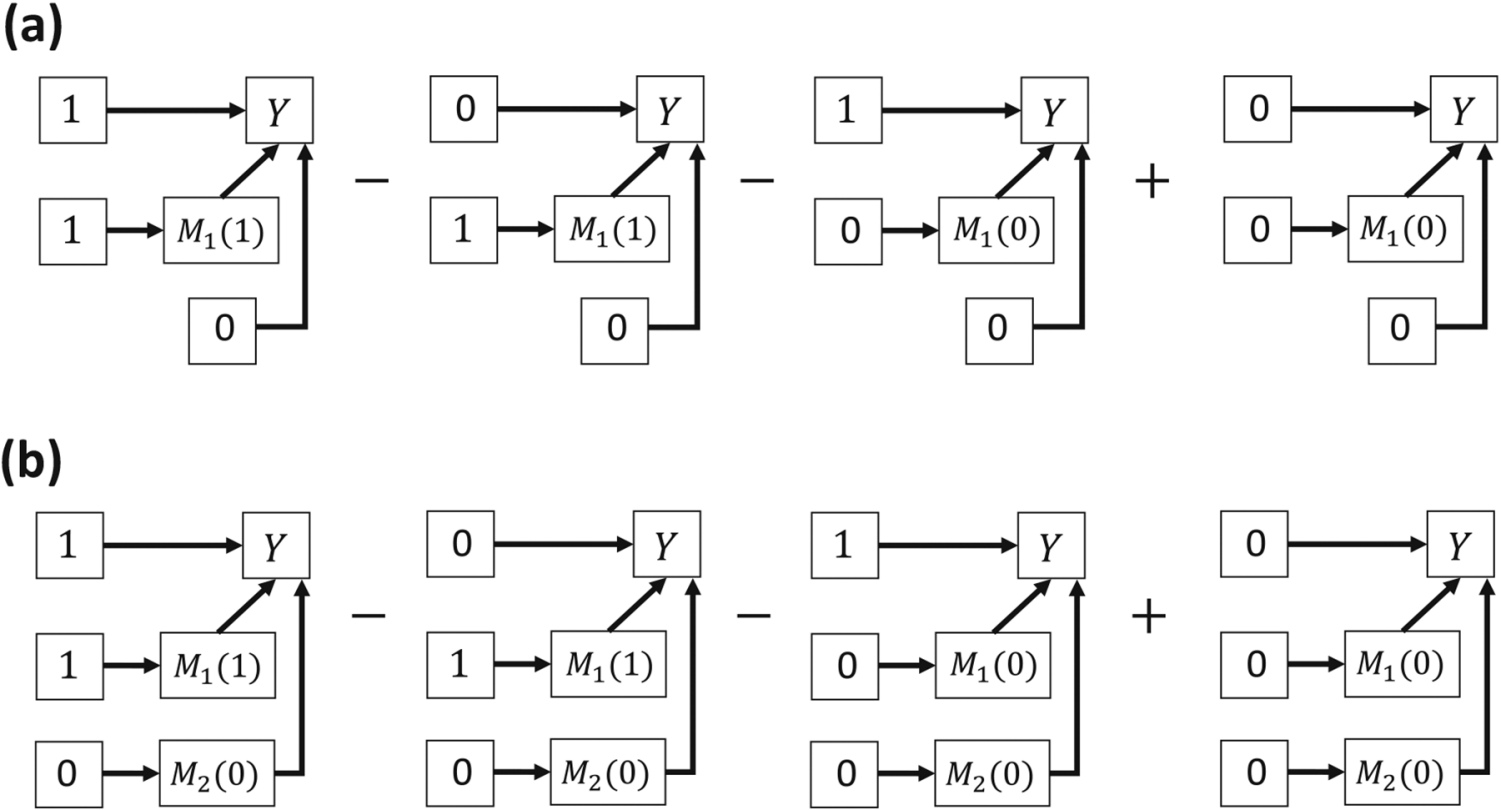
Comparison between the MI effect and the natural MI effect between *A* and *M*_1_ at the individual level in a non-sequential two-mediator scenario. (a) ${\text{INTmed}}_{AM_{1}}$ in Bellavia’s and Valeri’s method, where *M*_2_ is assumed to be fixed at 0 for all individuals. (b) ${\text{NatINT}}_{AM_{1}}$ where *M*_2_ takes its potential value *M*_2_(0) without such assumption.

Bellavia and Valeri [9] proposed a ten-way decomposition for the same directed acyclic graph in Figure 3. We show in Supplementary material S2 that their decomposition resembles our proposed decomposition under certain conditions. Their CDE and INT_ref_s are identical to the corresponding terms in our decomposition but their MI effects and pure NIEs are generally different from our natural MIs and PIEs. Figure 4a illustrates their MI effect between *A* and *M*_1_ where *M*_2_ is assigned a fixed value at $m_{2}^{*}=0$ assuming *M*_1_ and *M*_2_ are binary. Figure 4b illustrates the natural MI effect between *A* and *M*_1_, where both *M*_1_ and *M*_2_ take their potential values. In another publication, Taguri et al. [10] developed a four-way decomposition method and proposed the MI component to examine the contribution of the additive interaction effects between the mediators to the joint NIE, assuming that the mediators are not causally ordered. Our natural MI effect between *M*_1_ and *M*_2_ has some similarity to the MI component in terms of mathematical forms. However, there are three main differences between the Taguri et al. method and our proposed decomposition method. First, our ten-way decomposition also considers the MI effects between the exposure and the mediators. Second, the exposure is fixed at the treatment level in the MI component but our natural MI between *M*_1_ and *M*_2_ sets the exposure at the reference level. Third, our decomposition methods apply to scenarios with two causally sequential or non-sequential mediators.

The expected values of our natural MI effects provide natural interpretations by accounting for the distributions of *M*_1_(0) and *M*_2_(0). For example, if the population distribution of *M*_2_(0) has a probability of 1 taking the value 0, $E\left[{\text{NatINT}}_{AM_{1}}\right]$ becomes the expected value of the MI effect between *A* and *M*_1_ as proposed by Bellavia and Valeri. However, if the population distribution of *M*_2_(0) does not have a probability of 1 taking the value 0, $E\left[{\text{NatINT}}_{AM_{1}}\right]$ is more suitable to describe the population average of the counterfactual interaction effect. Table 2 presents our results of natural MI effects and PIEs under the assumption *M*_1_(0) = *M*_2_(0) = 0, which are identical to those proposed by Bellavia and Valeri [9]. A detailed comparison of the mediated effects between Bellavia’s and Valeri’s method and our proposed decomposition under linear models assuming continuous mediators and outcome is described in Section 5.3 and Table 5. The differences between the two methods are further discussed in Section 6.1 with a simulated data set.

### 3.2 Mediators causally sequential

**Figure 5:**
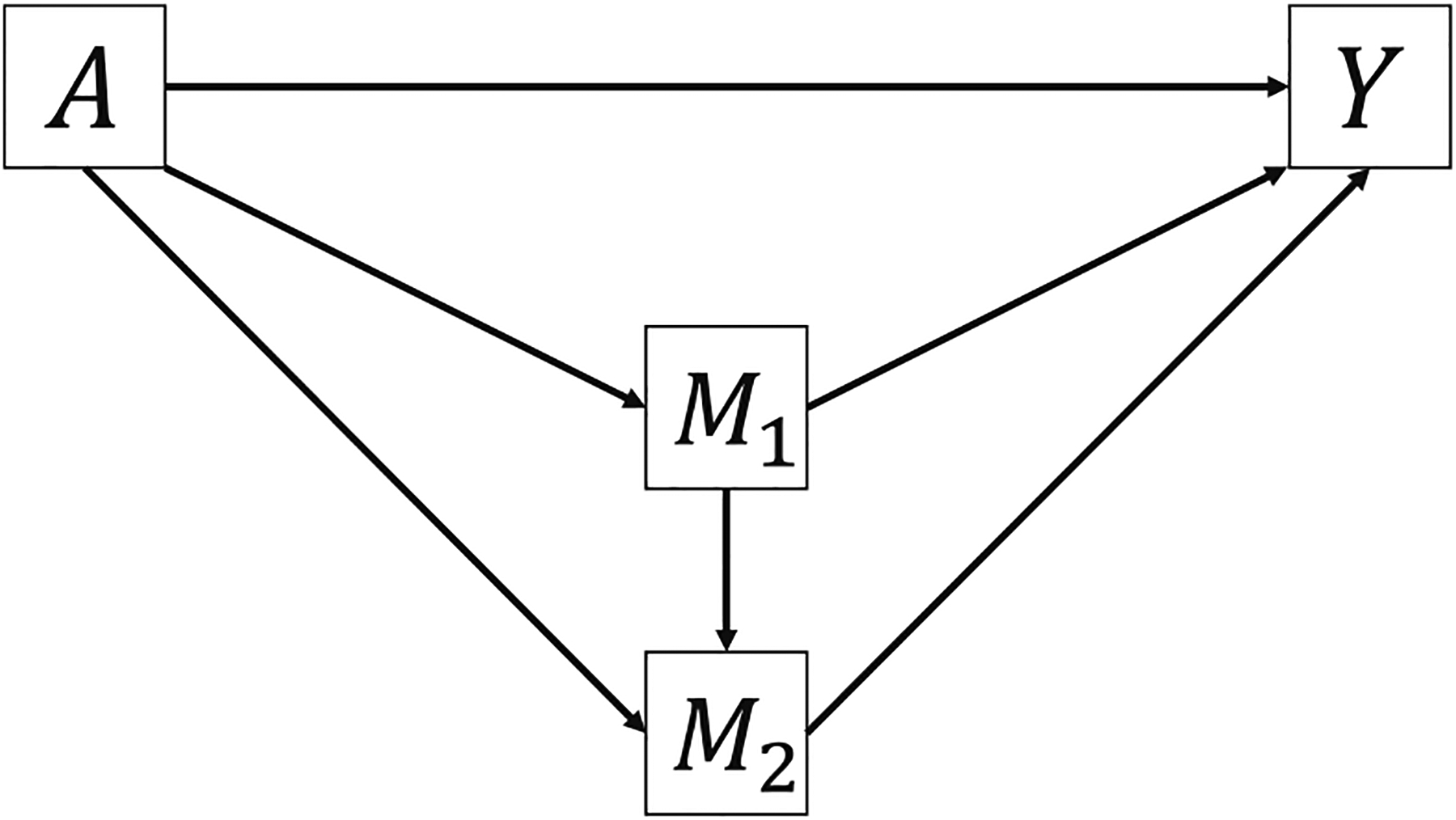
Directed acyclic graph with two sequential mediators where there exists a direct causal link pointing from *M*_1_ to *M*_2_.

In this section, we consider the scenario where the two mediators are causally sequential, i.e., there is a direct causal link from mediator *M*_1_ to *M*_2_ (Figure 5). Let *M*_2_(a*, *M*_1_(*a*)) be the potential value of *M*_2_ if *A* were fixed at a* and *M*_1_ were at its potential value had *A* been set at *a*. Similarly, *M*_2_(a*, *M*_1_(*a*)) denotes the potential value of *M*_2_ if *A* were fixed at a* and *M*_1_ were at its potential value had *A* been set at a*. Counterfactual values for *Y* are expressed using nested formulas but not all of them are non-parametrically identifiable [15]. For example, *Y*(*a*, *M*_1_(*a*), *M*_2_(*a*, *M*_1_(a*))) is not identifiable since it has two distinct counterfactual values of mediator *M*_1_, i.e., *M*_1_(*a*) and *M*_1_(a*), which means *M*_1_ is activated by two different values of *A* at the same time. Avin et al. [15] showed that such counterfactual formulas are not identifiable. We present identifiable decomposition components only with those identifiable counterfactual formulas of *Y*.

**Definition 3**. Natural MI effects in a causally sequential two-mediator scenario are defined as follows:

$${\text{NatINT}}_{AM_{1}}≔Y\left(a,M_{1}(a),M_{2}\left(a^{*},M_{1}(a)\right)\right)-Y\left(a^{*},M_{1}(a),M_{2}\left(a^{*},M_{1}(a)\right)\right)-Y\left(a,M_{1}\left(a^{*}\right),M_{2}\left(a^{*},M_{1}\left(a^{*}\right)\right)\right)+Y\left(a^{*},M_{1}\left(a^{*}\right),M_{2}\left(a^{*},M_{1}\left(a^{*}\right)\right)\right),$$

$${\text{NatINT}}_{AM_{2}}≔Y\left(a,M_{1}\left(a^{*}\right),M_{2}\left(a,M_{1}\left(a^{*}\right)\right)\right)-Y\left(a^{*},M_{1}\left(a^{*}\right),M_{2}\left(a,M_{1}\left(a^{*}\right)\right)\right)-Y\left(a,M_{1}\left(a^{*}\right),M_{2}\left(a^{*},M_{1}\left(a^{*}\right)\right)\right)+Y\left(a^{*},M_{1}\left(a^{*}\right),M_{2}\left(a^{*},M_{1}\left(a^{*}\right)\right)\right)\text{, }$$

$${\text{NatINT}}_{M_{1}M_{2}}≔Y\left(a^{*},M_{1}(a),M_{2}\left(a,M_{1}(a)\right)\right)-Y\left(a^{*},M_{1}\left(a^{*}\right),M_{2}\left(a,M_{1}\left(a^{*}\right)\right)\right)-Y\left(a^{*},M_{1}(a),M_{2}\left(a^{*},M_{1}(a)\right)\right)+Y\left(a^{*},M_{1}\left(a^{*}\right),M_{2}\left(a^{*},M_{1}\left(a^{*}\right)\right)\right),$$

$${\text{NatINT}}_{AM_{1}M_{2}}≔Y\left(a,M_{1}(a),M_{2}\left(a,M_{1}(a)\right)\right)-Y\left(a^{*},M_{1}(a),M_{2}\left(a,M_{1}(a)\right)\right)-Y\left(a,M_{1}\left(a^{*}\right),M_{2}\left(a,M_{1}\left(a^{*}\right)\right)\right)+Y\left(a^{*},M_{1}\left(a^{*}\right),M_{2}\left(a,M_{1}\left(a^{*}\right)\right)\right)-Y\left(a,M_{1}(a),M_{2}\left(a^{*},M_{1}(a)\right)\right)+Y\left(a^{*},M_{1}(a),M_{2}\left(a^{*},M_{1}(a)\right)\right)+Y\left(a,M_{1}\left(a^{*}\right),M_{2}\left(a^{*},M_{1}\left(a^{*}\right)\right)\right)-Y\left(a^{*},M_{1}\left(a^{*}\right),M_{2}\left(a^{*},M_{1}\left(a^{*}\right)\right)\right).$$

These interaction terms are similar to those in Definition 2 except that *M*_2_ has an extra input from *M*_1_. In ${\text{NatINT}}_{AM_{1}}$, *M*_2_ is neither fixed nor set at a level independent of *M*_1_; rather, *M*_2_ changes whenever *M*_1_ changes. Therefore, ${\text{NatINT}}_{AM_{1}}$ captures the change in the TE of *M*_1_ (going from *M*_1_(a*) to *M*_1_(*a*)) on the outcome when *A* goes from a* to *a*. In ${\text{NatINT}}_{M_{1}M_{2}}$, *M*_2_ would still partially depend on the level of *M*_1_. Hence, this component describes the interaction between *M*_1_ and *M*_2_ had *M*_2_ only change its exposure input. Similarly, the three-way interaction ${\text{NatINT}}_{AM_{1}M_{2}}$ can be interpreted as the change in the interaction between *A* and *M*_1_ when *M*_2_ has its exposure input going from a* to *a*.

We show in Supplementary material S3 that the TE can be decomposed into ten components at the individual level:

$$\text{TE}=\text{CDE}\left(m_{1}^{*},m_{2}^{*}\right)+{\text{INT}}_{\text{ref-}AM_{1}}\left(m_{1}^{*},m_{2}^{*}\right)+{\text{INT}}_{\text{ref-}AM_{2}}\left(m_{1}^{*},m_{2}^{*}\right)+{\text{INT}}_{\text{ref-}AM_{1}M_{2}}\left(m_{1}^{*},m_{2}^{*}\right)+{\text{NatINT}}_{AM_{1}}+{\text{NatINT}}_{AM_{2}}+{\text{NatINT}}_{AM_{1}M_{2}}+{\text{NatINT}}_{M_{1}M_{2}}+{\text{PIE}}_{M_{1}}+{\text{SNIE}}_{M_{2}}\text{,}$$

where

$$\text{CDE}\left(m_{1}^{*},m_{2}^{*}\right)=Y\left(a,m_{1}^{*},m_{2}^{*}\right)-Y\left(a^{*},m_{1}^{*},m_{2}^{*}\right),$$

$${\text{INT}}_{\text{ref-}AM_{1}}\left(m_{1}^{*},m_{2}^{*}\right)=Y\left(a,M_{1}\left(a^{*}\right),m_{2}^{*}\right)-Y\left(a^{*},M_{1}\left(a^{*}\right),m_{2}^{*}\right)-Y\left(a,m_{1}^{*},m_{2}^{*}\right)+Y\left(a^{*},m_{1}^{*},m_{2}^{*}\right),$$

$${\text{INT}}_{\text{ref-}AM_{2}}\left(m_{1}^{*},m_{2}^{*}\right)=Y\left(a,m_{1}^{*},M_{2}\left(a^{*},m_{1}^{*}\right)\right)-Y\left(a^{*},m_{1}^{*},M_{2}\left(a^{*},m_{1}^{*}\right)\right)-Y\left(a,m_{1}^{*},m_{2}^{*}\right)+Y\left(a^{*},m_{1}^{*},m_{2}^{*}\right),$$

$${\text{INT}}_{\text{ref-}AM_{1}M_{2}}\left(m_{1}^{*},m_{2}^{*}\right)=Y\left(a,M_{1}\left(a^{*}\right),M_{2}\left(a^{*},M_{1}\left(a^{*}\right)\right)\right)-Y\left(a^{*},M_{1}\left(a^{*}\right),M_{2}\left(a^{*},M_{1}\left(a^{*}\right)\right)\right)-Y\left(a,m_{1}^{*},M_{2}\left(a^{*},m_{1}^{*}\right)\right)+Y\left(a^{*},m_{1}^{*},M_{2}\left(a^{*},m_{1}^{*}\right)\right)-Y\left(a,M_{1}\left(a^{*}\right),m_{2}^{*}\right)+Y\left(a^{*},M_{1}\left(a^{*}\right),m_{2}^{*}\right)+Y\left(a,m_{1}^{*},m_{2}^{*}\right)-Y\left(a^{*},m_{1}^{*},m_{2}^{*}\right),$$

$${\text{PIE}}_{M_{1}}=Y\left(a^{*},M_{1}(a),M_{2}\left(a^{*},M_{1}(a)\right)\right)-Y\left(a^{*},M_{1}\left(a^{*}\right),M_{2}\left(a^{*},M_{1}\left(a^{*}\right)\right)\right),$$

$${\text{SNIE}}_{M_{2}}=Y\left(a^{*},M_{1}\left(a^{*}\right),M_{2}\left(a,M_{1}\left(a^{*}\right)\right)\right)-Y\left(a^{*},M_{1}\left(a^{*}\right),M_{2}\left(a^{*},M_{1}\left(a^{*}\right)\right)\right)\text{.}$$

**Figure 6:**
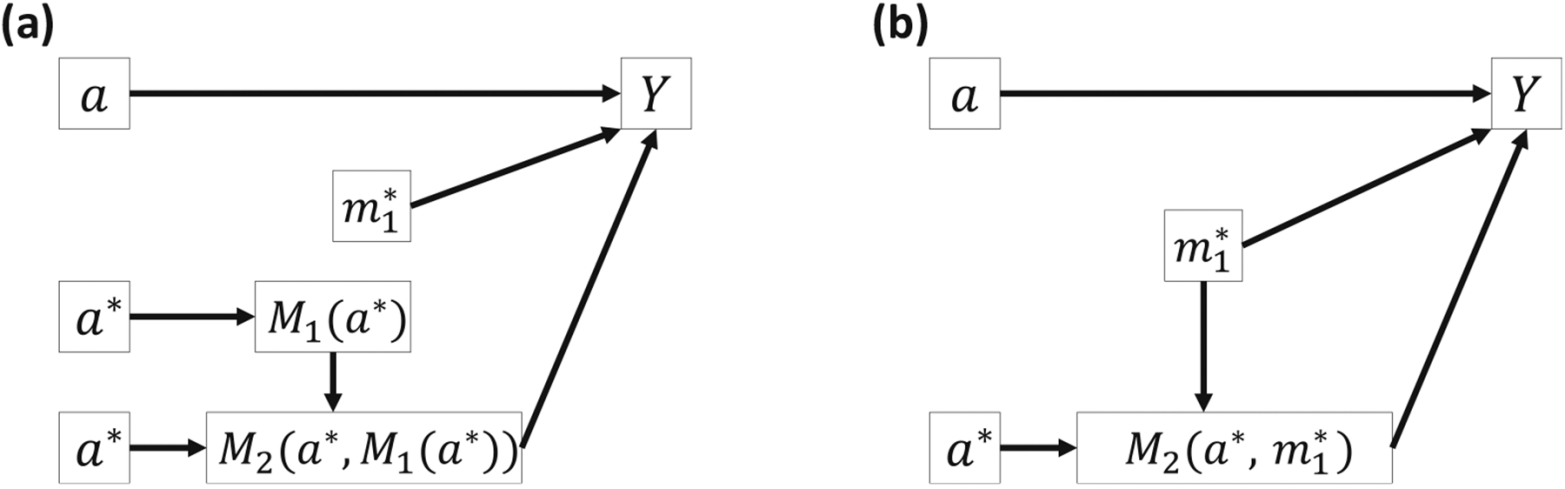
(a) The graphical illustration of $Y\left(a,m_{1}^{*},M_{2}\left(a^{*},M_{1}\left(a^{*}\right)\right)\right)$ which is an example of a type of non-identifiable counterfactual formula with *M*_1_ taking two different values, $m_{1}^{*}$ and *M*_1_(a*) in this example. (b) An identifiable counterfactual formula $Y\left(a,m_{1}^{*},M_{2}\left(a^{*},m_{1}^{*}\right)\right)$, where *M*_1_ takes one fixed value $m_{1}^{*}$.

**Figure 7:**
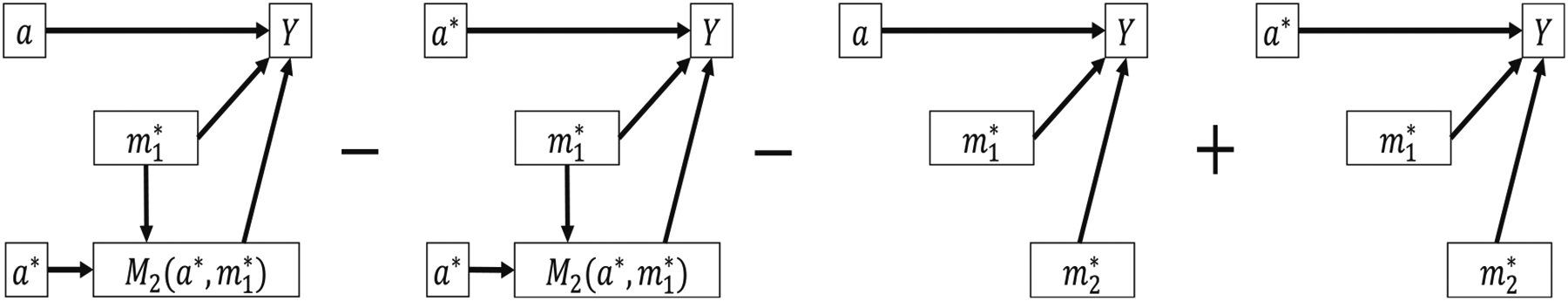
Graphical illustration of the reference interaction effect between *A* and *M*_2_ in a sequential two-mediator scenario, where *M*_1_ is fixed at the reference level $m_{1}^{*}$ so that the identifiability is ensured. *M*_2_ takes $M_{2}\left(a^{*},m_{1}^{*}\right)$ and $m_{2}^{*}$ as its treatment level and reference level, respectively.

Since the complexity significantly increases in a sequential two-mediator scenario with a direct causal link pointing from *M*_1_ to *M*_2_, a few important points need to be addressed. First, we need to ensure that all the counterfactual formulas in the decomposition are identifiable especially when finding ${\text{INT}}_{\text{ref-}AM_{2}}\left(m_{1}^{*},m_{2}^{*}\right)$ and ${\text{INT}}_{\text{ref-}AM_{1}M_{2}}\left(m_{1}^{*},m_{2}^{*}\right)$. We use the method from Figure 3 in Pearl [17] to graphically illustrate the counterfactual formulas. Figure 6a depicts $Y\left(a,m_{1}^{*},M_{2}\left(a^{*},M_{1}\left(a^{*}\right)\right)\right)$ as an example of a non-identifiable counterfactual formula and could be seen as a variant of the problematic counterfactual formulas proposed by Avin et al. [15]. We show how such counterfactual formulas might appear in ${\text{INT}}_{\text{ref-}AM_{2}}\left(m_{1}^{*},m_{2}^{*}\right)$ and ${\text{INT}}_{\text{ref-}AM_{1}M_{2}}\left(m_{1}^{*},m_{2}^{*}\right)$ and describe their non-identifiability in Supplementary material S4. Briefly, *M*_1_ can potentially take two different values within $Y\left(a,m_{1}^{*},M_{2}\left(a^{*},M_{1}\left(a^{*}\right)\right)\right)$, i.e., $m_{1}^{*}$ and *M*_1_(a*) can be different, which results in non-identifiability. In our approach to find ${\text{INT}}_{\text{ref-}AM_{2}}\left(m_{1}^{*},m_{2}^{*}\right)$, we set *M*_1_ to a fixed reference level $m_{1}^{*}$ and also use it as the second input argument of *M*_2_. With this approach, *M*_1_ only takes one value in each counterfactual formula of *Y* as illustrated in Figure 6b, and therefore the non-identifiability would not occur. A graphical illustration for the reference interaction effect between *A* and *M*_2_ is shown in Figure 7.

**Figure 8:**
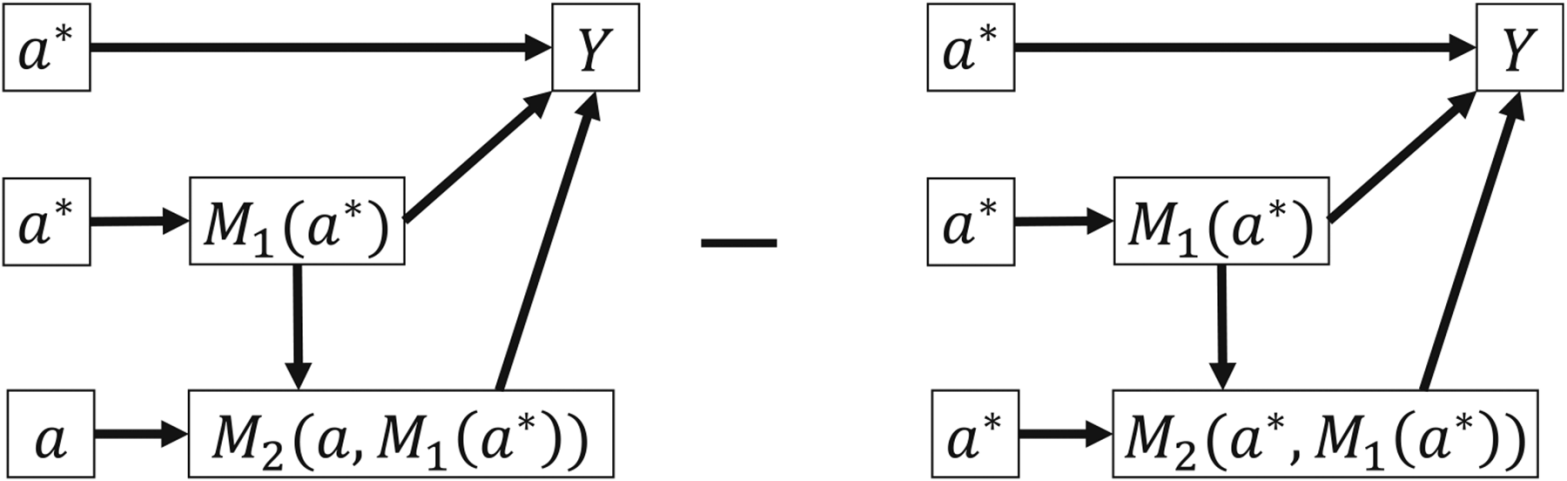
Graphical illustration of the seminatural indirect effect through *M*_2_, ${\text{SNIE}}_{M_{2}}$, which evaluates the causal effect along the path *A*→*M*_2_ →*Y* and can be interpreted as the effect due to partial mediation through *M*_2_ only.

Second, the causal effect along the path *A* → *M*_1_ → *M*_2_ → *Y* and the causal effect along the path *A* → *M*_2_ → *Y* combine to give the complete mediated effect through *M*_2_ (Figure 5). However, the part from *A* → *M*_1_ → *M*_2_ → *Y* is non-identifiable [15], and therefore we use the notion of seminatural indirect effect [19] instead of the PIE for the mediated effect through *M*_2_ in a sequential two-mediator scenario. The seminatural indirect effect through *M*_2_, ${\text{SNIE}}_{M_{2}}$, measures the causal effect along the path *A* → *M*_2_ → *Y* and can be interpreted as the effect due to partial mediation through *M*_2_ only [19,20]. A graphical illustration of ${\text{SNIE}}_{M_{2}}$ is presented in Figure 8.

These ten components and their interpretations are shown in Table 3 for the special case when *A*, *M*_1_, and *M*_2_ are all binary and additionally *a* = 1, a* = 0, $m_{1}^{*}=0$, and $m_{2}^{*}=0$.

## 4 Relations to traditional definitions

For both a non-sequential and a sequential two-mediator scenario, the ten components can be grouped into different portions with traditional definitions that are of great interest. In this section, we illustrate the relations of our proposed decompositions to the traditional definitions introduced in previous literature [7,16,17,21].

### 4.1 Non-sequential two-mediator scenario

Recall that the TE can be decomposed into the following ten components in a non-sequential two-mediator scenario:

$$\text{TE}=\text{CDE}\left(m_{1}^{*},m_{2}^{*}\right)+{\text{INT}}_{\text{ref-}AM_{1}}\left(m_{1}^{*},m_{2}^{*}\right)+{\text{INT}}_{\text{ref-}AM_{2}}\left(m_{1}^{*},m_{2}^{*}\right)+{\text{INT}}_{\text{ref-}AM_{1}M_{2}}\left(m_{1}^{*},m_{2}^{*}\right)+{\text{NatINT}}_{AM_{1}}+{\text{NatINT}}_{AM_{2}}+{\text{NatINT}}_{AM_{1}M_{2}}+{\text{NatINT}}_{M_{1}M_{2}}+{\text{PIE}}_{M_{1}}+{\text{PIE}}_{M_{2}}.$$

First, the sum of the CDE and the reference interaction effects equals the PDE that evaluates the causal effect through the direct path *A* → *Y* and is defined as the difference in the outcome when the exposure goes from a* to *a* while the mediators take their potential values, *M*_1_(a*) and *M*_2_(a*) [16]. Namely, we have,

(1) $$\text{PDE}=Y\left(a,M_{1}\left(a^{*}\right),M_{2}\left(a^{*}\right)\right)-Y\left(a^{*},M_{1}\left(a^{*}\right),M_{2}\left(a^{*}\right)\right)\;=\text{CDE}\left(m_{1}^{*},m_{2}^{*}\right)+{\text{INT}}_{\text{ref-}AM_{\text{1}}}\left(m_{1}^{*},m_{2}^{*}\right)+{\text{INT}}_{\text{ref-}AM_{2}}\left(m_{1}^{*},m_{2}^{*}\right)+{\text{INT}}_{\text{ref-}AM_{\text{1}}M_{2}}\left(m_{1}^{*},m_{2}^{*}\right).$$

Intuitively, the CDE and the reference interaction effects are the only components in the decomposition that do not require any mediated effects to exist as shown in equation (1). The four-way decomposition [7] also has the corresponding relation but the reference interaction effect only consists of one term.

The TDE [16] is different from PDE in the way that the potential values *M*_1_(*a*) and *M*_2_(*a*) are employed instead of *M*_1_(a*) and *M*_2_(a*). TDE can be expressed as the sum of four components consisting of PDE, ${\text{NatINT}}_{AM_{1}}$, ${\text{NatINT}}_{AM_{2}}$, and ${\text{NatINT}}_{AM_{1}M_{2}}$:

(2) $$\text{TDE}=Y\left(a,M_{1}(a),M_{2}(a)\right)-Y\left(a^{*},M_{1}(a),M_{2}(a)\right)\;=\text{PDE}+{\text{NatINT}}_{AM_{1}}+{\text{NatINT}}_{AM_{2}}+{\text{NatINT}}_{AM_{1}M_{2}}.$$

The natural MI effect between *M*_1_ and *M*_2_, ${\text{NatINT}}_{M_{1}M_{2}}$, is not included in equation (2). This is because ${\text{NatINT}}_{M_{1}M_{2}}$ measures the interdependence of the mediated effects through the two mediators while the exposure is fixed at a* for the direct path.

The NIE through *M*_1_, ${\text{NIE}}_{M_{1}}$, is defined by disabling the direct path with the fixed reference level a* as well as suppressing the indirect effect through *M*_2_ with the potential value *M*_2_(*a*), which can be seen as the type 2 mediator-specific effect proposed by Daniel et al. [3] without a direct causal link pointing from *M*_1_ to *M*_2_. We show in Supplementary material S1 that ${\text{NIE}}_{M_{1}}$ is the sum of ${\text{NatINT}}_{M_{1}M_{2}}$ and ${\text{PIE}}_{M_{1}}$, which can be expressed as the following equation:

$${\text{NIE}}_{M_{1}}=Y\left(a^{*},M_{1}(a),M_{2}(a)\right)-Y\left(a^{*},M_{1}\left(a^{*}\right),M_{2}(a)\right)={\text{NatINT}}_{M_{1}M_{2}}+{\text{PIE}}_{M_{1}}\text{,}$$

where ${\text{PIE}}_{M_{1}}$ satisfies the definition of a path-specific effect through *M*_1_ [17].

**Figure 9:**
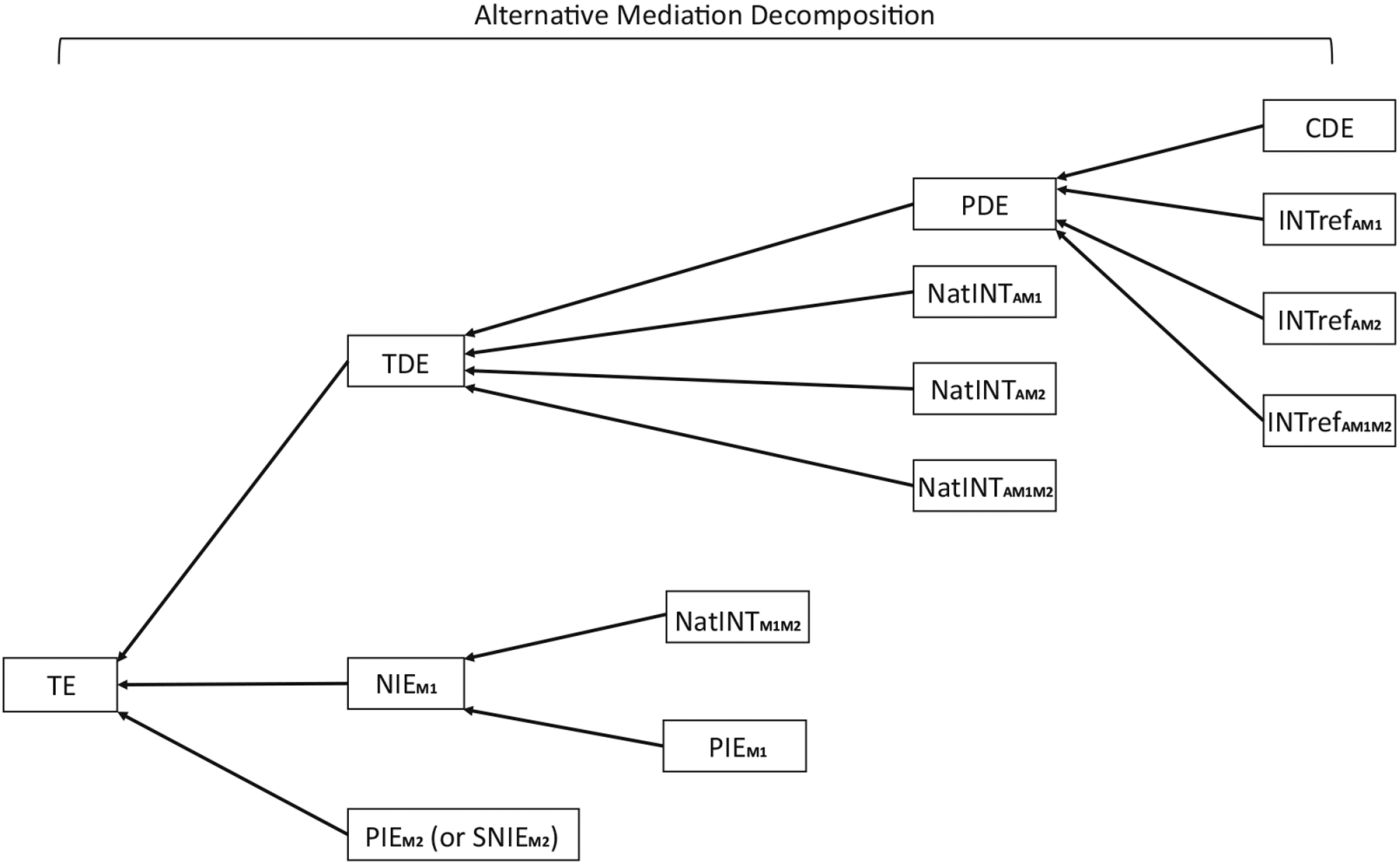
A flowchart illustrating an alternative mediation decomposition. For a non-sequential two-mediator scenario, the PDE consists of the CDE ($\text{CDE}\left(m_{1}^{*},m_{2}^{*}\right)$) and the reference interaction effects (INT_ref_s); the TDE consists of the PDE and the natural mediated interaction effects (NatINTs) except for the one between *M*_1_ and *M*_2_; the NIE through *M*_1_ (${\text{NIE}}_{M_{1}}$) consists of the PIE through *M*_1_ (${\text{PIE}}_{M_{1}}$) and the natural MI effect between *M*_1_ and *M*_2_ (${\text{NatINT}}_{M_{1}M_{2}}$); the TE consists of the TDE, the NIE through *M*_1_ (${\text{NIE}}_{M_{1}}$), and the PIE through *M*_2_ (${\text{PIE}}_{M_{2}}$). For a sequential two-mediator scenario, one can still follow the flowchart by replacing ${\text{PIE}}_{M_{2}}$ with ${\text{SNIE}}_{M_{2}}$.

The PIE through *M*_2_, ${\text{PIE}}_{M_{2}}$, is also a path specific effect. Figure 9 depicts an alternative mediation decomposition and illustrates the relations between the ten components and the traditional definitions in a non-sequential two-mediator scenario. Other relations that are not shown in Figure 9 can also be obtained. For example, the TIE [16] can be expressed as the sum of the following components:

$$\text{TIE}=Y\left(a,M_{1}(a),M_{2}(a)\right)-Y\left(a,M_{1}\left(a^{*}\right),M_{2}\left(a^{*}\right)\right)\;={\text{NatINT}}_{AM_{1}}+{\text{NatINT}}_{AM_{2}}+{\text{NatINT}}_{AM_{1}M_{2}}+{\text{NatINT}}_{M_{1}M_{2}}+{\text{PIE}}_{M_{1}}+{\text{PIE}}_{M_{2}}\text{,}$$

since

$$\text{TE-PDE}=Y\left(a,M_{1}(a),M_{2}(a)\right)-Y\left(a^{*},M_{1}\left(a^{*}\right),M_{2}\left(a^{*}\right)\right)-Y\left(a,M_{1}\left(a^{*}\right),M_{2}\left(a^{*}\right)\right)+Y\left(a^{*},M_{1}\left(a^{*}\right),M_{2}\left(a^{*}\right)\right)\;=Y\left(a,M_{1}(a),M_{2}(a)\right)-Y\left(a,M_{1}\left(a^{*}\right),M_{2}\left(a^{*}\right)\right)\;=\text{TIE}.$$

**Figure 10:**
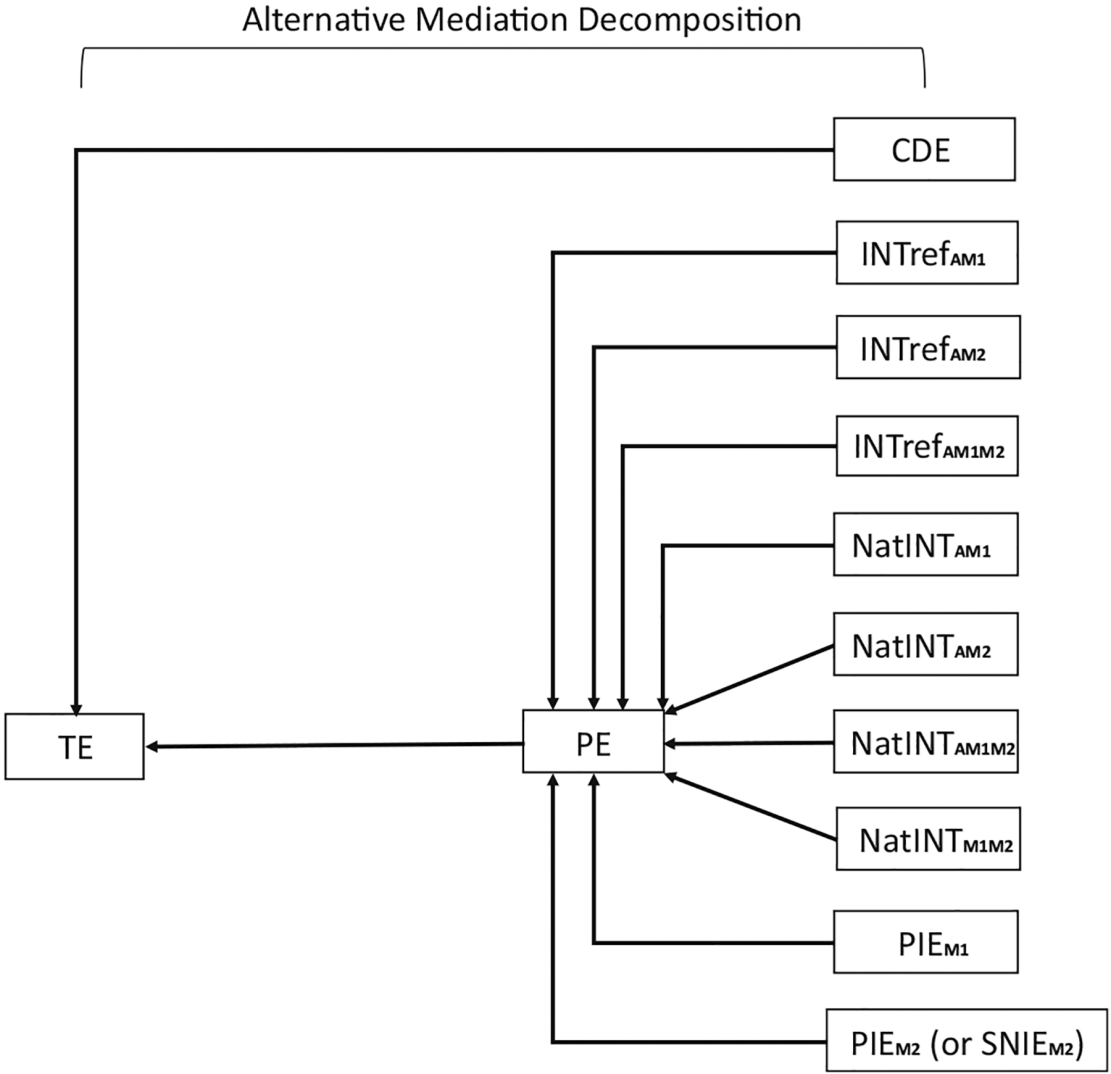
A flowchart illustrating an alternative mediation decomposition. For a non-sequential two-mediator scenario, the PE can be found by summing up the reference interaction effects (INT_ref_s), the natural mediated interaction effects (NatINTs), and the PIEs. The PE can also be calculated by subtracting the CDE ($\text{CDE}\left(m_{1}^{*},m_{2}^{*}\right)$) from the TE. For a sequential two-mediator scenario, one can still follow the flowchart by replacing ${\text{PIE}}_{M_{2}}$ with ${\text{SNIE}}_{M_{2}}$.

The portion eliminated (PE) is another useful measure that evaluates how much the causal effect of the exposure on the outcome would be removed if the mediators were set to 0 [16,21]. It can be expressed as follows:

$$\text{PE}=\text{TE -CDE}\left(m_{1}^{*},m_{2}^{*}\right)\;={\text{INT}}_{\text{ref-}AM_{1}}\left(m_{1}^{*},m_{2}^{*}\right)+{\text{INT}}_{\text{ref-}AM_{2}}\left(m_{1}^{*},m_{2}^{*}\right)+{\text{INT}}_{\text{ref-}AM_{1}M_{2}}\left(m_{1}^{*},m_{2}^{*}\right)+{\text{NatINT}}_{AM_{1}}+{\text{NatINT}}_{AM_{2}}+{\text{NatINT}}_{AM_{1}M_{2}}+{\text{NatINT}}_{M_{1}M_{2}}+{\text{PIE}}_{M_{1}}+{\text{PIE}}_{M_{2}}\text{,}$$

where the graphical illustration for this alternative decomposition with PE is shown in Figure 10.

If the components related to the effect due to interaction are of great interest, the portion attributable to interaction (PAI) [7] can be found by summing up the reference and natural MI effects. Namely, we have,

$$\text{PAI}={\text{INT}}_{\text{ref-}AM_{1}}\left(m_{1}^{*},m_{2}^{*}\right)+{\text{INT}}_{\text{ref-}AM_{2}}\left(m_{1}^{*},m_{2}^{*}\right)+{\text{INT}}_{\text{ref-}AM_{\text{1}}AM_{2}}\left(m_{1}^{*},m_{2}^{*}\right)+{\text{NatINT}}_{AM_{1}}+{\text{NatINT}}_{AM_{2}}+{\text{NatINT}}_{AM_{1}M_{2}}+{\text{NatINT}}_{M_{1}M_{2}},$$

which leads to a four-way decomposition for a non-sequential two-mediator scenario:

$$\text{TE}=\text{CDE}\left(m_{1}^{*},m_{2}*\right)+\text{PAI}+{\text{PIE}}_{M_{1}}+{\text{PIE}}_{M_{2}}.$$

**Figure 11:**
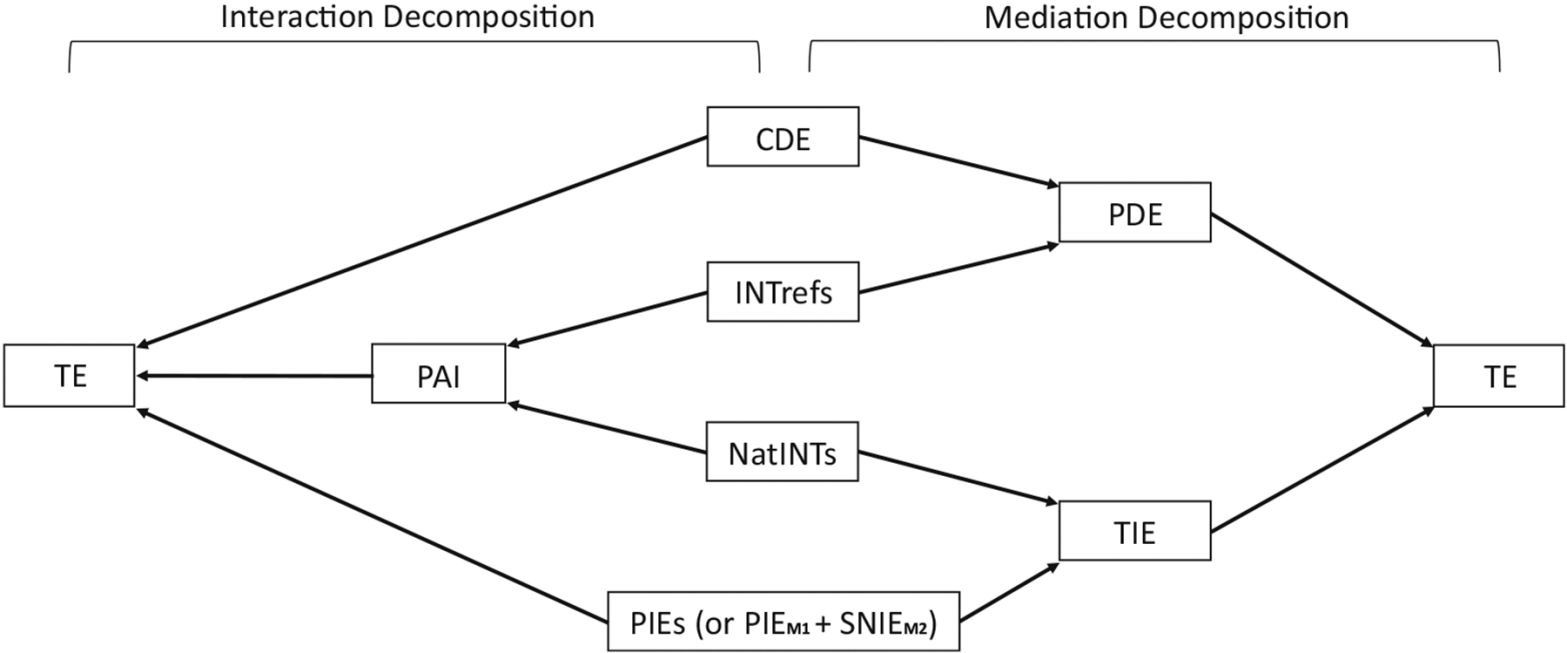
A flowchart illustrating alternative mediation and interaction decompositions. For a non-sequential two-mediator scenario, the left part shows an interaction decomposition. The portion attributable to interaction (PAI) consists of the reference interaction effects (INT_ref_s) and the natural mediated interaction effects (NatINTs). The TE consists of the CDE ($\text{CDE}\left(m_{1}^{*},m_{2}^{*}\right)$), the portion attributable to interaction (PAI), and the PIEs. The right part shows a mediation decomposition. The PDE consists of the CDE ($\text{CDE}\left(m_{1}^{*},m_{2}^{*}\right)$) and the reference interaction effects (INT_ref_s). The TIE consists of the NatINTs and the PIEs. The TE consists of the PDE and the TIE. For a sequential two-mediator scenario, one can still follow the flowchart by replacing ${\text{PIE}}_{M_{2}}$ with ${\text{SNIE}}_{M_{2}}$.

Figure 11 presents an overall picture for the interaction and mediation decompositions with the ten components for a non-sequential two-mediator scenario. Suggested choices for the multiway interaction decompositions are summarized in Table 4.

### 4.2 Sequential two-mediator scenario

We recall the ten components of the decomposed TE for a sequential two-mediator scenario:

$$\text{TE}=\text{CDE}\left(m_{1}^{*},m_{2}^{*}\right)+{\text{INT}}_{\text{ref-}AM_{1}}\left(m_{1}^{*},m_{2}^{*}\right)+{\text{INT}}_{\text{ref-}AM_{2}\;}\left(m_{1}^{*},m_{2}^{*}\right)+{\text{INT}}_{\text{ref-}AM_{1}M_{2}}\left(m_{1}^{*},m_{2}^{*}\right)+{\text{NatINT}}_{AM_{1}}+{\text{NatINT}}_{AM_{2}}+{\text{NatINT}}_{AM_{1}M_{2}}+{\text{NatINT}}_{M_{1}M_{2}}+{\text{PIE}}_{M_{1}}+{\text{SNIE}}_{M_{2}}.$$

As discussed in Section 3.2, the complete mediated effect through *M*_2_ cannot be identified with non-parametric models because of the direct causal link pointing from *M*_1_ to *M*_2_, and hence the seminatural indirect effect through *M*_2_, ${\text{SNIE}}_{M_{2}}$, is used instead. One can also employ traditional definitions to perform alternative interaction and mediation decompositions for a sequential two-mediator scenario by replacing ${\text{PIE}}_{M_{2}}$ with ${\text{SNIE}}_{M_{2}}$.

## 5 Identification assumptions and empirical formulas

The decompositions for one- and two-mediator scenarios thus far have been primarily conceptual. The individual-level effects in the decompositions cannot be identified from data, but under certain assumptions on confounding the population-averages of those components can be identified from data [6].

### 5.1 Identification assumptions

We first consider a single-mediator scenario. Four identification assumptions are required [22], which are listed below as (A′1)–(A′4):

(A′1) Y(a,m)⊥A∣C,

(A′2) Y(a,m)⊥M∣{A,C},

(A′3) M(a)⊥A∣C,

(A′4) $$Y(a,m)⊥M\left(a^{*}\right)|C,$$

where *C* is a set of covariates. The assumptions above state that given a covariate set *C* or {*A*, *C*}, there exist no unmeasured variables confounding the association between exposure *A* and outcome *Y* (A′1), no unmeasured variables confounding the association between mediator *M* and outcome *Y* (A′2), and no unmeasured variables confounding the association between exposure *A* and mediator *M* (A′3) [8]. (A′4) is a strong assumption and a few researchers published their works on this topic [4,7,23]. It could be interpreted as there exist no variables that are causal descendants of exposure *A*, and in the meantime, that confound the association between mediator *M* and outcome *Y* [4,17].

The analogs of (A′1)–(A′4) for a directed acyclic graph with two sequential mediators can be found by first considering *M*_1_ and *M*_2_ as a set [4]. Namely, we have four corresponding identification assumptions (A1)–(A4):

(A1) $$Y\left(a,m_{1},m_{2}\right)⊥A|C,$$

(A2) $$Y\left(a,m_{1},m_{2}\right)⊥\left\{M_{1},M_{2}\right\}|\left\{A,C\right\},$$

(A3) $$\left\{M_{1}(a),M_{2}\left(a,m_{1}\right)\right\}⊥A|C,$$

(A4) $$Y\left(a,m_{1},m_{2}\right)⊥\left\{M_{1}\left(a^{*}\right),M_{2}\left(a^{**},m_{1}\right)\right\}|C.$$

Similarly, the assumptions above state that given a covariate set *C* or {*A*, *C*}, there exist no unmeasured variables confounding the association between exposure *A* and outcome *Y* (A1), no unmeasured variables confounding the association between the mediator set {*M*_1_, *M*_2_} and outcome *Y* (A2), no unmeasured variables confounding the association between exposure *A* and the mediator set {*M*_1_, *M*_2_} (A3), and no unmeasured variables that are causal descendants of exposure *A*, and in the meantime, that confound the association between the mediator set {*M*_1_, *M*_2_} and outcome *Y* (A4) [4,22].

In order to account for the confounding between *M*_1_ and *M*_2_, two more assumptions are required:

(A5) $$M_{2}\left(a,m_{1}\right)⊥M_{1}|\left\{A,C\right\},$$

(A6) $$M_{2}\left(a,m_{1}\right)⊥M_{1}\left(a^{*}\right)|C,$$

where (A5) and (A6) state, respectively, that there exist no unmeasured variables confounding the association between *M*_1_ and *M*_2_ given {*A*, *C*}, and no unmeasured variables that are causal descendants of exposure *A*, and in the meantime, are confounding the association between *M*_1_ and *M*_2_ [4].

Steen et al. [4] presented comprehensive identification assumptions for the causal structures with multiple mediators and pointed out that weaker identification assumptions than (A1)–(A6) can be considered under certain decompositions.

### 5.2 Empirical formulas

Suppose a set of covariates *C* satisfies the assumptions on confounding for a decomposition. We can obtain the expected value of each component in the decomposition using the iterated conditional expectation rule. We focus on the scenario with two causally sequential mediators. Suppose *M*_1_ and *M*_2_ are categorical and let $p_{am_{1}m_{2}c}=E\left[Y|A=a,M_{1}=m_{1},M_{2}=m_{2},C=c\right]$. The following formulas can be obtained:

$$E\left[\text{CDE}\left(m_{1}^{*},m_{2}^{*}\right)|c\right]=p_{am_{1}^{*}m_{2}^{*}c}-p_{a^{*}m_{1}^{*}m_{2}^{*}c}$$

$$E\left[{\text{INT}}_{\text{ref}-AM_{1}}\left(m_{1}^{*},m_{2}^{*}\right)|c\right]=\underset{m_{1}}{\sum }\left(p_{am_{1}m_{2}^{*}c}-p_{am_{1}^{*}m_{2}^{*}c}-p_{a^{*}m_{1}m_{2}^{*}c}+p_{a^{*}m_{1}^{*}m_{2}^{*}c}\right)\times \text{Pr}\left(M_{1}=m_{1}|a^{*},c\right)$$

$$E\left[{\text{INT}}_{\text{ref-}AM_{2}}\left(m_{1}^{*},m_{2}^{*}\right)|c\right]=\underset{m_{2}}{\sum }\underset{m_{1}}{\sum }[\left(p_{am_{1}^{*}m_{2}c}-p_{a^{*}m_{1}^{*}m_{2}c}\right)\times \text{Pr}\left(M_{2}=m_{2}|a^{*},m_{1}^{*},c\right)+\left(-p_{am_{1}^{*}m_{2}^{*}c}+p_{a^{*}m_{1}^{*}m_{2}^{*}c}\right)\times \text{Pr}\left(M_{2}=m_{2}|a^{*},m_{1},c\right)]\times \text{Pr}\left(M_{1}=m_{1}|a^{*},c\right)$$

$$E\left[{\text{INT}}_{\text{ref}-AM_{1}M_{2}}\left(m_{1}^{*},m_{2}^{*}\right)|c\right]=\underset{m_{2}}{\sum }\underset{m_{1}}{\sum }[\left(p_{am_{1}m_{2}c}-p_{a^{*}m_{1}m_{2}c}-p_{am_{1}m_{2}^{*}c}+p_{a^{*}m_{1}m_{2}^{*}c}+p_{am_{1}^{*}m_{2}^{*}c}-p_{a^{*}m_{1}^{*}m_{2}^{*}c}\right)\times \text{Pr}\left(M_{2}=m_{2}|a^{*},m_{1},c\right)+\left(-p_{am_{1}^{*}m_{2}c}+p_{a^{*}m_{1}^{*}m_{2}c}\right)\times \text{Pr}\left(M_{2}=m_{2}|a^{*},m_{1}^{*},c\right)]\times \text{Pr}\left(M_{1}=m_{1}|a^{*},c\right)$$

$$E\left[{\text{NatINT}}_{AM_{1}}|c\right]=\underset{m_{2}}{\sum }\underset{m_{1}}{\sum }\left(p_{am_{1}m_{2}c}-p_{a^{*}m_{1}m_{2}c}\right)\times \text{Pr}\left(M_{2}=m_{2}|a^{*},m_{1},c\right)\times \left[\text{Pr}\left(M_{1}=m_{1}|a,c\right)-\text{Pr}\left(M_{1}=m_{1}|a^{*},c\right)\right]$$

$$E\left[{\text{NatINT}}_{AM_{2}}|c\right]=\underset{m_{2}}{\sum }\underset{m_{1}}{\sum }\left(p_{am_{1}m_{2}c}-p_{a^{*}m_{1}m_{2}c}\right)\times \text{Pr}\left(M_{1}=m_{1}|a^{*},c\right)\times \left[\text{Pr}\left(M_{2}=m_{2}|a,m_{1},c\right)-\text{Pr}\left(M_{2}=m_{2}|a^{*},m_{1},c\right)\right]$$

$$E\left[{\text{NatINT}}_{AM_{1}M_{2}}|c\right]=\underset{m_{2}}{\sum }\underset{m_{1}}{\sum }\left(p_{am_{1}m_{2}c}-p_{a^{*}m_{1}m_{2}c}\right)\times \left[\text{Pr}\left(M_{1}=m_{1}|a,c\right)-\text{Pr}\left(M_{1}=m_{1}|a^{*},c\right)\right]\times \left[\text{Pr}\left(M_{2}=m_{2}|a,m_{1},c\right)-\text{Pr}\left(M_{2}=m_{2}|a^{*},m_{1},c\right)\right]$$

$$E\left[{\text{NatINT}}_{M_{1}M_{2}}|c\right]=\underset{m_{2}}{\sum }\underset{m_{1}}{\sum }p_{a^{*}m_{1}m_{2}c}\left[\text{Pr}\left(M_{1}=m_{1}|a,c\right)-\text{Pr}\left(M_{1}=m_{1}|a^{*},c\right)\right][\text{Pr}\left(M_{2}=m_{2}|a,m_{1},c\right)-\text{Pr}\left(M_{2}=m_{2}|a^{*},m_{1},c\right)]$$

$$E\left[{\text{PIE}}_{M_{1}}|c\right]=\underset{m_{2}}{\sum }\underset{m_{1}}{\sum }p_{a^{*}m_{1}m_{2}c}\text{Pr}\left(M_{2}=m_{2}|a^{*},m_{1},c\right)\times \left[\text{Pr}\left(M_{1}=m_{1}|a,c\right)-\text{Pr}\left(M_{1}=m_{1}|a^{*},c\right)\right]$$

$$E\left[{\text{SNIE}}_{M_{2}}|c\right]=\underset{m_{2}}{\sum }\underset{m_{1}}{\sum }p_{a^{*}m_{1}m_{2}c}\times \text{Pr}\left(M_{1}=m_{1}|a^{*},c\right)\times [\text{Pr}\left(M_{2}=m_{2}|a,m_{1},c\right)-\text{Pr}\left(M_{2}=m_{2}|a^{*},m_{1},c\right)].$$

When *M*_1_ and *M*_2_ are continuous, empirical formulas can be obtained by replacing the sums by integrations and the conditional probabilities by conditional densities.

### 5.3 Relations to linear models

Suppose *Y*, *M*_1_, and *M*_2_ are continuous. For the scenario with two causally sequential mediators, we assume that the following regression models for *Y*, *M*_1_, and *M*_2_ are specified:

$$E\left[Y|A,M_{1},M_{2},C\right]=\theta_{0}+\theta_{1}A+\theta_{2}M_{1}+\theta_{3}M_{2}+\theta_{4}AM_{1}+\theta_{5}AM_{2}+\theta_{6}M_{1}M_{2}+\theta_{7}AM_{1}M_{2}+\theta_{8}^{\prime }C,$$

$$E\left[M_{2}|A,M_{1},C\right]=\beta_{0}+\beta_{1}A+\beta_{2}M_{1}+\beta_{3}AM_{1}+\beta_{4}^{\prime }C,$$

$$E\left[M_{1}|A,C\right]=\gamma_{0}+\gamma_{1}A+\gamma_{2}^{\prime }C,$$

where *C* is a confounding set that satisfies the identification assumptions (A1)–(A6). The expected values of the effect components are as follows:

$$E\left[\text{CDE}\left(m_{1}^{*},m_{2}^{*}\right)|c\right]=\left(\theta_{1}+\theta_{4}m_{1}^{*}+\theta_{5}m_{2}^{*}+\theta_{7}m_{1}^{*}m_{2}^{*}\right)\left(a-a^{*}\right)$$

$$E\left[{\text{INT}}_{\text{ref}-AM_{1}}\left(m_{1}^{*},m_{2}^{*}\right)|c\right]=\left(\gamma_{0}+\gamma_{1}a^{*}+\gamma_{2}^{\prime }c-m_{1}^{*}\right)\left(\theta_{4}+\theta_{7}m_{2}^{*}\right)\left(a-a^{*}\right)$$

$$E\left[{\text{INT}}_{\text{ref-}AM_{2}}\left(m_{1}^{*},m_{2}^{*}\right)|c\right]=\left(\theta_{5}+\theta_{7}m_{1}^{*}\right)\left(\beta_{0}+\beta_{1}a^{*}+\beta_{2}m_{1}^{*}+\beta_{3}a^{*}m_{1}^{*}+\beta_{4}^{\prime }c-m_{2}^{*}\right)\left(a-a^{*}\right)$$

$$E\left[{\text{INT}}_{\text{ref-}AM_{1}M_{2}}\left(m_{1}^{*},m_{2}^{*}\right)|c\right]=\{\theta_{1}+\theta_{7}\left(\beta_{0}+\beta_{1}a^{*}+\beta_{4}^{\prime }c\right)\left(\gamma_{0}+\gamma_{1}a^{*}+\gamma_{2}^{\prime }c\right)+\theta_{5}\left(\beta_{2}+\beta_{3}a^{*}\right)\left(\gamma_{0}+\gamma_{1}a^{*}+\gamma_{2}^{\prime }c\right)+\theta_{7}\left(\beta_{2}+\beta_{3}a^{*}\right)\left[\sigma_{M_{1}}^{2}+{\left(\gamma_{0}+\gamma_{1}a^{*}+\gamma_{2}^{\prime }c\right)}^{2}\right]-\left(\theta_{1}+\theta_{5}m_{2}^{*}\right)-\theta_{7}m_{2}^{*}\left(\gamma_{0}+\gamma_{1}a^{*}+\gamma_{2}^{\prime }c\right)-\theta_{5}\left(\beta_{2}m_{1}^{*}+\beta_{3}a^{*}m_{1}^{*}-m_{2}^{*}\right)-\theta_{7}m_{1}^{*}\left(\beta_{0}+\beta_{1}a^{*}+\beta_{2}m_{1}^{*}+\beta_{3}a^{*}m_{1}^{*}+\beta_{4}^{\prime }c-m_{2}^{*}\right)\}\left(a-a^{*}\right)$$

$$E\left[{\text{NatINT}}_{AM_{1}}|c\right]=[\theta_{4}\gamma_{1}+\theta_{7}\gamma_{1}\left(\beta_{0}+\beta_{1}a^{*}+\beta_{4}^{\prime }c\right)+\theta_{5}\gamma_{1}\left(\beta_{2}+\beta_{3}a^{*}\right)+2\theta_{7}\gamma_{1}\left(\beta_{2}+\beta_{3}a^{*}\right)\left(\gamma_{0}+\gamma_{2}^{\prime }c\right)+\theta_{7}\gamma_{1}^{2}\left(\beta_{2}+\beta_{3}a^{*}\right)\left(a+a^{*}\right)]{\left(a-a^{*}\right)}^{2}$$

$$E\left[{\text{NatINT}}_{AM_{2}}|c\right]=[\theta_{5}\beta_{1}+\theta_{7}\beta_{1}\left(\gamma_{0}+\gamma_{1}a^{*}+\gamma_{2}^{\prime }c\right)+\theta_{5}\beta_{3}\left(\gamma_{0}+\gamma_{1}a^{*}+\gamma_{2}^{\prime }c\right)+\theta_{7}\beta_{3}\left[\sigma_{M_{1}}^{2}+{\left(\gamma_{0}+\gamma_{1}a^{*}+\gamma_{2}^{\prime }c\right)}^{2}\right]]{\left(a-a^{*}\right)}^{2}$$

$$E\left[{\text{NatINT}}_{AM_{1}M_{2}}|c\right]=\left[\theta_{7}\beta_{1}\gamma_{1}+\theta_{5}\beta_{3}\gamma_{1}+2\theta_{7}\beta_{3}\gamma_{1}\left(\gamma_{0}+\gamma_{2}^{\prime }c\right)+\theta_{7}\beta_{3}\gamma_{1}^{2}\left(a+a^{*}\right)\right]{\left(a-a^{*}\right)}^{3}$$

$$E\left[{\text{NatINT}}_{M_{1}M_{2}}|c\right]=[\beta_{1}\gamma_{1}\left(\theta_{6}+\theta_{7}a^{*}\right)+\beta_{3}\gamma_{1}\left(\theta_{3}+\theta_{5}a^{*}\right)+2\beta_{3}\gamma_{1}\left(\theta_{6}+\theta_{7}a^{*}\right)\left(\gamma_{0}+\gamma_{2}^{\prime }c\right)+\beta_{3}\gamma_{1}^{2}\left(\theta_{6}+\theta_{7}a^{*}\right)\left(a+a^{*}\right)]{\left(a-a^{*}\right)}^{2}$$

$$E\left[{\text{PIE}}_{M_{1}}|c\right]=[\gamma_{1}\left(\theta_{2}+\theta_{4}a^{*}\right)+\gamma_{1}\left(\theta_{6}+\theta_{7}a^{*}\right)\left(\beta_{0}+\beta_{1}a^{*}+\beta_{4}^{\prime }c\right)+\gamma_{1}\left(\theta_{3}+\theta_{5}a^{*}\right)\left(\beta_{2}+\beta_{3}a^{*}\right)+2\gamma_{1}\left(\theta_{6}+\theta_{7}a^{*}\right)\left(\beta_{2}+\beta_{3}a^{*}\right)\left(\gamma_{0}+\gamma_{2}^{\prime }c\right)+\gamma_{1}^{2}\left(\theta_{6}+\theta_{7}a^{*}\right)\left(\beta_{2}+\beta_{3}a^{*}\right)\left(a+a^{*}\right)]\left(a-a^{*}\right)$$

$$E\left[{\text{SNIE}}_{M_{2}}|c\right]=[\beta_{1}\left(\theta_{3}+\theta_{5}a^{*}\right)+\beta_{1}\left(\theta_{6}+\theta_{7}a^{*}\right)\left(\gamma_{0}+\gamma_{1}a^{*}+\gamma_{2}^{\prime }c\right)+\beta_{3}\left(\theta_{3}+\theta_{5}a^{*}\right)\left(\gamma_{0}+\gamma_{1}a^{*}+\gamma_{2}^{\prime }c\right)+\beta_{3}\left(\theta_{6}+\theta_{7}a^{*}\right)\left[\sigma_{M_{1}}^{2}+{\left(\gamma_{0}+\gamma_{1}a^{*}+\gamma_{2}^{\prime }c\right)}^{2}\right]]\left(a-a^{*}\right)$$

$$E[\text{TE}|c]=[\theta_{1}+\theta_{5}\left(\beta_{0}+\beta_{4}^{\prime }c\right)+\beta_{1}\theta_{3}+\theta_{4}\left(\gamma_{0}+\gamma_{2}^{\prime }c\right)+\gamma_{1}\theta_{2}+\theta_{7}\left(\beta_{0}+\beta_{4}^{\prime }c\right)\left(\gamma_{0}+\gamma_{2}^{\prime }c\right)+\beta_{1}\theta_{6}\left(\gamma_{0}+\gamma_{2}^{\prime }c\right)+\gamma_{1}\theta_{6}\left(\beta_{0}+\beta_{4}^{\prime }c\right)+\theta_{5}\beta_{2}\left(\gamma_{0}+\gamma_{2}^{\prime }\right)+\theta_{3}\beta_{3}\left(\gamma_{0}+\gamma_{2}^{\prime }c\right)+\theta_{3}\beta_{2}\gamma_{1}+\theta_{7}\beta_{2}\sigma_{M_{1}}^{2}+\theta_{6}\beta_{3}\sigma_{M_{1}}^{2}+\theta_{7}\beta_{2}{\left(\gamma_{0}+\gamma_{2}^{\prime }c\right)}^{2}+\theta_{6}\beta_{3}{\left(\gamma_{0}+\gamma_{2}^{\prime }c\right)}^{2}+2\gamma_{1}\theta_{6}\beta_{2}\left(\gamma_{0}+\gamma_{2}^{\prime }c\right)]\left(a-a^{*}\right)+[\beta_{1}\theta_{5}+\gamma_{1}\theta_{4}+\beta_{1}\theta_{7}\left(\gamma_{0}+\gamma_{2}^{\prime }c\right)+\gamma_{1}\theta_{7}\left(\beta_{0}+\beta_{4}^{\prime }c\right)+\gamma_{1}\beta_{1}\theta_{6}+\theta_{5}\beta_{3}\left(\gamma_{0}+\gamma_{2}^{\prime }c\right)+\theta_{5}\beta_{2}\gamma_{1}+\theta_{3}\beta_{3}\gamma_{1}+\theta_{7}\beta_{3}\sigma_{M_{1}}^{2}+\theta_{7}\beta_{3}{\left(\gamma_{0}+\gamma_{2}^{\prime }c\right)}^{2}+2y_{1}\theta_{7}\beta_{2}\left(\gamma_{0}+\gamma_{2}^{\prime }c\right)+2\gamma_{1}\theta_{6}\beta_{3}\left(\gamma_{0}+\gamma_{2}^{\prime }c\right)+\theta_{6}\beta_{2}\gamma_{1}^{2}]\left(a^{2}-a^{*2}\right)+[\gamma_{1}\beta_{1}\theta_{7}+\theta_{5}\beta_{3}\gamma_{1}+2\gamma_{1}\theta_{7}\beta_{3}\left(\gamma_{0}+\gamma_{2}^{\prime }c\right)+\theta_{7}\beta_{2}\gamma_{1}^{2}+\theta_{6}\beta_{3}\gamma_{1}^{2}]\left(a^{3}-a^{*3}\right)+\theta_{7}\beta_{3}\gamma_{1}^{2}\left(a^{4}-a^{*4}\right),$$

where $\sigma_{M_{1}}^{2}$ denotes the constant variance of random error term for *M*_1_. A complete derivation for the aforementioned formulas are presented in Supplementary material S5.

For a scenario with two causally non-sequential mediators, again we assume that a set of covariates *C* satisfies the identification assumptions for the decomposition and that the following regression models for *Y*, *M*_1_, and *M*_2_ are specified:

$$E\left[Y|A,M_{1},M_{2},C\right]=\theta_{0}+\theta_{1}A+\theta_{2}M_{1}+\theta_{3}M_{2}+\theta_{4}AM_{1}+\theta_{5}AM_{2}+\theta_{6}M_{1}M_{2}+\theta_{7}AM_{1}M_{2}+\theta_{8}^{\prime }C,$$

$$E\left[M_{2}|A,C\right]=\beta_{0}+\beta_{1}A+\beta_{4}^{\prime }C,$$

$$E\left[M_{1}|A,C\right]=\gamma_{0}+\gamma_{1}A+\gamma_{2}^{\prime }C.$$

The results can be obtained as a special case of those derived from the scenario with two causally sequential mediators by setting parameters *β*_2_ and *β*_3_ to zero. Table 5 presents a side-by-side comparison of the expected value of six selected components in our proposed decomposition that are potentially different from the mediated effects in the study by Bellavia and Valeri [9]. Formulas are derived under linear structural equation models in a non-sequential two-mediator scenario with continuous outcome and mediators. Both decompositions have identical CDE and reference interaction effects. It was noted that the mediated effects in Bellavia and Valeri depend on two arbitrarily chosen values for *M*_1_(a*) and *M*_2_(a*), respectively. For example, the expected value of MI effect between *A* and *M*_1_ can be expressed as follows:

$$E\left[{\text{INTmed}}_{AM_{1}}|M_{2}\left(a^{*}\right)=m_{2}^{*},c\right]=\left(\theta_{4}+\theta_{7}m_{2}^{*}\right)\gamma_{1}{\left(a-a^{*}\right)}^{2},$$

where $m_{2}^{*}$ is an arbitrarily chosen value for *M*_2_(a*).

Compared to $E\left[{\text{INTmed}}_{AM_{1}}\right]$, the expected value of natural MI effect between *A* and *M*_1_ is given as follows:

$$E\left[{\text{NatINT}}_{AM_{1}}|c\right]=\left[\theta_{4}+\theta_{7}\left(\beta_{0}+\beta_{1}a^{*}+\beta_{4}^{\prime }c\right)\right]\gamma_{1}{\left(a-a^{*}\right)}^{2}.$$

The key difference is that $E\left[{\text{NatINT}}_{AM_{1}}\right]$ does not assume any arbitrarily chosen value for *M*_2_(a*) but uses the population averaged value of *M*_2_(a*) in the linear model which is $\beta_{0}+\beta_{1}a^{*}+\beta_{4}^{\prime }c$. Hence, $E\left[{\text{NatINT}}_{AM_{1}}\right]$ provides a natural interpretation of the MI between *A* and *M*_1_.

## 6 Illustrations with simulated and real data

We use a simulated data set to compare our method to Bellavia’s and Valeri’s method [9] in a non-sequential two-mediator scenario. We also analyzed a real data set in a sequential two-mediator scenario using the formulas derived in Section 5.3 for illustration.

### 6.1 Illustration with a simulated data set in a non-sequential two-mediator scenario

To compare Bellavia’s and Valeri’s method and our proposed decomposition with two non-sequential mediators (Figure 3), we simulated *n* = 1,000 observations from the following linear structural equation models:

$$E\left[Y|A,M_{1},M_{2},C\right]=0.2+0.3A+0.3M_{1}+0.4M_{2}+0.01AM_{1}+0.02AM_{2}+0.6M_{1}M_{2}+0.7AM_{1}M_{2}+0.2C,$$

$$E\left[M_{2}|A,C\right]=0.2+0.3A+0.2C,$$

$$E\left[M_{1}|A,C\right]=0.2+0.3A+0.2C,$$

where the exposure *A*, mediators *M*_1_ and *M*_2_, outcome *Y*, and covariate *C* are all continuous random variables.

The covariate *C* is the only confounder for the associations among *A*, *M*_1_, *M*_2_, and *Y* and was randomly drawn from *N*(0.2, 0.5), where 0.5 is the standard deviation. We randomly drew the exposure *A* from *N*(0.3 + 3*c*, 0.5), *M*_1_ and *M*_2_ from *N*(0.2 + 0.3*a* + 0.2*c*, 0.5), and *Y* from *N*(0.2 + 0.3*a* + 0.3*m*_1_ + 0.4*m*_2_ + 0.01*am*_1_ + 0.02*am*_2_ + 0.6*m*_1_*m*_2_ + 0.7*am*_1_*m*_2_ + 0.2*c*, 0.5).

The treatment and reference level of *A* are *a* = 1 and a* = 0, respectively. The fixed reference levels of *M*_1_ and *M*_2_, $m_{1}^{*}$ and $m_{2}^{*}$, were set to 0 in calculating the CDE, reference interaction effects, and the mediated effects in Bellavia’s and Valeri’s method. We plugged in the maximum likelihood estimators for the coefficients and unbiased estimator for the constant variance into the regression-based formulas to obtain point estimates of the effects in the decompositions and used 100,000 bootstrap samples to obtain the 95% confidence intervals [24]. Table 6 shows the simulation results and interpretations of the identical components, including the CDE, reference interaction effects, PDE, and TE. Table 7 presents the simulation results of other decomposition components that are expected to be different.

Bellavia’s and Valeri’s method has a few drawbacks. First of all, the mediated effects in Bellavia’s and Valeri’s method vary with respect to the arbitrary choices of $m_{1}^{*}$ and $m_{2}^{*}$. Second, the interpretations of the mediated effects in Bellavia and Valeri have to account for the choices of $m_{1}^{*}$ and $m_{2}^{*}$, and therefore have a lack of generalizability (Table 8). At last, it is difficult to extend Bellavia’s and Valeri’s method into the scenarios with multiple sequential mediators by fixing the mediators at certain levels. For example, in a sequential two-mediator scenario (Figure 5), the direct causal link pointing from *M*_1_ to *M*_2_ would have to be removed by setting *M*_2_ to a fixed value. Namely, the causal relationship between *M*_1_ and *M*_2_ in a sequential two-mediator scenario would be lost. In contrast, our proposed decomposition overcomes these disadvantages by allowing the mediators to naturally vary with respect to the exposure.

### 6.2 Illustration with real data in a sequential two-mediator scenario

#### 6.2.1 Justification of the causal diagram

**Figure 12:**
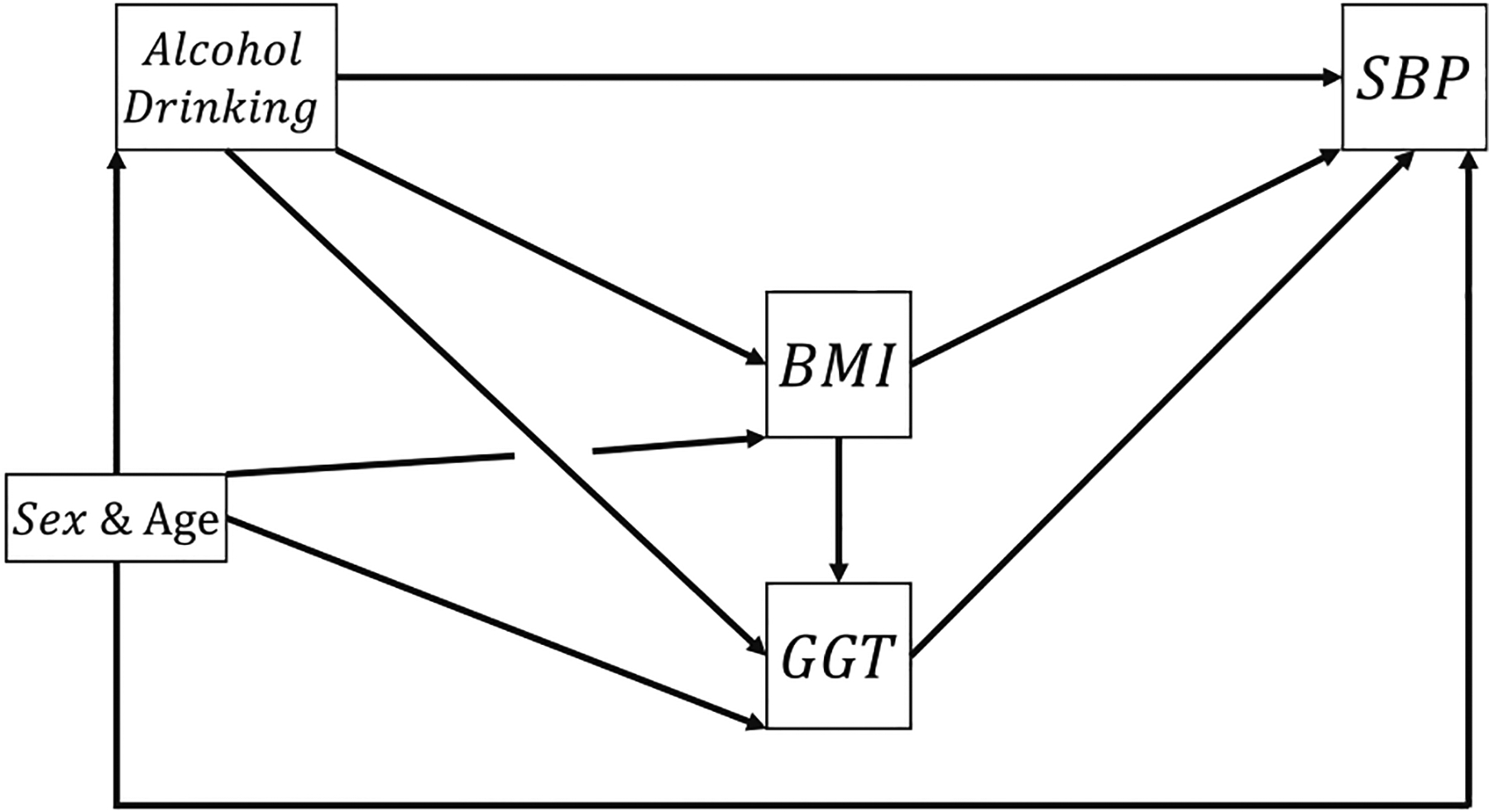
Directed acyclic graph for the study on hazard of drinking alcohol, where alcohol drinking is used as the exposure, BMI and log-transformed GGT as the two sequential mediators, SBP as the outcome, and sex and age as two confounders.

In our motivating example, we aim to examine the effect of alcohol consumption on hypertension, and the components of the TE that are due to the mediation or interaction with GGT and BMI. The hypothetical causal diagram with two sequential mediators is shown in Figure 12. We adopted the causal diagram from the study by Daniel et al. [3], and provided additional evidence from literature reports to support the causal diagram. While GGT is traditionally used as a biological marker for excessive alcohol consumption and liver function [25], it has been suggestive to be a robust marker for oxidative stress [26,27]. There is growing evidence that obesity, especially central obesity, may result in increased serum GGT levels [28,29]. Experimental and clinical studies have demonstrated the important role of GGT in antioxidant defense, detoxification, and inflammation processes [30]. There are a number of reports that have investigated the effects of GGT on the risk and prognosis of complex diseases such as cancer [31] and cardiovascular disease [32]. A study that has conducted a 12-week alcohol relapse prevention trial reported that participant with positive GGT (≥50 IU) had 10 mmHg greater SBP and 9 mmHg greater diastolic blood pressure (DBP) than those with negative GGT [33]. Mechanistic studies investigating the role of increases in GGT activity in predicting hypertension (commonly defined as SBP ≥140 mmHg or DBP ≥90 mmHg) could be due to a connection with the increased level of arterial stiffness [34,35]. We acknowledge that the biological and pathological mechanisms involving the interactions among adiposity, ethanol, and GGT remain less understood. However, several epidemiological and clinical studies have investigated and reported the combined and interactive effects of excessive ethanol consumption and obesity on the biochemical variables. A study based on an analysis of 8,373 adults in the 2005–2008 National Health and Nutrition Examination Survey showed that the co-occurrence of obesity and patterns of alcohol use are significantly associated with elevated serum GGT [36]. Another study reported additive interaction effects between moderate drinking and obesity on serum GGT activities [37]. A longitudinal study investigating the relationship between serum GGT and risk of hypertension stratified by alcohol consumption status and BMI groups has reported a stronger association among current drinkers than that among non-drinkers [38]. In the same study of subgroup analysis by BMI groups, significant association between serum GGT and hypertension was only found among participants above the median of anthropometric measures (e.g., BMI > 26.4) [38]. These studies suggest potential complex two-way or even three-way interaction effects between BMI, alcohol consumption, and GGT on hypertension that warrant further investigation.

To illustrate the concept of natural MI effect and the decomposition methods, we used the 2013–2014, 2015–2016, and 2017–2018 National Health and Nutrition Examination Survey data with 8,920 observations [3,39]. The data set was downloaded from http://www.cdc.gov/nhanes. Exposure *A* is alcohol drinking and treated as a binary random variable (never/moderate or heavy). As suggested by the Dietary Guidelines for Americans from US Department of Agriculture and US Department of Health and Human Services [40], we define heavy alcohol drinking as consuming 3 or more drinks in a day for males, and consuming 2 or more drinks in a day for females. In our causal diagram, the mediator BMI (*M*_1_) is measured in kg/m^2^, the mediator GGT (*M*_2_) is measured in U/L, and the outcome SBP (*Y*) is measured in mmHg. Sex (females or males) and age (measured in years) are considered a sufficient set satisfying the assumptions on confounding.

Log transformation was performed on GGT due to the skewness of the data. The fixed reference levels of *M*_1_ and log(*M*_2_) were chosen to be the estimated means from data, where $m_{1}^{*}=29.21$ and log(*m*_2_)* = 3.09. Three linear models were fit for *Y*, log(*M*_2_), and *M*_1_, which include all possible interactions among the exposure and mediators. The 95% confidence intervals were obtained by using a bootstrap method [24].

Table 9 presents the decomposition of the TE conditional on males and the mean level of age at 45.96. The CDE is 1.1014 (95% CI = 0.4900 to 1.7218); the reference interaction effect between *A* and *M*_1_ is 0.0329 (−0.0277 to 0.0963); the reference interaction effect between *A* and log(*M*_2_) is 0.0745 (−0.0150 to 0.1706); the reference interaction effect between *A*, *M*_1_, and log(*M*_2_) is 0.0025 (−0.1108 to 0.1151); the natural MI effect between *A* and *M*_1_ is −0.0167 (−0.0670 to 0.0305); the natural MI effect between *A* and log(*M*_2_) is 0.1307 (−0.0383 to 0.3023); the natural MI effect between *A*, *M*_1_, and log(*M*_2_) is 0.0003 (−0.0136 to 0.0143); the natural MI effect between *M*_1_ and log(*M*_2_) is−0.0059 (−0.0195 to 0.0050); the PDE is 1.2113 (0.6011 to 1.8326); the PIE through *M*_1_ is 0.2137 (0.0927 to 0.3417); the seminatural indirect effect through log(*M*_2_) is 0.3952 (0.2581 to 0.5470); and the TE is 1.9287 (1.2874 to 2.5807). The results of the decomposition of the TE conditional on females and the mean level of age are shown in Table 10.

Overall, we observed a significant increase in SBP among heavy alcohol drinkers in both males (TE: 1.9287; 95% CI: 1.2874, 2.5807) and females (TE: 1.5960; 95% CI: 0.9731, 2.2246) compared to never/moderate drinkers. Detailed decomposition using our method showed that all three path effects (PDE, ${\text{PIE}}_{M_{1}}$ and ${\text{SNIE}}_{\text{log}\left(M_{2}\right)}$) significantly contribute to the TE. Among the natural MI effect components, we observed that the interaction effects between alcohol drinking and GGT have the highest magnitude in both females and males, although not statistically significant. The natural MI between alcohol drinking and GGT can be interpreted as the expected value of the product of the mediation effect through GGT and the additive interaction effects between heavy drinkers and the GGT levels, while the BMI is fixed at the potential value for never/moderate drinkers. Compared to never/moderate drinkers, heavy drinkers are associated with an average of 0.13 units higher SBP that is due to the MI effects between alcohol drinking and GGT. This suggests that the mediating and interactive mechanisms for alcohol drinking and GGT are likely operating in the same direction, which results in further increased SBP at the average population level in both females and males. We note that there are potential limitations of the real data analysis. First, we assume that the linear structural equation models are correctly specified. A bias would occur if the true relationships were non-linear. Second, observations with missing data were not considered in the analysis. Third, the data analysis is primarily for illustration purpose. Our data analysis may have limited power in detecting statistically significant reference or MI effects. However, it clearly demonstrates how to decompose the TE into different components. Results suggest that the detected significant TE may be driven by the components other than the interaction effects in this population. These results would also provide helpful information on developing targeted prevention strategies for hypertension. Finally, the causal interpretations in this example should be made with discretion because the identification assumptions on unmeasured confounding might be violated.

## 7 Conclusion

In this work, we develop decompositions for scenarios where the two mediators are causally sequential or non-sequential. We propose a unified approach for decomposing the TE into components that are due to mediation only, interaction only, both mediation and interaction, and neither mediation nor interaction within the counterfactual framework. The decomposition was implemented via a new concept called natural MI effect that we proposed to describe the two-way and three-way interactions for both scenarios that extend the two-way MIs in existing literature. To estimate the components of our proposed decompositions, we lay out the identification assumptions. We also derive the formulas when the response is assumed to be continuous with linear structural equation models. We use both simulated and real data sets to illustrate our method.

We believe that our proposed new concept of natural MI effects and the decomposition methods for the causal framework with two sequential or non-sequential mediators provide a powerful tool to decipher the refined path effects while appropriately account for interaction effects among the exposure and mediators. The counterfactual interaction effects evaluate the interaction terms that involve mediators by treating them at the natural levels. There is a gap in existing research of decomposing TE into mediation and interaction effects for the scenario of multiple sequential mediators, and our proposed methods have the potential to fill in the gap. Our future work will include developing decomposition methods for causal structures involving multiple sequential mediators and multiple exposures. We will also investigate the interventional analogue version of this decomposition and the corresponding interpretation of the effects in the future work.

## Supplementary Material

Supplementary Materials

## Figures and Tables

**Table 1:** Decomposition of the TE in a non-sequential two-mediator scenario when *A*, *M*_1_, and *M*_2_ are binary with *a* = 1, a* = 0, $m_{1}^{*}=0$, and $m_{2}^{*}=0$

Effect^a,b^	Definition	Interpretation
CDE(0, 0)	*Y*(1, 0, 0) − *Y*(0, 0, 0)	Due to neither mediation nor interaction
${\text{INT}}_{\text{ref-}AM_{1}}(0,0)$	[*Y*(1, 1, 0) − *Y*(0, 1, 0) − *Y*(1, 0, 0) + *Y*(0, 0, 0)] × *M*_1_(0)	Due to the interaction between *A* and *M*_1_ only
${\text{INT}}_{\text{ref-}AM_{2}}(0,0)$	[*Y*(1, 0, 1) − *Y*(0, 0, 1) − *Y*(1, 0, 0) + *Y*(0, 0, 0)] × *M*_2_(0)	Due to the interaction between *A* and *M*_2_ only
${\text{INT}}_{\text{ref-}AM_{1}M_{2}}(0,0)$	[*Y* (1, 1, 1) − *Y* (0, 1, 1) − *Y* (1, 0, 1) + *Y* (0, 0, 1) − *Y* (1, 1, 0) + *Y* (0, 1, 0) + *Y* (1, 0, 0) − *Y* (0, 0, 0)] × *M*_1_(0) × *M*_1_(0)	Due to the interaction between *A, M*_1_ and *M*_2_ only
${\text{NatINT}}_{AM_{1}}$	$\sum_{m_{2}}\left[Y\left(1,1,m_{2}\right)/\left(M_{2}(0)=m_{2}\right)-Y\left(0,1,m_{2}\right)/\left(M_{2}(0)=m_{2}\right)-Y\left(1,0,m_{2}\right)/\left(M_{2}(0)=m_{2}\right)+Y\left(0,0,m_{2}\right)/\left(M_{2}(0)=m_{2}\right)\right]\times \left[M_{1}(1)-M_{1}(0)\right]$	Due to the mediation through *M*_1_ and the interaction between *A* and *M*_1_ conditional on the potential value of *M*_2_ with the fixed reference level a* = 0
${\text{NatINT}}_{AM_{2}}$	$\sum_{m_{1}}\left[Y\left(1,m_{1},1\right)/\left(M_{1}(0)=m_{1}\right)-Y\left(0,m_{1},1\right)/\left(M_{1}(0)=m_{1}\right)-Y\left(1,m_{1},0\right)/\left(M_{1}(0)=m_{1}\right)+Y\left(0,m_{1},0\right)/\left(M_{1}(0)=m_{1}\right)\right]\times \left[M_{2}(1)-M_{2}(0)\right]$	Due to the mediation through *M*_2_ and the interaction between *A* and *M*_2_ conditional on the potential value of *M*_1_ with the fixed reference level a* = 0
${\text{NatINT}}_{AM_{1}M_{2}}$	[*Y* (1, 1, 1) − *Y* (0, 1, 1) − *Y* (1, 0, 1) + *Y* (0, 0, 1) − *Y* (1, 1, 0) + *Y* (0, 1, 0) + *Y* (1, 0, 0) − *Y* (0, 0, 0)] × [*M*_1_(1) − *M*_1_(0)] × [*M*_2_(1) − *M*_2_(0)]	Due to the mediation through both *M*_1_ and *M*_2_ and the interaction between *A*, *M*_1_ and *M*_2_
${\text{NatINT}}_{M_{1}M_{2}}$	[*Y* (0, 1, 1) − *Y* (0, 0, 1) − *Y* (0, 1, 0) + *Y* (0, 0, 0)] × [*M*_1_(1) − *M*_1_(0)] × [*M*_2_(1) − *M*_2_(0)]	Due to the mediation through both *M*_1_ and *M*_2_ only
${\text{PIE}}_{M_{1}}$	$\sum_{m_{2}}\left[Y\left(0,1,m_{2}\right)/\left(M_{2}(0)=m_{2}\right)-Y\left(0,0,m_{2}\right)/\left(M_{2}(0)=m_{2}\right)\right]\times \left[M_{1}(1)-M_{1}(0)\right]$	Due to the mediation through *M*_1_ only conditional on the potential value of *M*_2_ with the fixed reference level a* = 0
${\text{PIE}}_{M_{2}}$	$\sum_{m_{1}}\left[Y\left(0,m_{1},1\right)/\left(M_{1}(0)=m_{1}\right)-Y\left(0,m_{1},0\right)/\left(M_{1}(0)=m_{1}\right)\right]\times \left[M_{2}(1)-M_{2}(0)\right]$	Due to the mediation through *M*_2_ only conditional on the potential value of *M*_1_ with the fixed reference level a* = 0

a The CDE and reference interaction effects are the same as those proposed by Bellavia and Valeri [9].

b CDE denotes controlled direct effect; INT_ref_ denotes reference interaction effect; NatINT denotes natural MI effect; PIE denotes PIE.

**Table 2:** Proposed mediated effects in a non-sequential two-mediator scenario with binary *A*, *M*_1_, and *M*_2_ under the Assumption *M*_1_(0) = *M*_2_(0) = 0

Effect^a^	Definition	Interpretation
${\text{NatINT}}_{AM_{1}}$	[*Y* (1, 1, 0) − *Y* (0, 1, 0) − *Y* (1, 0, 0) + *Y* (0, 0, 0)] × [*M*_1_(1) − *M*_1_(0)]	Due to the mediation through *M*_1_ and the interaction between *A* and *M*_1_ assuming *M*_2_(0) = 0
${\text{NatINT}}_{AM_{2}}$	[*Y* (1, 0, 1) − *Y* (0, 0, 1) − *Y* (1, 0, 0) + *Y* (0, 0, 0)] × [*M*_2_(1) − *M*_2_(0)]	Due to the mediation through *M*_2_ and the interaction between *A* and *M*_2_ assuming *M*_1_(0) = 0
${\text{NatINT}}_{AM_{1}M_{2}}$	[*Y*(1, 1, 1) − *Y*(0, 1, 1) − *Y*(1, 0, 1) + *Y*(0, 0, 1) − *Y*(1, 1, 0) + *Y*(0, 1, 0) + *Y*(1, 0, 0) − *Y*(0, 0, 0)] × [*M*_1_(1)*M*_2_(1) − *M*_1_(0)*M*_2_(0)]	Due to the mediation through both *M*_1_ and *M*_2_ and the interaction between *A*, *M*_1_ and *M*_2_ assuming *M*_1_(0) = *M*_2_(0) = 0
${\text{NatINT}}_{M_{1}M_{2}}$	[*Y*(0, 1, 1) − *Y*(0, 0, 1) − *Y*(0, 1, 0) + *Y*(0, 0, 0)] × [*M*_1_(1)*M*_2_(1) − *M*_1_(0)*M*_2_(0)]	Due to the mediation through both *M*_1_ and *M*_2_ only assuming *M*_1_(0) = *M*_2_(0) = 0
${\text{PIE}}_{M_{1}}$	[*Y*(0, 1, 0) − *Y*(0, 0, 0)] × [*M*_1_(1) − *M*_1_(0)]	Due to the mediation through *M*_1_ only assuming *M*_2_(0) = 0
${\text{PIE}}_{M_{2}}$	[*Y*(0, 0, 1) − *Y*(0, 0, 0)] × [*M*_2_(1) − *M*_2_(0)]	Due to the mediation through *M*_2_ only assuming *M*_1_(0) = 0

a NatINT denotes natural MI effect; PIE denotes pure indirect effect.

**Table 3:** Decomposition of the TE in a sequential two-mediator scenario when *A*, *M*_1_, and *M*_2_ are binary with *a* = 1, a* = 0, $m_{1}^{*}=0$, and $m_{2}^{*}=0$

Effect^a^	Definition	Interpretation
CDE(0, 0)	*Y*(1, 0, 0) − *Y*(0, 0, 0)	Due to neither mediation nor interaction
${\text{INT}}_{\text{ref-}AM_{1}}(0,0)$	[*Y*(1, 1, 0) − *Y*(0, 1, 0) - *Y*(1, 0, 0) + *Y*(0, 0, 0)] × *M*_1_(0)	Due to the interaction between *A* and *M*_1_ only
${\text{INT}}_{\text{ref-}AM_{2}}(0,0)$	[*Y*(1, 0, 1) − *Y*(0, 0, 1) − *Y*(1, 0, 0) + *Y*(0, 0, 0)] × *M*_2_(0, 0)	Due to the interaction between *A* and *M*_2_ only
${\text{INT}}_{\text{ref-}AM_{1}M_{2}}(0,0)$	[*Y*(1, 1, 1) − *Y*(0, 1, 1) − *Y*(1, 1, 0) + *Y*(0, 1, 0)] × *M*_1_(0) × *M*_2_(0, 1) + [−*Y*(1, 0, 1) + *Y*(0, 0, 1) + *Y*(1, 0, 0) − *Y*(0, 0, 0)] × *M*_1_(0) × *M*_2_(0, 0)	Due to the interaction between *A, M*_1_ and *M*_2_ only
${\text{NatINT}}_{AM_{1}}$	$\sum_{m_{2}}\left[Y\left(1,1,m_{2}\right)/\left(M_{2}(0,1)=m_{2}\right)-Y\left(0,1,m_{2}\right)/\left(M_{2}(0,1)=m_{2}\right)-Y\left(1,0,m_{2}\right)/\left(M_{2}(0,0)=m_{2}\right)+Y\left(0,0,m_{2}\right)/\left(M_{2}(0,0)=m_{2}\right)\right]\times \left[M_{1}(1)-M_{1}(0)\right]$	Due to the mediation through *M*_1_ and the interaction between *A* and *M*_1_ conditional on the potential values of *M*_2_ with the fixed reference level a* = 0
${\text{NatINT}}_{AM_{2}}$	$\sum_{m_{1}}\left[Y\left(1,m_{1},1\right)/\left(M_{1}(0)=m_{1}\right)-Y\left(0,m_{1},1\right)/\left(M_{1}(0)=m_{1}\right)-Y\left(1,m_{1},0\right)/\left(M_{1}(0)=m_{1}\right)+Y\left(0,m_{1},0\right)/\left(M_{1}(0)=m_{1}\right)\right]\times \left[M_{2}\left(1,m_{1}\right)-M_{2}\left(0,m_{1}\right)\right]$	Due to the mediation through *M*_2_ and the interaction between *A* and *M*_2_ conditional on the potential value of *M*_1_ with the fixed reference level a* = 0
${\text{NatINT}}_{AM_{1}M_{2}}$	[*Y*(1, 1, 1) − *Y*(0, 1, 1) − *Y*(1, 1, 0) + *Y*(0, 1, 0)] × [*M*_1_(1) − *M*_1_(0)] × [*M*_2_(1, 1) − *M*_2_(0, 1)] + [−*Y*(1, 0, 1) + *Y*(0, 0, 1) + *Y*(1, 0, 0) − *Y*(0, 0, 0)] × [*M*_1_(1) − *M*_1_(0)] × [M_2_(1, 0) − *M*_2_(0, 0)]	Due to the mediation through both *M*_1_ and *M*_2_ and the interaction between *A, M*_1_ and *M*_2_
${\text{NatINT}}_{M_{1}M_{2}}$	[*Y*(0, 1, 1) − *Y*(0, 1, 0)] × [*M*_1_(1) − *M*_1_(0)] × [*M*_2_(1, 1) − *M*_2_(0, 1)] + [−*Y*(0, 0, 1) + *Y*(0, 0, 0)] × [*M*_1_(1) − *M*_1_(0)] × [*M*_2_(1, 0) − *M*_2_(0, 0)]	Due to the mediation through both *M*_1_ and *M*_2_ only
${\text{PIE}}_{M_{1}}$	$\sum_{m_{2}}\left[Y\left(0,1,m_{2}\right)/\left(M_{2}(0,1)=m_{2}\right)-Y\left(0,0,m_{2}\right)/\left(M_{2}(0,0)=m_{2}\right)\right]\times \left[M_{1}(1)-M_{1}(0)\right]$	Due to the mediation through *M*_1_ only conditional on the potential values of *M*_2_ with the fixed reference level a* = 0
${\text{SNIE}}_{M_{2}}$	$\sum_{m_{1}}\left[Y\left(0,m_{1},1\right)\times I\left(M_{1}(0)=m_{1}\right)-Y\left(0,m_{1},0\right)\times I\left(M_{1}(0)=m_{1}\right)\right]\times \left[M_{2}\left(1,m_{1}\right)-M_{2}\left(0,m_{1}\right)\right]$	Due to the partial mediation through *M*_2_ only conditional on the potential value of *M*_1_ with the fixed reference level a* = 0

a CDE denotes controlled direct effect; INT_ref_ denotes reference interaction effect; NatINT denotes natural MI effect; PIE denotes pure indirect effect.

**Table 4:** Suggested interaction decompositions for both a non-sequential and a sequential two-mediator scenario^a^

Number of components	Decomposition^b^
2-Way decomposition (no mediation)	$\text{CDE}\left(m_{1}^{*},m_{2}^{*}\right)+\text{PAI}$
4-Way decomposition	$\text{CDE}\left(m_{1}^{*},m_{2}^{*}\right)+\text{PAI}+{\text{PIE}}_{M_{1}}+{\text{PIE}}_{M_{2}}\left({\text{or SNIE}}_{M_{2}}\right)$
4-Way decomposition	$\text{TDE}+{\text{NatINT}}_{M_{1}M_{2}}+{\text{PIE}}_{M_{1}}+{\text{PIE}}_{M_{2}}\left({\text{or SNIE}}_{M_{2}}\right)$
5-Way decomposition	$\text{CDE}\left(m_{1}^{*},m_{2}^{*}\right)+{\text{INT}}_{\text{ref-}AM_{1}}\left(m_{1}^{*},m_{2}^{*}\right)+{\text{INT}}_{\text{ref-}AM_{2}}\left(m_{1}^{*},m_{2}^{*}\right)+{\text{INT}}_{\text{ref-}AM_{1}M_{2}}\left(m_{1}^{*},m_{2}^{*}\right)+\text{TIE}$
7-Way decomposition	$\text{PDE}+{\text{NatINT}}_{AM_{1}}+{\text{NatINT}}_{AM_{\text{2}}}+{\text{NatINT}}_{AM_{1}M_{2}}+{\text{NatINT}}_{M_{1}M_{2}}+{\text{PIE}}_{M_{1}}+{\text{PIE}}_{M_{2}}\left({\text{or SNIE}}_{M_{2}}\right)$
10-Way decomposition	$\text{CDE}\left(m_{1}^{*},m_{2}^{*}\right)+{\text{INT}}_{\text{ref-}AM_{1}}\left(m_{1}^{*},m_{2}^{*}\right)+{\text{INT}}_{\text{ref-}AM_{2}}\left(m_{1}^{*},m_{2}^{*}\right)+{\text{INT}}_{\text{ref-}AM_{1}M_{2}}\left(m_{1}^{*},m_{2}^{*}\right)+{\text{NatINT}}_{AM_{1}}+{\text{NatINT}}_{AM_{2}}+{\text{NatINT}}_{AM_{1}M_{2}}+{\text{ NatINT}}_{M_{1}M_{2}}+{\text{PIE}}_{M_{1}}+{\text{PIE}}_{M_{2}}{\text{(or SNIE}}_{M_{2}}\text{) }$

a Use ${\text{SNIE}}_{M_{2}}$ instead of ${\text{PIE}}_{M_{2}}$ in a sequential two-mediator scenario.

b CDE denotes controlled direct effect; INT_ref_ denotes reference interaction effect; NatINT denotes natural MI effect; PIE denotes pure indirect effect; PAI denotes portion attributable to interaction; SNIE denotes seminatural indirect effect; TDE denotes total direct effect; TIE denotes total indirect effect; PDE denotes pure direct effect.

**Table 5:** Comparison of the mediated effects between Bellavia’s and Valeri’s method^a^ and our proposed decomposition^b^ in the formulas^c^ under linear structural equation models in a non-sequential two-mediator scenario

Bellavia’s and Valeri’s method		Our proposed decomposition	
Component^d,e^	Formula	Component^f^	Formula
$E\left[{\text{INTmed}}_{AM_{1}}|m_{2}^{*},c\right]$	$\left(\theta_{4}+\theta_{7}m_{2}^{*}\right)\gamma_{1}{\left(a-a^{*}\right)}^{2}$	$E\left[{\text{NatINT}}_{AM_{1}}|c\right]$	$\left[\theta_{4}+\theta_{7}\left(\beta_{0}+\beta_{1}a^{*}+\beta_{4}^{\prime }c\right)\right]\gamma_{1}{\left(a-a^{*}\right)}^{2}$
$E\left[{\text{INTmed}}_{AM_{2}}|m_{1}^{*},c\right]$	$\left(\theta_{5}+\theta_{7}m_{1}^{*}\right)\beta_{1}{\left(a-a^{*}\right)}^{2}$	$E\left[{\text{NatINT}}_{AM_{2}}|c\right]$	$\left[\theta_{5}+\theta_{7}\left(\gamma_{0}+\gamma_{1}a^{*}+\gamma_{2}^{\prime }c\right)\right]\beta_{1}{\left(a-a^{*}\right)}^{2}$
$E\left[{\text{INTmed}}_{AM_{1}M_{2}}|m_{1}^{*},m_{2}^{*},c\right]$	$\left[\beta_{1}\left(\gamma_{0}+\gamma_{2}^{\prime }c-m_{1}^{*}\right)+\gamma_{1}\left(\beta_{0}+\beta_{4}^{\prime }c-m_{2}^{*}\right)+\beta_{1}\gamma_{1}\left(a+a^{*}\right)\right]\theta_{7}{\left(a-a^{*}\right)}^{2}$	$E\left[{\text{NatINT}}_{AM_{1}M_{2}}|c\right]$	$\theta_{7}\beta_{1}\gamma_{1}{\left(a-a^{*}\right)}^{3}$
$E\left[{\text{PNIE}}_{M_{1}M_{2}}|m_{1}^{*},m_{2}^{*},c\right]$	$\left[\gamma_{1}\left(\beta_{0}+\beta_{4}^{\prime }c\right)+\beta_{1}\left(\gamma_{0}+\gamma_{2}^{\prime }c\right)-\gamma_{1}m_{2}^{*}-\beta_{1}m_{1}^{*}+\gamma_{1}\beta_{1}\left(a+a^{*}\right)\right]\times \left(\theta_{6}+\theta_{7}a^{*}\right)\left(a-a^{*}\right)$	$E\left[{\text{NatINT}}_{M_{1}M_{2}}|c\right]$	$\beta_{1}\gamma_{1}\left(\theta_{6}+\theta_{7}a^{*}\right){\left(a-a^{*}\right)}^{2}$
$E\left[{\text{PNIE}}_{M_{1}}|m_{2}^{*},c\right]$	$\left[\theta_{2}+\theta_{4}a^{*}+\left(\theta_{6}+\theta_{7}a^{*}\right)m_{2}^{*}\right]\gamma_{1}\left(a-a^{*}\right)$	$E\left[{\text{PIE}}_{M_{1}}|c\right]$	$\left[\theta_{2}+\theta_{4}a^{*}+\left(\theta_{6}+\theta_{7}a^{*}\right)\left(\beta_{0}+\beta_{1}a^{*}+\beta_{4}^{\prime }c\right)\right]\gamma_{1}\left(a-a^{*}\right)$
$E\left[{\text{PNIE}}_{M_{2}}|m_{1}^{*},c\right]$	$\left[\theta_{3}+\theta_{5}a^{*}+\left(\theta_{6}+\theta_{7}a^{*}\right)m_{1}^{*}\right]\beta_{1}\left(a-a^{*}\right)$	$E\left[{\text{PIE}}_{M_{2}}|c\right]$	$\left[\theta_{3}+\theta_{5}a^{*}+\left(\theta_{6}+\theta_{7}a^{*}\right)\left(\gamma_{0}+\gamma_{1}a^{*}+\gamma_{2}^{\prime }c\right)\right]\beta_{1}\left(a-a^{*}\right)$

a The formulas in Bellavia’s and Valeri’s method are derived according to Web Table 2 in the study by Bellavia and Valeri [9].

b The formulas in our proposed decomposition are obtained by setting *β*_2_ and *β*_3_ to 0 in a sequential two-mediator scenario.

c All formulas under linear structural equation models are based on a continuous outcome *Y* and two continuous non-sequential mediators *M*_1_ and *M*_2_. The structural equation models are as follows:

$$E\left[Y|A,M_{1},M_{2},C\right]=\theta_{0}+\theta_{1}A+\theta_{2}M_{1}+\theta_{3}M_{2}+\theta_{4}AM_{1}+\theta_{5}AM_{2}+\theta_{6}M_{1}M_{2}+\theta_{7}AM_{1}M_{2}+\theta_{8}^{\prime }C,$$


$$E\left[M_{2}|A,C\right]=\beta_{0}+\beta_{1}A+\beta_{4}^{\prime }C,$$


$$E\left[M_{1}|A,C\right]=\gamma_{0}+\gamma_{1}A+\gamma_{2}^{\prime }C.$$

d The components in Bellavia’s and Valeri’s method are conditional on $M_{1}\left(a^{*}\right)=m_{1}^{*}$ and/or $M_{2}\left(a^{*}\right)=m_{2}^{*}$. Only $m_{1}^{*}$ and/or $m_{2}^{*}$ are shown in Table 5 for simplicity.

e INTmed denotes MI effect; PNIE denotes pure NIE.

f NatINT denotes natural MI effect; PIE denotes pure indirect effect.

**Table 6:** Simulation results^a^ and corresponding interpretations^b^ of identical components in Bellavia’s and Valeri’s method and our proposed decomposition

Component^c^	True value	Estimate	95% CI	Interpretation
CDE(0, 0)	0.3000	0.2891	0.2210, 0.3590	Due to neither mediation nor interaction with fixed reference levels $m_{1}^{*}=m_{2}^{*}=0$
${\text{INT}}_{\text{ref-}AM_{1}}(0,0)$	0.0024	0.0003	−0.0082, 0.0088	Due to the interaction between *A* and *M*_1_only with fixed reference levels $m_{1}^{*}=m_{2}^{*}=0$
${\text{INT}}_{\text{ref-}AM_{2}}(0,0)$	0.0048	0.0101	0.0029, 0.0181	Due to the interaction between *A* and *M*_2_ only with fixed reference levels $m_{1}^{*}=m_{2}^{*}=0$
${\text{INT}}_{\text{ref-}AM_{1}M_{2}}(0,0)$	0.0403	0.0332	0.0212, 0.0470	Due to the interaction between *A, M*_1_ and *M*_2_ only with fixed reference levels $m_{1}^{*}=m_{2}^{*}=0$
PDE	0.3475	0.3327	0.2647, 0.4012	The causal effect through the direct path *A* → *Y*
TE	0.8707	0.8697	0.7841, 0.9561	The overall causal effect of *A* on *Y*

a The simulation results are calculated from the following structural equation models:

$$E\left[Y|A,M_{1},M_{2},C\right]=0.2+0.3A+0.3M_{1}+0.4M_{2}+0.01AM_{1}+0.02AM_{2}+0.6M_{1}M_{2}+0.7AM_{1}M_{2}+0.2C,$$


$$E\left[M_{2}|A,C\right]=0.2+0.3A+0.2C,$$


$$E\left[M_{1}|A,C\right]=0.2+0.3A+0.2C.$$

b All effects are calculated from the contrast between *a* = 1 and a* = 0.

c CDE denotes controlled direct effect; INT_ref_ denotes reference interaction effect; PDE denotes pure direct effect; TE denotes TE.

**Table 7:** Simulation results^a^ of different components in Bellavia’s and Valeri’s method and our proposed decomposition

Bellavia’s and Valeri’s method				Our proposed decomposition			
Component^b^	True value	Estimate	95% CI	Component^c^	True value	Estimate	95% CI
${\text{INTmed}}_{AM_{1}}$	0.0030	0.0004	−0.0107, 0.0116	${\text{NatINT}}_{AM_{1}}$	0.0534	0.0439	0.0260, 0.0634
${\text{INTmed}}_{AM_{2}}$	0.0060	0.0165	0.0048, 0.0283	${\text{NatINT}}_{AM_{2}}$	0.0564	0.0703	0.0499, 0.0922
${\text{INTmed}}_{AM_{1}M_{2}}$	0.1638	0.1680	0.1474, 0.1887	${\text{NatINT}}_{AM_{1}M_{2}}$	0.0630	0.0706	0.0521, 0.0911
${\text{PNIE}}_{M_{1}M_{2}}$	0.1404	0.1286	0.1021, 0.1573	${\text{NatINT}}_{M_{1}M_{2}}$	0.0540	0.0541	0.0378, 0.0734
${\text{PNIE}}_{M_{1}}$	0.0900	0.0902	0.0626, 0.1207	${\text{PIE}}_{M_{1}}$	0.1332	0.1236	0.0919, 0.1579
${\text{PNIE}}_{M_{2}}$	0.1200	0.1333	0.1011, 0.1688	${\text{PIE}}_{M_{2}}$	0.1632	0.1745	0.1353, 0.2174

a The simulation results are calculated from the following structural equation models:

$$E\left[Y|A,M_{1},M_{2},C\right]=0.2+0.3A+0.3M_{1}+0.4M_{2}+0.01AM_{1}+0.02AM_{2}+0.6M_{1}M_{2}+0.7AM_{1}M_{2}+0.2C,$$


$$E\left[M_{2}|A,C\right]=0.2+0.3A+0.2C,$$


$$E\left[M_{1}|A,C\right]=0.2+0.3A+0.2C.$$

b INTmed denotes MI effect; PNIE denotes pure NIE.

c NatINT denotes natural MI effect; PIE denotes pure indirect effect.

**Table 8:** Corresponding interpretations^a^ for the simulation results of different components in Bellavia’s and Valeri’s method and our proposed decomposition

Bellavia’s and Valeri’s method		Our proposed decomposition	
Component^b^	Interpretation	Component^c^	Interpretation
${\text{INTmed}}_{AM_{1}}$	Due to the mediation through *M*_1_ and the interaction between *A* and *M*_1_ assuming *M*_2_(0) = 0	${\text{NatINT}}_{AM_{1}}$	Due to the mediation through *M*_1_ and the interaction between *A* and *M*_1_ with *M*_2_(0) estimated from data
${\text{INTmed}}_{AM_{2}}$	Due to the mediation through *M*_2_ and the interaction between *A* and *M*_2_ assuming *M*_1_(0) = 0	${\text{NatINT}}_{AM_{2}}$	Due to the mediation through *M*_2_ and the interaction between *A* and *M*_2_ with *M*_1_(0) estimated from data
${\text{INTmed}}_{AM_{1}M_{2}}$	Due to the mediation through both *M*_1_ and *M*_2_ and the interaction between *A*, *M*_1_, and *M*_2_ assuming *M*_1_(0) = *M*_2_(0) = 0	${\text{NatINT}}_{AM_{1}M_{2}}$	Due to the mediation through both *M*_1_ and *M*_2_ and the interaction between *A*, *M*_1_, and *M*_2_ with *M*_1_(0) and *M*_2_(0) estimated from data
${\text{PNIE}}_{M_{1}M_{2}}$	Due to the mediation through both *M*_1_ and *M*_2_ only assuming *M*_1_(0) = *M*_2_(0) = 0	${\text{NatINT}}_{M_{1}M_{2}}$	Due to the mediation through both *M*_1_ and *M*_2_ only with *M*_1_(0) and *M*_2_(0) estimated from data
${\text{PNIE}}_{M_{1}}$	Due to the mediation through *M*_1_ only assuming *M*_2_(0) = 0	${\text{PIE}}_{M_{1}}$	Due to the mediation through *M*_1_ only with *M*_2_(0) estimated from data
${\text{PNIE}}_{M_{2}}$	Due to the mediation through *M*_2_ only assuming *M*_1_(0) = 0	${\text{PIE}}_{M_{2}}$	Due to the mediation through *M*_2_ only with *M*_1_(0) estimated from data

a All effects are calculated from the contrast between *a* = 1 and a* = 0.

b INTmed denotes MI effect; PNIE denotes pure NIE.

c NatINT denotes natural MI effect; PIE denotes pure indirect effect.

**Table 9:** Illustration with real data: decomposition of TE conditional on males and the mean age^a^

Component^b^	Estimate	95% CI
$\text{CDE}\left(m_{1}^{*},\text{log}{\left(m_{2}\right)}^{*}\right)$	1.1014	0.4900, 1.7218
${\text{INT}}_{\text{ref-}AM_{1}}\left(m_{1}^{*},\text{log}{\left(m_{2}\right)}^{*}\right)$	0.0329	−0.0277, 0.0963
${\text{INT}}_{\text{ref-}A\text{ log}\left(M_{2}\right)}\left(m_{1}^{*},\text{log}{\left(m_{2}\right)}^{*}\right)$	0.0745	−0.0150, 0.1706
${\text{INT}}_{\text{ref-}AM_{1}\text{ log}\left(M_{2}\right)}\left(m_{1}^{*},\text{log}{\left(m_{2}\right)}^{*}\right)$	0.0025	−0.1108, 0.1151
${\text{NatINT}}_{AM_{1}}$	−0.0167	−0.0670, 0.0305
${\text{NatINT}}_{A\text{ log}\left(M_{2}\right)}$	0.1307	−0.0383, 0.3023
${\text{NatINT}}_{AM_{1}\text{ log}\left(M_{2}\right)}$	0.0003	−0.0136, 0.0143
${\text{NatINT}}_{M_{1}\text{ log}\left(M_{2}\right)}$	−0.0059	−0.0195, 0.0050
PDE	1.2113	0.6011, 1.8326
${\text{PIE}}_{M_{1}}$	0.2137	0.0927, 0.3417
${\text{SNIE}}_{\text{log}\left(M_{2}\right)}$	0.3952	0.2581, 0.5470
TE	1.9287	1.2874, 2.5807

a The exposure *A* is alcohol drinking; the mediator *M*_1_ is BMI; the mediator *M*_2_ is GGT; the outcome *Y* is SBP; the confounding covariate set contains sex and age.

b CDE denotes controlled direct effect; INT_ref_ denotes reference interaction effect; NatINT denotes natural MI effect; PDE denotes pure direct effect; PIE denotes pure indirect effect; SNIE denotes seminatural indirect effect; TE denotes total effect.

**Table 10:** Illustration with real data: decomposition of TE conditional on females and the mean age^a^

Component^b^	Estimate	95% CI
$\text{CDE}\left(m_{1}^{*},\text{log}{\left(m_{2}\right)}^{*}\right)$	1.1014	0.4900, 1.7218
${\text{INT}}_{\text{ref-}AM_{1}}\left(m_{1}^{*},\text{log}{\left(m_{2}\right)}^{*}\right)$	−0.0097	−0.0426, 0.0093
${\text{INT}}_{\text{ref-}A\text{ log}\left(M_{2}\right)}\left(m_{1}^{*},\text{log}{\left(m_{2}\right)}^{*}\right)$	−0.2218	−0.4945, 0.0458
${\text{INT}}_{\text{ref-}AM_{1}\text{ log}\left(M_{2}\right)}\left(m_{1}^{*},\text{log}{\left(m_{2}\right)}^{*}\right)$	0.0153	−0.0971, 0.1270
${\text{NatINT}}_{AM_{1}}$	−0.0195	−0.0719, 0.0290
${\text{NatINT}}_{A\text{ log}\left(M_{2}\right)}$	0.1312	−0.0310, 0.2968
${\text{NatINT}}_{AM_{1}\text{ log}\left(M_{2}\right)}$	0.0003	−0.0132, 0.0139
${\text{NatINT}}_{M_{1}\text{ log}\left(M_{2}\right)}$	−0.0058	−0.0190, 0.0049
PDE	0.8853	0.2567, 1.5150
${\text{PIE}}_{M_{1}}$	0.2193	0.0949, 0.3512
${\text{SNIE}}_{\text{log}\left(M_{2}\right)}$	0.3852	0.2527, 0.5319
TE	1.5960	0.9731, 2.2246

a The exposure *A* is alcohol drinking; the mediator *M*_1_ is BMI; the mediator *M*_2_ is GGT; the outcome *Y* is SBP; the confounding covariate set contains sex and age.

b CDE denotes controlled direct effect; INT_ref_ denotes reference interaction effect; NatINT denotes natural MI effect; PDE denotes pure direct effect; PIE denotes pure indirect effect; SNIE denotes seminatural indirect effect; TE denotes total effect.

## Data Availability

The R scripts for the simulation study and real data analysis are available at: https://github.com/flourish-727/data_analysis.
