# Supplementary material for: Decomposition of the total effect for two mediators: A natural mediated interaction effect framework
Source: J Causal Inference. Author manuscript; Available in PMC 2022 May 27. (PMC9139468; doi:10.1515/jci-2020-0017)
Supplement: Supplementary Materials [file NIHMS1808233-supplement-Supplementary_Materials.pdf]

Supplementary material

S1 Decomposition of total effect with the notion of natural MI effect in a non-sequential two-mediator scenario and the corresponding interpretations

Suppose we have a directed acyclic graph as shown in Figure 3. We show in the following that the TE can be decomposed into the following ten components at the individual level:

$$\begin{aligned}\text{TE} = {} & \text{CDE}(m_1^*, m_2^*) + \text{INT}_{\text{ref-AM}_1}(m_1^*, m_2^*) + \text{INT}_{\text{ref-AM}_2}(m_1^*, m_2^*) + \text{INT}_{\text{ref-AM}_1M_2}(m_1^*, m_2^*)\\ & + \text{NatINT}_{AM_1} + \text{NatINT}_{AM_2} + \text{NatINT}_{AM_1M_2} + \text{NatINT}_{M_1M_2} + \text{PIE}_{M_1} + \text{PIE}_{M_2},\end{aligned}$$

where the natural MI effects are listed in Definition 2. We also give the corresponding interpretation for each component.

Proof. We first decompose the TE into TDE [16], NIE through M_1 (NIE_{M_1}) [3] and PIE (path-specific effect) through M_2 (PIE_{M_2}) [16,17].

$$\begin{aligned}\text{TE} &= Y(a) - Y(a^*)\\ &= Y(a, M_1(a), M_2(a)) - Y(a^*, M_1(a^*), M_2(a^*))\\ &= Y(a, M_1(a), M_2(a)) - Y(a^*, M_1(a), M_2(a)) + Y(a^*, M_1(a), M_2(a)) - Y(a^*, M_1(a^*), M_2(a))\\ &\quad + Y(a^*, M_1(a^*), M_2(a)) - Y(a^*, M_1(a^*), M_2(a^*)),\end{aligned}$$

where the second equality follows the composition axiom [8,15] and the third equality follows by adding and subtracting the same counterfactual formulas.

The formulas of TDE, NIE_{M_1}, and PIE_{M_2} are presented as follows:

$$\begin{aligned}\text{TDE} &= Y(a, M_1(a), M_2(a)) - Y(a^*, M_1(a), M_2(a)),\\ \text{NIE}_{M_1} &= Y(a^*, M_1(a), M_2(a)) - Y(a^*, M_1(a^*), M_2(a)),\\ \text{PIE}_{M_2} &= Y(a^*, M_1(a^*), M_2(a)) - Y(a^*, M_1(a^*), M_2(a^*)),\end{aligned}$$

where $\text{TE} = \text{TDE} + \text{NIE}_{M_1} + \text{PIE}_{M_2}$.

We focus on TDE in the next step and decompose it into natural MI effects and PDE [16] by subtracting PDE from TDE, where PDE satisfies the definition of a path-specific effect [17] and equals the following contrast of two counterfactual formulas:

$$\text{PDE} = Y(a, M_1(a^*), M_2(a^*)) - Y(a^*, M_1(a^*), M_2(a^*)).$$

We have the following results:

$$\begin{aligned}\text{TDE-PDE} &= Y(a, M_1(a), M_2(a)) - Y(a^*, M_1(a), M_2(a)) - Y(a, M_1(a^*), M_2(a^*)) + Y(a^*, M_1(a^*), M_2(a^*))\\ &= Y(a, M_1(a), M_2(a)) - Y(a^*, M_1(a), M_2(a)) - Y(a, M_1(a^*), M_2(a^*)) + Y(a^*, M_1(a^*), M_2(a^*))\\ &\quad + Y(a^*, M_1(a^*), M_2(a^*)) - Y(a^*, M_1(a^*), M_2(a^*)) + Y(a^*, M_1(a^*), M_2(a)) - Y(a^*, M_1(a^*), M_2(a))\\ &\quad + Y(a^*, M_1(a), M_2(a^*)) - Y(a^*, M_1(a), M_2(a^*)) + Y(a, M_1(a^*), M_2(a^*)) - Y(a, M_1(a^*), M_2(a^*))\\ &\quad + Y(a, M_1(a^*), M_2(a)) - Y(a, M_1(a^*), M_2(a)) + Y(a, M_1(a), M_2(a^*)) - Y(a, M_1(a), M_2(a^*))\\ &= Y(a, M_1(a), M_2(a^*)) - Y(a^*, M_1(a), M_2(a^*)) - Y(a, M_1(a^*), M_2(a^*)) + Y(a^*, M_1(a^*), M_2(a^*))\\ &\quad + Y(a, M_1(a^*), M_2(a)) - Y(a^*, M_1(a^*), M_2(a)) - Y(a, M_1(a^*), M_2(a^*)) + Y(a^*, M_1(a^*), M_2(a^*))\\ &\quad + Y(a, M_1(a), M_2(a)) - Y(a^*, M_1(a), M_2(a)) - Y(a, M_1(a^*), M_2(a)) + Y(a^*, M_1(a^*), M_2(a))\\ &\quad - Y(a, M_1(a), M_2(a^*)) + Y(a^*, M_1(a), M_2(a^*)) + Y(a, M_1(a^*), M_2(a^*)) - Y(a^*, M_1(a^*), M_2(a^*)),\end{aligned}$$

where the second equality follows by adding and subtracting the same counterfactual formulas, and the third equality follows by rearranging all the terms.

Therefore, we have the following formulas satisfying Definition 2:

$$\begin{aligned}
\text{NatINT}_{AM_1} &= Y(a, M_1(a), M_2(a^*)) - Y(a^*, M_1(a), M_2(a^*)) - Y(a, M_1(a^*), M_2(a^*)) + Y(a^*, M_1(a^*), M_2(a^*)),\\
\text{NatINT}_{AM_2} &= Y(a, M_1(a^*), M_2(a)) - Y(a^*, M_1(a^*), M_2(a)) - Y(a, M_1(a^*), M_2(a^*)) + Y(a^*, M_1(a^*), M_2(a^*)),\\
\text{NatINT}_{AM_1M_2} &= Y(a, M_1(a), M_2(a)) - Y(a^*, M_1(a), M_2(a)) - Y(a, M_1(a^*), M_2(a)) + Y(a^*, M_1(a^*), M_2(a)),\\
&\quad - Y(a, M_1(a), M_2(a^*)) + Y(a^*, M_1(a), M_2(a^*)) + Y(a, M_1(a^*), M_2(a^*)) - Y(a^*, M_1(a^*), M_2(a^*)).
\end{aligned}$$

Accordingly, TDE can be decomposed into the following components:

$$\text{TDE} = \text{PDE} + \text{NatINT}_{AM_1} + \text{NatINT}_{AM_2} + \text{NatINT}_{AM_1M_2}.$$

We next focus on PDE (path-specific effect) and decompose it into CDE and reference interaction effects [7,9]:

$$\begin{aligned}
\text{PDE} &= Y(a, M_1(a^*), M_2(a^*)) - Y(a^*, M_1(a^*), M_2(a^*))\\
&= \sum_{m_2}\sum_{m_1} Y(a, m_1, m_2) \times I(M_1(a^*) = m_1) \times I(M_2(a^*) = m_2) - \sum_{m_2}\sum_{m_1} Y(a^*, m_1, m_2) \times I(M_1(a^*) = m_1)\\
&\quad \times I(M_2(a^*) = m_2)\\
&= \sum_{m_2}\sum_{m_1}[Y(a, m_1, m_2) - Y(a^*, m_1, m_2)] \times I(M_1(a^*) = m_1) \times I(M_2(a^*) = m_2)\\
&= \sum_{m_2}\sum_{m_1}[Y(a, m_1, m_2) - Y(a^*, m_1, m_2) - Y(a, m_1^*, m_2^*) + Y(a^*, m_1^*, m_2^*)] \times I(M_1(a^*) = m_1) \times I(M_2(a^*) = m_2)\\
&\quad + \sum_{m_2}\sum_{m_1}[Y(a, m_1^*, m_2^*) - Y(a^*, m_1^*, m_2^*)] \times I(M_1(a^*) = m_1) \times I(M_2(a^*) = m_2)
\end{aligned}$$

$$\begin{aligned}
&= \sum_{m_2}\sum_{m_1}[Y(a, m_1, m_2) - Y(a^*, m_1, m_2) - Y(a, m_1^*, m_2^*) + Y(a^*, m_1^*, m_2^*)] \times I(M_1(a^*) = m_1) \times I(M_2(a^*) = m_2)\\
&\quad + Y(a, m_1^*, m_2^*) - Y(a^*, m_1^*, m_2^*)\\
&= \sum_{m_2}\sum_{m_1}[Y(a, m_1, m_2) - Y(a^*, m_1, m_2) - Y(a, m_1^*, m_2^*) + Y(a^*, m_1^*, m_2^*) + Y(a^*, m_1^*, m_2^*) - Y(a^*, m_1^*, m_2^*)\\
&\quad + Y(a^*, m_1^*, m_2) - Y(a^*, m_1^*, m_2) + Y(a^*, m_1, m_2^*) - Y(a^*, m_1, m_2^*) + Y(a, m_1^*, m_2^*) - Y(a, m_1^*, m_2^*)\\
&\quad + Y(a, m_1^*, m_2) - Y(a, m_1^*, m_2) + Y(a, m_1, m_2^*) - Y(a, m_1, m_2^*)]\\
&\quad \times I(M_1(a^*) = m_1) \times I(M_2(a^*) = m_2) + Y(a, m_1^*, m_2^*) - Y(a^*, m_1^*, m_2^*)
\end{aligned}$$

$$\begin{aligned}
\text{PDE} &= \sum_{m_2}\sum_{m_1}[Y(a, m_1, m_2^*) - Y(a^*, m_1, m_2^*) - Y(a, m_1^*, m_2^*) + Y(a^*, m_1^*, m_2^*)] \times I(M_1(a^*) = m_1)I(M_2(a^*) = m_2)\\
&\quad + \sum_{m_2}\sum_{m_1}[Y(a, m_1^*, m_2) - Y(a^*, m_1^*, m_2) - Y(a, m_1^*, m_2^*) + Y(a^*, m_1^*, m_2^*)] \times I(M_1(a^*) = m_1)I(M_2(a^*) = m_2)\\
&\quad + \sum_{m_2}\sum_{m_1}[Y(a, m_1, m_2) - Y(a^*, m_1, m_2) - Y(a, m_1^*, m_2) + Y(a^*, m_1^*, m_2) - Y(a, m_1, m_2^*) + Y(a^*, m_1, m_2^*)\\
&\quad + Y(a, m_1^*, m_2^*) - Y(a^*, m_1^*, m_2^*)] \times I(M_1(a^*) = m_1) \times I(M_2(a^*) = m_2) + Y(a, m_1^*, m_2^*) - Y(a^*, m_1^*, m_2^*)\\
&= \sum_{m_1}[Y(a, m_1, m_2^*) - Y(a^*, m_1, m_2^*) - Y(a, m_1^*, m_2^*) + Y(a^*, m_1^*, m_2^*)] \times I(M_1(a^*) = m_1)\\
&\quad + \sum_{m_2}[Y(a, m_1^*, m_2) - Y(a^*, m_1^*, m_2) - Y(a, m_1^*, m_2^*) + Y(a^*, m_1^*, m_2^*)] \times I(M_2(a^*) = m_2)\\
&\quad + \sum_{m_2}\sum_{m_1}[Y(a, m_1, m_2) - Y(a^*, m_1, m_2) - Y(a, m_1^*, m_2) + Y(a^*, m_1^*, m_2)\\
&\quad - Y(a, m_1, m_2^*) + Y(a^*, m_1, m_2^*) + Y(a, m_1^*, m_2^*) - Y(a^*, m_1^*, m_2^*)]\\
&\quad \times I(M_1(a^*) = m_1) \times I(M_2(a^*) = m_2) + Y(a, m_1^*, m_2^*) - Y(a^*, m_1^*, m_2^*).
\end{aligned}$$

According to the derivation above, the following formulas can be obtained:

$$\begin{aligned}
\text{CDE}(m_1^*, m_2^*) &= Y(a, m_1^*, m_2^*) - Y(a^*, m_1^*, m_2^*),\\
\text{INT}_{\text{ref-AM}_1}(m_1^*, m_2^*) &= \sum_{m_1}[Y(a, m_1, m_2^*) - Y(a^*, m_1, m_2^*) - Y(a, m_1^*, m_2^*) + Y(a^*, m_1^*, m_2^*)] \times I(M_1(a^*) = m_1),\\
\text{INT}_{\text{ref-AM}_2}(m_1^*, m_2^*) &= \sum_{m_2}[Y(a, m_1^*, m_2) - Y(a^*, m_1^*, m_2) - Y(a, m_1^*, m_2^*) + Y(a^*, m_1^*, m_2^*)] \times I(M_2(a^*) = m_2),\\
\text{INT}_{\text{ref-AM}_1M_2}(m_1^*, m_2^*) &= \sum_{m_2}\sum_{m_1}[Y(a, m_1, m_2) - Y(a^*, m_1, m_2) - Y(a, m_1^*, m_2) + Y(a^*, m_1^*, m_2)\\
&\quad - Y(a, m_1, m_2^*) + Y(a^*, m_1, m_2^*) + Y(a, m_1^*, m_2^*) - Y(a^*, m_1^*, m_2^*)]\\
&\quad \times I(M_1(a^*) = m_1) \times I(M_2(a^*) = m_2).
\end{aligned}$$

With a little mathematical derivation, $\text{INT}_{\text{ref-AM}_1}$, $\text{INT}_{\text{ref-AM}_2}$, and $\text{INT}_{\text{ref-AM}_1M_2}$ can be expressed in the form of the counterfactual formula:

$$\begin{aligned}
\text{INT}_{\text{ref-AM}_1}(m_1^*, m_2^*) &= Y(a, M_1(a^*), m_2^*) - Y(a^*, M_1(a^*), m_2^*) - Y(a, m_1^*, m_2^*) + Y(a^*, m_1^*, m_2^*),\\
\text{INT}_{\text{ref-AM}_2}(m_1^*, m_2^*) &= Y(a, m_1^*, M_2(a^*)) - Y(a^*, m_1^*, M_2(a^*)) - Y(a, m_1^*, m_2^*) + Y(a^*, m_1^*, m_2^*),\\
\text{INT}_{\text{ref-AM}_1M_2}(m_1^*, m_2^*) &= Y(a, M_1(a^*), M_2(a^*)) - Y(a^*, M_1(a^*), M_2(a^*)) - Y(a, m_1^*, M_2(a^*)) + Y(a^*, m_1^*, M_2(a^*)),\\
&\quad -Y(a, M_1(a^*), m_2^*) + Y(a^*, M_1(a^*), m_2^*) + Y(a, m_1^*, m_2^*) - Y(a^*, m_1^*, m_2^*).
\end{aligned}$$

Therefore, PDE can be decomposed into the following components:

$$\text{PDE} = \text{CDE}(m_1^*, m_2^*) + \text{INT}_{\text{ref-AM}_1}(m_1^*, m_2^*) + \text{INT}_{\text{ref-AM}_2}(m_1^*, m_2^*) + \text{INT}_{\text{ref-AM}_1M_2}(m_1^*, m_2^*).$$

We next focus on NIE_{M_1} and try to decompose it into PIE_{M_1} and $\text{NatINT}_{M_1M_2}$ by subtracting PIE_{M_1} from NIE_{M_1}:

$$\begin{aligned}
\text{NIE}_{M_1} - \text{PIE}_{M_1} &= Y(a^*, M_1(a), M_2(a)) - Y(a^*, M_1(a^*), M_2(a)) - Y(a^*, M_1(a), M_2(a^*)) + Y(a^*, M_1(a^*), M_2(a^*))\\
&= \text{NatINT}_{M_1M_2},
\end{aligned}$$

where $\text{NatINT}_{M_1M_2}$ satisfies Definition 2.

Therefore, NIE_{M_1} can be decomposed into the following components:

$$\text{NIE}_{M_1} = \text{PIE}_{M_1} + \text{NatINT}_{M_1M_2}.$$

Combining all the derivations above, we have the decomposition of TE as follows:

$$\begin{aligned}
\text{TE} = {} & \text{CDE}(m_1^*, m_2^*) + \text{INT}_{\text{ref-AM}_1}(m_1^*, m_2^*) + \text{INT}_{\text{ref-AM}_2}(m_1^*, m_2^*) + \text{INT}_{\text{ref-AM}_1M_2}(m_1^*, m_2^*)\\
& + \text{NatINT}_{AM_1} + \text{NatINT}_{AM_2} + \text{NatINT}_{AM_1M_2} + \text{NatINT}_{M_1M_2} + \text{PIE}_{M_1} + \text{PIE}_{M_2}.
\end{aligned}$$

We next present the interpretation for each component assuming binary A, M_1, and M_2 with the conditions $a = 1$, $a^* = 0$, $m_1^* = 0$, and $m_2^* = 0$ for illustration purpose. While other interpretations were proposed in the literature [7,9], our work represents a different and more flexible interpretation from the perspective of population averages which accounts for the distribution of the mediators in the causal structure.

S1.1 CDE

With the specified conditions, the CDE can be written as:

$$\text{CDE}(m_1^*, m_2^*) = Y(a, m_1^*, m_2^*) - Y(a^*, m_1^*, m_2^*) \Rightarrow \text{CDE}(0, 0) = Y(1, 0, 0) - Y(0, 0, 0).$$

$\text{CDE}(m_1^*, m_2^*)$ can be interpreted as the effect due to neither mediation nor interaction.

S1.2 Reference interaction effects

With the specified conditions, the reference interaction effect between A and M_1 can be written as:

$$\begin{aligned}
\text{INT}_{\text{ref-AM}_1}(m_1^*, m_2^*) &= \sum_{m_1}[Y(a, m_1, m_2^*) - Y(a^*, m_1, m_2^*) - Y(a, m_1^*, m_2^*) + Y(a^*, m_1^*, m_2^*)] \times I(M_1(a^*) = m_1) \\
\Rightarrow \text{INT}_{\text{ref-AM}_1}(0, 0) &= \sum_{m_1}[Y(1, m_1, 0) - Y(0, m_1, 0) - Y(1, 0, 0) + Y(0, 0, 0)] \times I(M_1(0) = m_1) \\
&= [Y(1, 0, 0) - Y(0, 0, 0) - Y(1, 0, 0) + Y(0, 0, 0)] \times I(M_1(0) = 0) \\
&\quad + [Y(1, 1, 0) - Y(0, 1, 0) - Y(1, 0, 0) + Y(0, 0, 0)] \times I(M_1(0) = 1) \\
&= [Y(1, 1, 0) - Y(0, 1, 0) - Y(1, 0, 0) + Y(0, 0, 0)] \times I(M_1(0) = 1) \\
&= [Y(1, 1, 0) - Y(0, 1, 0) - Y(1, 0, 0) + Y(0, 0, 0)] \times M_1(0).
\end{aligned}$$

$\text{INT}_{\text{ref-AM}_1}(m_1^*, m_2^*)$ can be interpreted as the effect due to the interaction between A and M_1 only.

The reference interaction effect between A and M_2 can be written as:

$$\begin{aligned}
\text{INT}_{\text{ref-AM}_2}(m_1^*, m_2^*) &= \sum_{m_2}[Y(a, m_1^*, m_2) - Y(a^*, m_1^*, m_2) - Y(a, m_1^*, m_2^*) + Y(a^*, m_1^*, m_2^*)] \times I(M_2(a^*) = m_2) \\
\Rightarrow \text{INT}_{\text{ref-AM}_2}(0, 0) &= \sum_{m_2}[Y(1, 0, m_2) - Y(0, 0, m_2) - Y(1, 0, 0) + Y(0, 0, 0)] \times I(M_2(0) = m_2) \\
&= [Y(1, 0, 0) - Y(0, 0, 0) - Y(1, 0, 0) + Y(0, 0, 0)] \times I(M_2(0) = 0) \\
&\quad + [Y(1, 0, 1) - Y(0, 0, 1) - Y(1, 0, 0) + Y(0, 0, 0)] \times I(M_2(0) = 1) \\
&= [Y(1, 0, 1) - Y(0, 0, 1) - Y(1, 0, 0) + Y(0, 0, 0)] \times I(M_2(0) = 1) \\
&= [Y(1, 0, 1) - Y(0, 0, 1) - Y(1, 0, 0) + Y(0, 0, 0)] \times M_2(0).
\end{aligned}$$

$\text{INT}_{\text{ref-AM}_2}(m_1^*, m_2^*)$ can be interpreted as the effect due to the interaction between A and M_2 only.

The reference interaction effect between A, M_1, and M_2 can be written as:

$$\begin{aligned}
\text{INT}_{\text{ref-AM}_1M_2}(m_1^*, m_2^*) &= \sum_{m_2}\sum_{m_1}[Y(a, m_1, m_2) - Y(a^*, m_1, m_2) - Y(a, m_1^*, m_2) + Y(a^*, m_1^*, m_2) \\
&\quad - Y(a, m_1, m_2^*) + Y(a^*, m_1, m_2^*) + Y(a, m_1^*, m_2^*) - Y(a^*, m_1^*, m_2^*)] \\
&\quad \times I(M_1(a^*) = m_1) \times I(M_2(a^*) = m_2) \\
\Rightarrow \text{INT}_{\text{ref-AM}_1M_2}(0, 0) &= \sum_{m_2}\sum_{m_1}[Y(1, m_1, m_2) - Y(0, m_1, m_2) - Y(1, 0, m_2) + Y(0, 0, m_2) - Y(1, m_1, 0) \\
&\quad + Y(0, m_1, 0) + Y(1, 0, 0) - Y(0, 0, 0)] \times I(M_1(0) = m_1) \times I(M_2(0) = m_2) \\
&= \sum_{m_2}[Y(1, 0, m_2) - Y(0, 0, m_2) - Y(1, 0, m_2) + Y(0, 0, m_2) - Y(1, 0, 0) + Y(0, 0, 0) \\
&\quad + Y(1, 0, 0) - Y(0, 0, 0)] \times I(M_1(0) = 0) \times I(M_2(0) = m_2) \\
&\quad + \sum_{m_2}[Y(1, 1, m_2) - Y(0, 1, m_2) - Y(1, 0, m_2) + Y(0, 0, m_2) - Y(1, 1, 0) + Y(0, 1, 0) \\
&\quad + Y(1, 0, 0) - Y(0, 0, 0)] \times I(M_1(0) = 1) \times I(M_2(0) = m_2) \\
&= [Y(1, 1, 0) - Y(0, 1, 0) - Y(1, 0, 0) + Y(0, 0, 0) - Y(1, 1, 0) + Y(0, 1, 0) + Y(1, 0, 0) \\
&\quad - Y(0, 0, 0)] \times I(M_1(0) = 1) \times I(M_2(0) = 0) + [Y(1, 1, 1) - Y(0, 1, 1) - Y(1, 0, 1) \\
&\quad + Y(0, 0, 1) - Y(1, 1, 0) + Y(0, 1, 0) + Y(1, 0, 0) - Y(0, 0, 0)] \times I(M_1(0) = 1) \\
&\quad \times I(M_2(0) = 1) \\
&= [Y(1, 1, 1) - Y(0, 1, 1) - Y(1, 0, 1) + Y(0, 0, 1) - Y(1, 1, 0) + Y(0, 1, 0) + Y(1, 0, 0) \\
&\quad - Y(0, 0, 0)] \times M_1(0) \times M_2(0).
\end{aligned}$$

$\text{INT}_{\text{ref-AM}_1M_2}(m_1^*, m_2^*)$ can be interpreted as the effect due to the interaction between A, M_1, and M_2 only.

S1.3 Natural MI effects

The natural MI effect between A and M_1 can be rewritten as:

$$\begin{aligned}
\text{NatINT}_{AM_1} &= Y(a, M_1(a), M_2(a^*)) - Y(a^*, M_1(a), M_2(a^*)) - Y(a, M_1(a^*), M_2(a^*)) + Y(a^*, M_1(a^*), M_2(a^*)) \\
&= \sum_{m_2}\sum_{m_1} Y(a, m_1, m_2) I(M_1(a) = m_1) I(M_2(a^*) = m_2) - \sum_{m_2}\sum_{m_1} Y(a^*, m_1, m_2) I(M_1(a) = m_1) I(M_2(a^*) = m_2) \\
&\quad - \sum_{m_2}\sum_{m_1} Y(a, m_1, m_2) I(M_1(a^*) = m_1) I(M_2(a^*) = m_2) + \sum_{m_2}\sum_{m_1} Y(a^*, m_1, m_2) I(M_1(a^*) = m_1) I(M_2(a^*) = m_2) \\
&= \sum_{m_2}\sum_{m_1} [Y(a, m_1, m_2) - Y(a^*, m_1, m_2)] I(M_1(a) = m_1) I(M_2(a^*) = m_2) \\
&\quad - \sum_{m_2}\sum_{m_1} [Y(a, m_1, m_2) - Y(a^*, m_1, m_2)] I(M_1(a^*) = m_1) I(M_2(a^*) = m_2) \\
&= \sum_{m_2}\sum_{m_1} [Y(a, m_1, m_2) - Y(a^*, m_1, m_2)] I(M_2(a^*) = m_2) [I(M_1(a) = m_1) - I(M_1(a^*) = m_1)] \\
&= \sum_{m_2}\sum_{m_1} [Y(a, m_1, m_2) I(M_2(a^*) = m_2) - Y(a^*, m_1, m_2) I(M_2(a^*) = m_2)] \\
&\quad \times [I(M_1(a) = m_1) - I(M_1(a^*) = m_1)] \\
&= \sum_{m_2}\sum_{m_1} [Y(a, m_1, m_2) I(M_2(a^*) = m_2) - Y(a^*, m_1, m_2) I(M_2(a^*) = m_2) - Y(a, m_1^*, m_2) I(M_2(a^*) = m_2) \\
&\quad + Y(a^*, m_1^*, m_2) I(M_2(a^*) = m_2)] \times [I(M_1(a) = m_1) - I(M_1(a^*) = m_1)],
\end{aligned}$$

where the sixth equation follows by adding two extra terms which do not change the value of NatINT_{AM_1}.

With the specified conditions, NatINT_{AM_1} can be written as:

$$\begin{aligned}
\text{NatINT}_{AM_1} &= \sum_{m_2}\sum_{m_1} [Y(1, m_1, m_2) I(M_2(0) = m_2) - Y(0, m_1, m_2) I(M_2(0) = m_2) - Y(1, 0, m_2) I(M_2(0) = m_2) \\
&\quad + Y(0, 0, m_2) I(M_2(0) = m_2)] \times [I(M_1(1) = m_1) - I(M_1(0) = m_1)] \\
&= \sum_{m_2} [Y(1, 0, m_2) I(M_2(0) = m_2) - Y(0, 0, m_2) I(M_2(0) = m_2) - Y(1, 0, m_2) I(M_2(0) = m_2) \\
&\quad + Y(0, 0, m_2) I(M_2(0) = m_2)] \times [I(M_1(1) = 0) - I(M_1(0) = 0)] \\
&\quad + \sum_{m_2} [Y(1, 1, m_2) I(M_2(0) = m_2) - Y(0, 1, m_2) I(M_2(0) = m_2) - Y(1, 0, m_2) I(M_2(0) = m_2) \\
&\quad + Y(0, 0, m_2) I(M_2(0) = m_2)] \times [I(M_1(1) = 1) - I(M_1(0) = 1)] \\
&= \sum_{m_2} [Y(1, 1, m_2) I(M_2(0) = m_2) - Y(0, 1, m_2) I(M_2(0) = m_2) - Y(1, 0, m_2) I(M_2(0) = m_2) \\
&\quad + Y(0, 0, m_2) I(M_2(0) = m_2)] \times [I(M_1(1) = 1) - I(M_1(0) = 1)] \\
&= \sum_{m_2} [Y(1, 1, m_2) I(M_2(0) = m_2) - Y(0, 1, m_2) I(M_2(0) = m_2) - Y(1, 0, m_2) I(M_2(0) = m_2) \\
&\quad + Y(0, 0, m_2) I(M_2(0) = m_2)] \times [M_1(1) - M_1(0)],
\end{aligned}$$

where the indicator function $I(M_2(0) = m_2)$ indicates that M_2 is at its potential value $M_2(0)$, which may vary with respect to different individuals.

NatINT_{AM_1} can be interpreted as the effect due to the mediation through M_1 and the interaction between A and M_1 conditioning on the potential value of M_2 with the fixed reference level a^*.

The natural MI effect between A and M_2 can be rewritten as:

$$\begin{aligned}\text{NatINT}_{AM_2} &= Y(a, M_1(a^*), M_2(a)) - Y(a^*, M_1(a^*), M_2(a)) - Y(a, M_1(a^*), M_2(a^*)) + Y(a^*, M_1(a^*), M_2(a^*))\\
&= \sum_{m_2}\sum_{m_1} Y(a, m_1, m_2)I(M_1(a^*) = m_1)I(M_2(a) = m_2) - \sum_{m_2}\sum_{m_1} Y(a^*, m_1, m_2)I(M_1(a^*) = m_1)I(M_2(a) = m_2)\\
&\quad - \sum_{m_2}\sum_{m_1} Y(a, m_1, m_2)I(M_1(a^*) = m_1)I(M_2(a^*) = m_2) + \sum_{m_2}\sum_{m_1} Y(a^*, m_1, m_2)I(M_1(a^*) = m_1)I(M_2(a^*) = m_2)\\
&= \sum_{m_2}\sum_{m_1}[Y(a, m_1, m_2) - Y(a^*, m_1, m_2)]I(M_1(a^*) = m_1)I(M_2(a) = m_2)\\
&\quad - \sum_{m_2}\sum_{m_1}[Y(a, m_1, m_2) - Y(a^*, m_1, m_2)]I(M_1(a^*) = m_1)I(M_2(a^*) = m_2)\\
&= \sum_{m_2}\sum_{m_1}[Y(a, m_1, m_2) - Y(a^*, m_1, m_2)]I(M_1(a^*) = m_1)[I(M_2(a) = m_2) - I(M_2(a^*) = m_2)]\\
&= \sum_{m_2}\sum_{m_1}[Y(a, m_1, m_2)I(M_1(a^*) = m_1) - Y(a^*, m_1, m_2)I(M_1(a^*) = m_1)]\\
&\quad \times [I(M_2(a) = m_2) - I(M_2(a^*) = m_2)]\\
&= \sum_{m_2}\sum_{m_1}[Y(a, m_1, m_2)I(M_1(a^*) = m_1) - Y(a^*, m_1, m_2)I(M_1(a^*) = m_1) - Y(a, m_1, m_2^*)I(M_1(a^*) = m_1)\\
&\quad + Y(a^*, m_1, m_2^*)I(M_1(a^*) = m_1)] \times [I(M_2(a) = m_2) - I(M_2(a^*) = m_2)],\end{aligned}$$

where the sixth equation follows by adding two extra terms which do not change the value of NatINT_{AM_2}.

With the specified conditions, NatINT_{AM_2} can be written as:

$$\begin{aligned}\text{NatINT}_{AM_2} &= \sum_{m_2}\sum_{m_1}[Y(1, m_1, m_2)I(M_1(0) = m_1) - Y(0, m_1, m_2)I(M_1(0) = m_1) - Y(1, m_1, 0)I(M_1(0) = m_1)\\
&\quad + Y(0, m_1, 0)I(M_1(0) = m_1)] \times [I(M_2(1) = m_2) - I(M_2(0) = m_2)]\\
&= \sum_{m_1}[Y(1, m_1, 0)I(M_1(0) = m_1) - Y(0, m_1, 0)I(M_1(0) = m_1) - Y(1, m_1, 0)I(M_1(0) = m_1)\\
&\quad + Y(0, m_1, 0)I(M_1(0) = m_1)] \times [I(M_2(1) = 0) - I(M_2(0) = 0)]\\
&\quad + \sum_{m_1}[Y(1, m_1, 1)I(M_1(0) = m_1) - Y(0, m_1, 1)I(M_1(0) = m_1) - Y(1, m_1, 0)I(M_1(0) = m_1)\\
&\quad + Y(0, m_1, 0)I(M_1(0) = m_1)] \times [I(M_2(1) = 1) - I(M_2(0) = 1)]\\
&= \sum_{m_1}[Y(1, m_1, 1)I(M_1(0) = m_1) - Y(0, m_1, 1)I(M_1(0) = m_1) - Y(1, m_1, 0)I(M_1(0) = m_1)\\
&\quad + Y(0, m_1, 0)I(M_1(0) = m_1)] \times [I(M_2(1) = 1) - I(M_2(0) = 1)]\\
&= \sum_{m_1}[Y(1, m_1, 1)I(M_1(0) = m_1) - Y(0, m_1, 1)I(M_1(0) = m_1) - Y(1, m_1, 0)I(M_1(0) = m_1)\\
&\quad + Y(0, m_1, 0)I(M_1(0) = m_1)] \times [M_2(1) - M_2(0)],\end{aligned}$$

where the indicator function $I(M_1(0) = m_1)$ indicates that M_1 is at its potential value $M_1(0)$, which may vary with respect to different individuals.

NatINT_{AM_2} can be interpreted as the effect due to the mediation through M_2 and the interaction between A and M_2 conditioning on the potential value of M_1 with the fixed reference level a^*.

The natural MI effect between A, M_1, and M_2 can be rewritten as:

$$\begin{aligned}\text{NatINT}_{AM_1M_2} &= Y(a, M_1(a), M_2(a)) - Y(a^*, M_1(a), M_2(a)) - Y(a, M_1(a^*), M_2(a)) + Y(a^*, M_1(a^*), M_2(a))\\
&\quad - Y(a, M_1(a), M_2(a^*)) + Y(a^*, M_1(a), M_2(a^*)) + Y(a, M_1(a^*), M_2(a^*)) - Y(a^*, M_1(a^*), M_2(a^*))\\
&= \sum_{m_2}\sum_{m_1} Y(a, m_1, m_2)I(M_1(a) = m_1)I(M_2(a) = m_2) - \sum_{m_2}\sum_{m_1} Y(a^*, m_1, m_2)I(M_1(a) = m_1)I(M_2(a) = m_2)\\
&\quad - \sum_{m_2}\sum_{m_1} Y(a, m_1, m_2)I(M_1(a^*) = m_1)I(M_2(a) = m_2) + \sum_{m_2}\sum_{m_1} Y(a^*, m_1, m_2)I(M_1(a^*) = m_1)I(M_2(a) = m_2)\\
&\quad - \sum_{m_2}\sum_{m_1} Y(a, m_1, m_2)I(M_1(a) = m_1)I(M_2(a^*) = m_2) + \sum_{m_2}\sum_{m_1} Y(a^*, m_1, m_2)I(M_1(a) = m_1)I(M_2(a^*) = m_2)\\
&\quad + \sum_{m_2}\sum_{m_1} Y(a, m_1, m_2)I(M_1(a^*) = m_1)I(M_2(a^*) = m_2) - \sum_{m_2}\sum_{m_1} Y(a^*, m_1, m_2)I(M_1(a^*) = m_1)I(M_2(a^*) = m_2)\end{aligned}$$

$$\begin{aligned}
\mathrm{NatINT}_{AM_1M_2} &= \sum_{m_2}\sum_{m_1}[Y(a, m_1, m_2) - Y(a^*, m_1, m_2)]I(M_1(a) = m_1)I(M_2(a) = m_2)\\
&\quad - \sum_{m_2}\sum_{m_1}[Y(a, m_1, m_2) - Y(a^*, m_1, m_2)]I(M_1(a^*) = m_1)I(M_2(a) = m_2)\\
&\quad - \sum_{m_2}\sum_{m_1}[Y(a, m_1, m_2) - Y(a^*, m_1, m_2)]I(M_1(a) = m_1)I(M_2(a^*) = m_2)\\
&\quad + \sum_{m_2}\sum_{m_1}[Y(a, m_1, m_2) - Y(a^*, m_1, m_2)]I(M_1(a^*) = m_1)I(M_2(a^*) = m_2)\\
&= \sum_{m_2}\sum_{m_1}[Y(a, m_1, m_2) - Y(a^*, m_1, m_2)][I(M_1(a) = m_1) - I(M_1(a^*) = m_1)]\\
&\quad \times [I(M_2(a) = m_2) - I(M_2(a^*) = m_2)]\\
&= \sum_{m_2}\sum_{m_1}[Y(a, m_1, m_2) - Y(a^*, m_1, m_2) - Y(a, m_1^*, m_2) + Y(a^*, m_1^*, m_2) - Y(a, m_1, m_2^*) + Y(a^*, m_1, m_2^*)\\
&\quad + Y(a, m_1^*, m_2^*) - Y(a^*, m_1^*, m_2^*)]\\
&\quad \times [I(M_1(a) = m_1) - I(M_1(a^*) = m_1)] \times [I(M_2(a) = m_2) - I(M_2(a^*) = m_2)],
\end{aligned}$$

where the fifth equation follows by adding six extra terms which do not change the value of $\mathrm{NatINT}_{AM_1M_2}$.

With the specified conditions, $\mathrm{NatINT}_{AM_1M_2}$ can be written as:

$$\begin{aligned}
\mathrm{NatINT}_{AM_1M_2} &= \sum_{m_2}\sum_{m_1}[Y(1, m_1, m_2) - Y(0, m_1, m_2) - Y(1, 0, m_2) + Y(0, 0, m_2) - Y(1, m_1, 0) + Y(0, m_1, 0)\\
&\quad + Y(1, 0, 0) - Y(0, 0, 0)] \times [I(M_1(1) = m_1) - I(M_1(0) = m_1)]\\
&\quad \times [I(M_2(1) = m_2) - I(M_2(0) = m_2)]\\
&= \sum_{m_2}[Y(1, 0, m_2) - Y(0, 0, m_2) - Y(1, 0, m_2) + Y(0, 0, m_2) - Y(1, 0, 0) + Y(0, 0, 0) + Y(1, 0, 0)\\
&\quad - Y(0, 0, 0)] \times [I(M_1(1) = 0) - I(M_1(0) = 0)] \times [I(M_2(1) = m_2) - I(M_2(0) = m_2)]\\
&\quad + \sum_{m_2}[Y(1, 1, m_2) - Y(0, 1, m_2) - Y(1, 0, m_2) + Y(0, 0, m_2) - Y(1, 1, 0) + Y(0, 1, 0) + Y(1, 0, 0)\\
&\quad - Y(0, 0, 0)] \times [I(M_1(1) = 1) - I(M_1(0) = 1)] \times [I(M_2(1) = m_2) - I(M_2(0) = m_2)]\\
&= [Y(1, 1, 0) - Y(0, 1, 0) - Y(1, 0, 0) + Y(0, 0, 0) - Y(1, 1, 0) + Y(0, 1, 0) + Y(1, 0, 0)\\
&\quad - Y(0, 0, 0)] \times [I(M_1(1) = 1) - I(M_1(0) = 1)] \times [I(M_2(1) = 0) - I(M_2(0) = 0)]\\
&\quad + [Y(1, 1, 1) - Y(0, 1, 1) - Y(1, 0, 1) + Y(0, 0, 1) - Y(1, 1, 0) + Y(0, 1, 0) + Y(1, 0, 0)\\
&\quad - Y(0, 0, 0)] \times [I(M_1(1) = 1) - I(M_1(0) = 1)] \times [I(M_2(1) = 1) - I(M_2(0) = 1)]\\
&= [Y(1, 1, 1) - Y(0, 1, 1) - Y(1, 0, 1) + Y(0, 0, 1) - Y(1, 1, 0) + Y(0, 1, 0) + Y(1, 0, 0)\\
&\quad - Y(0, 0, 0)] \times [I(M_1(1) = 1) - I(M_1(0) = 1)] \times [I(M_2(1) = 1) - I(M_2(0) = 1)]\\
&= [Y(1, 1, 1) - Y(0, 1, 1) - Y(1, 0, 1) + Y(0, 0, 1) - Y(1, 1, 0) + Y(0, 1, 0) + Y(1, 0, 0)\\
&\quad - Y(0, 0, 0)] \times [M_1(1) - M_1(0)] \times [M_2(1) - M_2(0)],
\end{aligned}$$

$\mathrm{NatINT}_{AM_1M_2}$ can be interpreted as the effect due to the mediation through both M_1 and M_2, and the interaction between A, M_1, and M_2.

The natural MI effect between M_1 and M_2 can be rewritten as:

$$\begin{aligned}
\mathrm{NatINT}_{M_1M_2} &= Y(a^*, M_1(a), M_2(a)) - Y(a^*, M_1(a^*), M_2(a)) - Y(a^*, M_1(a), M_2(a^*)) + Y(a^*, M_1(a^*), M_2(a^*))\\
&= \sum_{m_2}\sum_{m_1}Y(a^*, m_1, m_2)I(M_1(a) = m_1)I(M_2(a) = m_2) - \sum_{m_2}\sum_{m_1}Y(a^*, m_1, m_2)I(M_1(a^*) = m_1)I(M_2(a) = m_2)\\
&\quad - \sum_{m_2}\sum_{m_1}Y(a^*, m_1, m_2)I(M_1(a) = m_1)I(M_2(a^*) = m_2) + \sum_{m_2}\sum_{m_1}Y(a^*, m_1, m_2)I(M_1(a^*) = m_1)I(M_2(a^*) = m_2)\\
&= \sum_{m_2}\sum_{m_1}Y(a^*, m_1, m_2)[I(M_1(a) = m_1) - I(M_1(a^*) = m_1)] \times [I(M_2(a) = m_2) - I(M_2(a^*) = m_2)]\\
&= \sum_{m_2}\sum_{m_1}[Y(a^*, m_1, m_2) - Y(a^*, m_1^*, m_2) - Y(a^*, m_1, m_2^*) + Y(a^*, m_1^*, m_2^*)]\\
&\quad \times [I(M_1(a) = m_1) - I(M_1(a^*) = m_1)] \times [I(M_2(a) = m_2) - I(M_2(a^*) = m_2)],
\end{aligned}$$

where the fourth equation follows by adding three extra terms which do not change the value of $\mathrm{NatINT}_{M_1M_2}$.

With the specified conditions, $\mathrm{NatINT}_{M_1M_2}$ can be written as:

$$\begin{aligned}
\text{NatINT}_{M_1M_2} &= \sum_{m_2}\sum_{m_1}[Y(0,m_1,m_2) - Y(0,0,m_2) - Y(0,m_1,0) + Y(0,0,0)] \times [I(M_1(1)=m_1) - I(M_1(0)=m_1)] \\
&\quad \times [I(M_2(1)=m_2) - I(M_2(0)=m_2)] \\
&= \sum_{m_2}[Y(0,0,m_2) - Y(0,0,m_2) - Y(0,0,0) + Y(0,0,0)] \times [I(M_1(1)=0) - I(M_1(0)=0)] \\
&\quad \times [I(M_2(1)=m_2) - I(M_2(0)=m_2)] + \sum_{m_2}[Y(0,1,m_2) - Y(0,0,m_2) - Y(0,1,0) \\
&\quad + Y(0,0,0)] \times [I(M_1(1)=1) - I(M_1(0)=1)] \times [I(M_2(1)=m_2) - I(M_2(0)=m_2)] \\
&= [Y(0,1,0) - Y(0,0,0) - Y(0,1,0) + Y(0,0,0)] \times [I(M_1(1)=1) - I(M_1(0)=1)] \\
&\quad \times [I(M_2(1)=0) - I(M_2(0)=0)] + [Y(0,1,1) - Y(0,0,1) - Y(0,1,0) \\
&\quad + Y(0,0,0)] \times [I(M_1(1)=1) - I(M_1(0)=1)] \times [I(M_2(1)=1) - I(M_2(0)=1)] \\
&= [Y(0,1,1) - Y(0,0,1) - Y(0,1,0) + Y(0,0,0)] \times [I(M_1(1)=1) - I(M_1(0)=1)] \\
&\quad \times [I(M_2(1)=1) - I(M_2(0)=1)] \\
&= [Y(0,1,1) - Y(0,0,1) - Y(0,1,0) + Y(0,0,0)] \times [M_1(1) - M_1(0)] \times [M_2(1) - M_2(0)].
\end{aligned}$$

$\text{NatINT}_{M_1M_2}$ can be interpreted as the effect due to the mediation through both M_1 and M_2, and the interaction between M_1 and M_2. Since the interaction is not involved with the change in exposure A, the interpretation can be simply put as the effect due to the mediation through both M_1 and M_2 only.

S1.4 PIEs

The PIE through M_1 can be rewritten as:

$$\begin{aligned}
\text{PIE}_{M_1} &= Y(a^*, M_1(a), M_2(a^*)) - Y(a^*, M_1(a^*), M_2(a^*)) \\
&= \sum_{m_2}\sum_{m_1} Y(a^*, m_1, m_2) I(M_1(a)=m_1) I(M_2(a^*)=m_2) - \sum_{m_2}\sum_{m_1} Y(a^*, m_1, m_2) I(M_1(a^*)=m_1) I(M_2(a^*)=m_2) \\
&= \sum_{m_2}\sum_{m_1} Y(a^*, m_1, m_2)[I(M_1(a)=m_1) - I(M_1(a^*)=m_1)] I(M_2(a^*)=m_2).
\end{aligned}$$

With the specified conditions, PIE_{M_1} can be written as:

$$\begin{aligned}
\text{PIE}_{M_1} &= \sum_{m_2}\sum_{m_1} Y(0, m_1, m_2)[I(M_1(1)=m_1) - I(M_1(0)=m_1)] I(M_2(0)=m_2) \\
&= \sum_{m_2} Y(0,0,m_2)[I(M_1(1)=0) - I(M_1(0)=0)] I(M_2(0)=m_2) \\
&\quad + \sum_{m_2} Y(0,1,m_2)[I(M_1(1)=1) - I(M_1(0)=1)] I(M_2(0)=m_2) \\
&= -\sum_{m_2} Y(0,0,m_2)[I(M_1(1)=1) - I(M_1(0)=1)] I(M_2(0)=m_2) \\
&\quad + \sum_{m_2} Y(0,1,m_2)[I(M_1(1)=1) - I(M_1(0)=1)] I(M_2(0)=m_2) \\
&= \sum_{m_2}[Y(0,1,m_2) - Y(0,0,m_2)][I(M_1(1)=1) - I(M_1(0)=1)] I(M_2(0)=m_2) \\
&= \sum_{m_2}[Y(0,1,m_2) I(M_2(0)=m_2) - Y(0,0,m_2) I(M_2(0)=m_2)][M_1(1) - M_1(0)],
\end{aligned}$$

where the third equation follows by the facts that $I(M_1(1)=0) = 1 - I(M_1(1)=1)$ and $I(M_1(0)=0) = 1 - I(M_1(0)=1)$ and the indicator function, $I(M_2(0)=m_2)$, indicates that M_2 is at its potential value $M_2(0)$, which may vary with respect to different individuals.

PIE_{M_1} can be interpreted as the effect due to the mediation through M_1 only, conditioning on the potential value of M_2 with the fixed reference level a^*.

The PIE through M_2 can be rewritten as:

$$\begin{aligned}\mathrm{PIE}_{M_2} &= Y(a^*, M_1(a^*), M_2(a)) - Y(a^*, M_1(a^*), M_2(a^*))\\ &= \sum_{m_2}\sum_{m_1} Y(a^*, m_1, m_2)I(M_1(a^*) = m_1)I(M_2(a) = m_2) - \sum_{m_2}\sum_{m_1} Y(a^*, m_1, m_2)I(M_1(a^*) = m_1)I(M_2(a^*) = m_2)\\ &= \sum_{m_2}\sum_{m_1} Y(a^*, m_1, m_2)I(M_1(a^*) = m_1)[I(M_2(a) = m_2) - I(M_2(a^*) = m_2)].\end{aligned}$$

With the specified conditions, PIE_{M_2} can be written as:

$$\begin{aligned}\mathrm{PIE}_{M_2} &= \sum_{m_2}\sum_{m_1} Y(0, m_1, m_2)I(M_1(0) = m_1)[I(M_2(1) = m_2) - I(M_2(0) = m_2)]\\ &= \sum_{m_1} Y(0, m_1, 0)I(M_1(0) = m_1)[I(M_2(1) = 0) - I(M_2(0) = 0)]\\ &\quad + \sum_{m_1} Y(0, m_1, 1)I(M_1(0) = m_1)[I(M_2(1) = 1) - I(M_2(0) = 1)]\\ &= -\sum_{m_1} Y(0, m_1, 0)I(M_1(0) = m_1)[I(M_2(1) = 1) - I(M_2(0) = 1)]\\ &\quad + \sum_{m_1} Y(0, m_1, 1)I(M_1(0) = m_1)[I(M_2(1) = 1) - I(M_2(0) = 1)]\\ &= \sum_{m_1} [Y(0, m_1, 1)I(M_1(0) = m_1) - Y(0, m_1, 0)I(M_1(0) = m_1)][I(M_2(1) = 1) - I(M_2(0) = 1)]\\ &= \sum_{m_1} [Y(0, m_1, 1)I(M_1(0) = m_1) - Y(0, m_1, 0)I(M_1(0) = m_1)][M_2(1) - M_2(0)],\end{aligned}$$

where the third equation follows by the facts that $I(M_2(1) = 0) = 1 - I(M_2(1) = 1)$ and $I(M_2(0) = 0) = 1 - I(M_2(0) = 1)$ and the indicator function, $I(M_1(0) = m_1)$, indicates that M_1 is at its potential value $M_1(0)$, which may vary with respect to different individuals.

PIE_{M_2} can be interpreted as the effect due to the mediation through M_2 only, conditioning on the potential value of M_1 with the fixed reference level a^*.

S2 Mediated effects in a non-sequential two-mediator scenario in Bellavia's and Valeri's method

Suppose we have a directed acyclic graph as shown in Figure 3. We show that the mediated effects proposed by Bellavia and Valeri [9], if they exist (not equal to zero), are equivalent to the natural MI effects when A, M_1, and M_2 are binary with the conditions $a = 1$, $a^* = 0$, $m_1^* = 0$, $m_2^* = 0$ and the assumption $M_1(0) = M_2(0) = 0$, where the mediated effects consists of the MI effects and the pure NIEs.

S2.1 MI effect between A and M_1

From Supplementary material S1, we know that with the conditions $a = 1$, $a^* = 0$, $m_1^* = 0$, and $m_2^* = 0$ the NatINT_{AM_1} can be written as:

$$\begin{aligned}\mathrm{NatINT}_{AM_1} = \sum_{m_2} [&Y(1, 1, m_2)I(M_2(0) = m_2) - Y(0, 1, m_2)I(M_2(0) = m_2) - Y(1, 0, m_2)I(M_2(0) = m_2)\\ &+ Y(0, 0, m_2)I(M_2(0) = m_2)] \times [M_1(1) - M_1(0)].\end{aligned}$$

If we apply the condition $M_2(0) = 0$, the equation can be simplified to the following expression:

$$\begin{aligned}\text{NatINT}_{AM_1} &= [Y(1,1,0)I(M_2(0)=0) - Y(0,1,0)I(M_2(0)=0) - Y(1,0,0)I(M_2(0)=0)\\ &\quad + Y(0,0,0)I(M_2(0)=0)] \times [M_1(1) - M_1(0)] + [Y(1,1,1)I(M_2(0)=1) - Y(0,1,1)I(M_2(0)=1)\\ &\quad - Y(1,0,1)I(M_2(0)=1) + Y(0,0,1)I(M_2(0)=1)] \times [M_1(1) - M_1(0)]\\ &= [Y(1,1,0) - Y(0,1,0) - Y(1,0,0) + Y(0,0,0)] \times [M_1(1) - M_1(0)],\end{aligned}$$

where the second equality follows by the condition $M_2(0) = 0$. This expression is identical to the MI effect between A and M_1 proposed by Bellavia and Valeri [9].

S2.2 MI effect between A and M_2

From Supplementary material S1, we know that with the conditions $a = 1$, $a^* = 0$, $m_1^* = 0$, and $m_2^* = 0$ the NatINT_{AM_2} can be written as:

$$\begin{aligned}\text{NatINT}_{AM_2} &= \sum_{m_1}[Y(1,m_1,1)I(M_1(0)=m_1) - Y(0,m_1,1)I(M_1(0)=m_1) - Y(1,m_1,0)I(M_1(0)=m_1)\\ &\quad + Y(0,m_1,0)I(M_1(0)=m_1)] \times [M_2(1) - M_2(0)].\end{aligned}$$

If we apply the condition $M_1(0) = 0$, the equation can be simplified to the following expression:

$$\begin{aligned}\text{NatINT}_{AM_2} &= [Y(1,0,1)I(M_1(0)=0) - Y(0,0,1)I(M_1(0)=0) - Y(1,0,0)I(M_1(0)=0)\\ &\quad + Y(0,0,0)I(M_1(0)=0)] \times [M_2(1) - M_2(0)] + [Y(1,1,1)I(M_1(0)=1) - Y(0,1,1)I(M_1(0)=1)\\ &\quad - Y(1,1,0)I(M_1(0)=1) + Y(0,1,0)I(M_1(0)=1)] \times [M_2(1) - M_2(0)]\\ &= [Y(1,0,1) - Y(0,0,1) - Y(1,0,0) + Y(0,0,0)] \times [M_2(1) - M_2(0)],\end{aligned}$$

where the second equality follows by the condition $M_1(0) = 0$. This expression is identical to the MI effect between A and M_2 proposed by Bellavia and Valeri [9].

S2.3 MI effect between A, M_1, and M_2

From Supplementary material S1, we know that with the conditions $a = 1$, $a^* = 0$, $m_1^* = 0$, and $m_2^* = 0$ the $\text{NatINT}_{AM_1M_2}$ can be written as:

$$\begin{aligned}\text{NatINT}_{AM_1M_2} &= [Y(1,1,1) - Y(0,1,1) - Y(1,0,1) + Y(0,0,1) - Y(1,1,0) + Y(0,1,0) + Y(1,0,0)\\ &\quad - Y(0,0,0)] \times [M_1(1) - M_1(0)] \times [M_2(1) - M_2(0)].\end{aligned}$$

If we apply the conditions $M_1(0) = 0$ and $M_2(0) = 0$, the equation can be simplified to the following expression:

$$\begin{aligned}\text{NatINT}_{AM_1M_2} &= [Y(1,1,1) - Y(0,1,1) - Y(1,0,1) + Y(0,0,1) - Y(1,1,0) + Y(0,1,0) + Y(1,0,0)\\ &\quad - Y(0,0,0)] \times [M_1(1) - 0] \times [M_2(1) - 0]\\ &= [Y(1,1,1) - Y(0,1,1) - Y(1,0,1) + Y(0,0,1) - Y(1,1,0) + Y(0,1,0) + Y(1,0,0)\\ &\quad - Y(0,0,0)] \times M_1(1) \times M_2(1)\\ &= [Y(1,1,1) - Y(0,1,1) - Y(1,0,1) + Y(0,0,1) - Y(1,1,0) + Y(0,1,0) + Y(1,0,0)\\ &\quad - Y(0,0,0)] \times [M_1(1)M_2(1) - 0]\\ &= [Y(1,1,1) - Y(0,1,1) - Y(1,0,1) + Y(0,0,1) - Y(1,1,0) + Y(0,1,0) + Y(1,0,0)\\ &\quad - Y(0,0,0)] \times [M_1(1)M_2(1) - M_1(0)M_2(0)],\end{aligned}$$

where the last equality is identical to the MI effect between A, M_1, and M_2 proposed by Bellavia and Valeri [9].

S2.4 Pure NIE between M_1 and M_2 ($\text{PNIE}_{M_1M_2}$)

From Supplementary material S1, we know that with the conditions $a = 1$, $a^* = 0$, $m_1^* = 0$, and $m_2^* = 0$ the $\text{NatINT}_{M_1M_2}$ can be written as:

$$\text{NatINT}_{M_1M_2} = [Y(0,1,1) - Y(0,0,1) - Y(0,1,0) + Y(0,0,0)] \times [M_1(1) - M_1(0)] \times [M_2(1) - M_2(0)].$$

If we apply the conditions $M_1(0) = 0$ and $M_2(0) = 0$, the equation can be simplified to the following expression:

$$\begin{aligned}\text{NatINT}_{M_1M_2} &= [Y(0,1,1) - Y(0,0,1) - Y(0,1,0) + Y(0,0,0)] \times [M_1(1) - 0] \times [M_2(1) - 0]\\ &= [Y(0,1,1) - Y(0,0,1) - Y(0,1,0) + Y(0,0,0)] \times M_1(1) \times M_2(1)\\ &= [Y(0,1,1) - Y(0,0,1) - Y(0,1,0) + Y(0,0,0)] \times [M_1(1)M_2(1) - 0]\\ &= [Y(0,1,1) - Y(0,0,1) - Y(0,1,0) + Y(0,0,0)] \times [M_1(1)M_2(1) - M_1(0)M_2(0)],\end{aligned}$$

where the last equality is identical to the pure NIE between M_1 and M_2 ($\text{PNIE}_{M_1M_2}$) proposed by Bellavia and Valeri [9].

S2.5 PIE through M_1

From Supplementary material S1, we know that with the conditions $a = 1$, $a^* = 0$, $m_1^* = 0$, and $m_2^* = 0$ the PIE_{M_1} can be written as:

$$\text{PIE}_{M_1} = \sum_{m_2}[Y(0,1,m_2)I(M_2(0) = m_2) - Y(0,0,m_2)I(M_2(0) = m_2)][M_1(1) - M_1(0)].$$

If we apply the condition $M_2(0) = 0$, the equation can be simplified to the following expression:

$$\text{PIE}_{M_1} = [Y(0,1,0) - Y(0,0,0)][M_1(1) - M_1(0)],$$

where the equality is identical to the pure NIE through M_1 (PNIE_{M_1}) proposed by Bellavia and Valeri [9].

S2.6 PIE through M_2

From Supplementary material S1, we know that with the conditions $a = 1$, $a^* = 0$, $m_1^* = 0$, and $m_2^* = 0$ the PIE_{M_2} can be written as:

$$\text{PIE}_{M_2} = \sum_{m_1}[Y(0,m_1,1)I(M_1(0) = m_1) - Y(0,m_1,0)I(M_1(0) = m_1)][M_2(1) - M_2(0)].$$

If we apply the condition $M_1(0) = 0$, the equation can be simplified to the following expression:

$$\text{PIE}_{M_2} = [Y(0,0,1) - Y(0,0,0)][M_2(1) - M_2(0)],$$

where the equality is identical to the pure NIE through M_2 (PNIE_{M_2}) proposed by Bellavia and Valeri [9].

S2.7 Graphical comparison between the MI effect and the natural MI effect between A and M_1

With the conditions $a = 1$ and $a^* = 0$, the natural MI effect can be written as:

$$\text{NatINT}_{AM_1} = Y(1, M_1(1), M_2(0)) - Y(0, M_1(1), M_2(0)) - Y(1, M_1(0), M_2(0)) + Y(0, M_1(0), M_2(0)),$$

which is illustrated in Figure 4b.

If we apply the condition $M_2(0) = 0$, the natural MI effect will be simplified to the MI effect between A and M_1:

$$\text{NatINT}_{AM_1} = Y(1, M_1(1), 0) - Y(0, M_1(1), 0) - Y(1, M_1(0), 0) + Y(0, M_1(0), 0),$$

which is illustrated in Figure 4a.

S3 Decomposition of TE in a sequential two-mediator scenario

Suppose we have a directed acyclic graph as shown in Figure 5. We show that the TE can be decomposed into the following ten components at the individual level:

$$\begin{aligned}\text{TE} = {} & \text{CDE}(m_1^*, m_2^*) + \text{INT}_{\text{ref-AM}_1}(m_1^*, m_2^*) + \text{INT}_{\text{ref-AM}_2}(m_1^*, m_2^*) + \text{INT}_{\text{ref-AM}_1M_2}(m_1^*, m_2^*) \\ & + \text{NatINT}_{AM_1} + \text{NatINT}_{AM_2} + \text{NatINT}_{AM_1M_2} + \text{NatINT}_{M_1M_2} + \text{PIE}_{M_1} + \text{SNIE}_{M_2},\end{aligned}$$

where all the natural MI effects are listed in Definition 3. We also give the corresponding interpretation for each component.

Proof. We first decompose the TE into TDE [16], NIE through M_1 (NIE_{M_1}) [3], and seminatural indirect effect through M_2 (SNIE_{M_2}) [19].

$$\begin{aligned}\text{TE} &= Y(a) - Y(a^*) \\ &= Y(a, M_1(a), M_2(a, M_1(a))) - Y(a^*, M_1(a^*), M_2(a^*, M_1(a^*))) \\ &= Y(a, M_1(a), M_2(a, M_1(a))) - Y(a^*, M_1(a), M_2(a, M_1(a))) + Y(a^*, M_1(a), M_2(a, M_1(a))) \\ &\quad - Y(a^*, M_1(a^*), M_2(a, M_1(a^*))) + Y(a^*, M_1(a^*), M_2(a, M_1(a^*))) - Y(a^*, M_1(a^*), M_2(a^*, M_1(a^*))),\end{aligned}$$

where the second equality follows by the composition axiom [8,15] and the third equality follows by adding and subtracting the same identifiable counterfactual formulas. □

The formulas of TDE, NIE_{M_1}, and SNIE_{M_2} are presented as follows:

$$\begin{aligned}\text{TDE} &= Y(a, M_1(a), M_2(a, M_1(a))) - Y(a^*, M_1(a), M_2(a, M_1(a))), \\ \text{NIE}_{M_1} &= Y(a^*, M_1(a), M_2(a, M_1(a))) - Y(a^*, M_1(a^*), M_2(a, M_1(a^*))), \\ \text{SNIE}_{M_2} &= Y(a^*, M_1(a^*), M_2(a, M_1(a^*))) - Y(a^*, M_1(a^*), M_2(a^*, M_1(a^*))),\end{aligned}$$

where $\text{TE} = \text{TDE} + \text{NIE}_{M_1} + \text{SNIE}_{M_2}$.

We next focus on TDE and decompose it into natural MI effects and PDE [16] by subtracting PDE from TDE, where PDE satisfies the definition of a path-specific effect [17] and equals the following difference of two identifiable counterfactual formulas:

$$\text{PDE} = Y(a, M_1(a^*), M_2(a^*, M_1(a^*))) - Y(a^*, M_1(a^*), M_2(a^*, M_1(a^*))).$$

We have the following results:

$$\begin{aligned}
\text{TDE} - \text{PDE} &= Y(a, M_1(a), M_2(a, M_1(a))) - Y(a^*, M_1(a), M_2(a, M_1(a))) \\
&\quad - Y(a, M_1(a^*), M_2(a^*, M_1(a^*))) + Y(a^*, M_1(a^*), M_2(a^*, M_1(a^*))) \\
&= Y(a, M_1(a), M_2(a, M_1(a))) - Y(a^*, M_1(a), M_2(a, M_1(a))) \\
&\quad - Y(a, M_1(a^*), M_2(a^*, M_1(a^*))) + Y(a^*, M_1(a^*), M_2(a^*, M_1(a^*))) \\
&\quad + Y(a^*, M_1(a^*), M_2(a^*, M_1(a^*))) - Y(a^*, M_1(a^*), M_2(a^*, M_1(a^*))) \\
&\quad + Y(a^*, M_1(a^*), M_2(a, M_1(a^*))) - Y(a^*, M_1(a^*), M_2(a, M_1(a^*))) \\
&\quad + Y(a^*, M_1(a), M_2(a^*, M_1(a))) - Y(a^*, M_1(a), M_2(a^*, M_1(a))) \\
&\quad + Y(a, M_1(a^*), M_2(a^*, M_1(a^*))) - Y(a, M_1(a^*), M_2(a^*, M_1(a^*))) \\
&\quad + Y(a, M_1(a^*), M_2(a, M_1(a^*))) - Y(a, M_1(a^*), M_2(a, M_1(a^*))) \\
&\quad + Y(a, M_1(a), M_2(a^*, M_1(a))) - Y(a, M_1(a), M_2(a^*, M_1(a))) \\
&= Y(a, M_1(a), M_2(a^*, M_1(a))) - Y(a^*, M_1(a), M_2(a^*, M_1(a))) \\
&\quad - Y(a, M_1(a^*), M_2(a^*, M_1(a^*))) + Y(a^*, M_1(a^*), M_2(a^*, M_1(a^*))) \\
&\quad + Y(a, M_1(a^*), M_2(a, M_1(a^*))) - Y(a^*, M_1(a^*), M_2(a, M_1(a^*))) \\
&\quad - Y(a, M_1(a^*), M_2(a^*, M_1(a^*))) + Y(a^*, M_1(a^*), M_2(a^*, M_1(a^*))) \\
&\quad + Y(a, M_1(a), M_2(a, M_1(a))) - Y(a^*, M_1(a), M_2(a, M_1(a))) \\
&\quad - Y(a, M_1(a^*), M_2(a, M_1(a^*))) + Y(a^*, M_1(a^*), M_2(a, M_1(a^*))) \\
&\quad - Y(a, M_1(a), M_2(a^*, M_1(a))) + Y(a^*, M_1(a), M_2(a^*, M_1(a))) \\
&\quad + Y(a, M_1(a^*), M_2(a^*, M_1(a^*))) - Y(a^*, M_1(a^*), M_2(a^*, M_1(a^*))).
\end{aligned}$$

where the second equality follows by adding and subtracting the same identifiable counterfactual formulas and the third equality follows by rearranging all the terms to satisfy the definition of the natural MI effects.

Therefore, we have the following formulas satisfying Definition 3:

$$\begin{aligned}
\text{NatINT}_{AM_1} &= Y(a, M_1(a), M_2(a^*, M_1(a))) - Y(a^*, M_1(a), M_2(a^*, M_1(a))) \\
&\quad - Y(a, M_1(a^*), M_2(a^*, M_1(a^*))) + Y(a^*, M_1(a^*), M_2(a^*, M_1(a^*))), \\
\text{NatINT}_{AM_2} &= Y(a, M_1(a^*), M_2(a, M_1(a^*))) - Y(a^*, M_1(a^*), M_2(a, M_1(a^*))) \\
&\quad - Y(a, M_1(a^*), M_2(a^*, M_1(a^*))) + Y(a^*, M_1(a^*), M_2(a^*, M_1(a^*))), \\
\text{NatINT}_{AM_1M_2} &= Y(a, M_1(a), M_2(a, M_1(a))) - Y(a^*, M_1(a), M_2(a, M_1(a))) \\
&\quad - Y(a, M_1(a^*), M_2(a, M_1(a^*))) + Y(a^*, M_1(a^*), M_2(a, M_1(a^*))) \\
&\quad - Y(a, M_1(a), M_2(a^*, M_1(a))) + Y(a^*, M_1(a), M_2(a^*, M_1(a))) \\
&\quad + Y(a, M_1(a^*), M_2(a^*, M_1(a^*))) - Y(a^*, M_1(a^*), M_2(a^*, M_1(a^*))).
\end{aligned}$$

Accordingly, TDE can be decomposed into the following components:

$$\text{TDE} = \text{PDE} + \text{NatINT}_{AM_1} + \text{NatINT}_{AM_2} + \text{NatINT}_{AM_1M_2}.$$

We next focus on PDE (path-specific effect) and decompose it into CDE and reference interaction effects [7,9]:

$$\begin{aligned}
\text{PDE} &= Y(a, M_1(a^*), M_2(a^*, M_1(a^*))) - Y(a^*, M_1(a^*), M_2(a^*, M_1(a^*))) \\
&= \sum_{m_2}\sum_{m_1} Y(a, m_1, m_2) \times I(M_1(a^*) = m_1) \times I(M_2(a^*, m_1) = m_2) \\
&\quad - \sum_{m_2}\sum_{m_1} Y(a^*, m_1, m_2) \times I(M_1(a^*) = m_1) \times I(M_2(a^*, m_1) = m_2) \\
&= \sum_{m_2}\sum_{m_1} [Y(a, m_1, m_2) - Y(a^*, m_1, m_2)] \times I(M_1(a^*) = m_1) \times I(M_2(a^*, m_1) = m_2) \\
&= \sum_{m_2}\sum_{m_1} [Y(a, m_1, m_2) - Y(a^*, m_1, m_2) - Y(a, m_1^*, m_2^*) + Y(a^*, m_1^*, m_2^*)] \times I(M_1(a^*) = m_1) \\
&\quad \times I(M_2(a^*, m_1) = m_2) + \sum_{m_2}\sum_{m_1} [Y(a, m_1^*, m_2^*) - Y(a^*, m_1^*, m_2^*)] \times I(M_1(a^*) = m_1) \times I(M_2(a^*, m_1) = m_2) \\
&= \sum_{m_2}\sum_{m_1} [Y(a, m_1, m_2) - Y(a^*, m_1, m_2) - Y(a, m_1^*, m_2^*) + Y(a^*, m_1^*, m_2^*)] \times I(M_1(a^*) = m_1) \\
&\quad \times I(M_2(a^*, m_1) = m_2) + Y(a, m_1^*, m_2^*) - Y(a^*, m_1^*, m_2^*)
\end{aligned}$$

$$\begin{aligned}\mathrm{PDE} = &\sum_{m_2}\sum_{m_1}[Y(a, m_1, m_2) - Y(a^*, m_1, m_2) - Y(a, m_1^*, m_2^*) + Y(a^*, m_1^*, m_2^*) + Y(a^*, m_1, m_2^*) - Y(a^*, m_1, m_2^*)\\
&+ Y(a, m_1, m_2^*) - Y(a, m_1, m_2^*)] \times I(M_1(a^*) = m_1) \times I(M_2(a^*, m_1) = m_2) + Y(a, m_1^*, m_2^*)\\
&- Y(a^*, m_1^*, m_2^*)\\
= &\sum_{m_2}\sum_{m_1}[Y(a, m_1, m_2^*) - Y(a^*, m_1, m_2^*) - Y(a, m_1^*, m_2^*) + Y(a^*, m_1^*, m_2^*)] \times I(M_1(a^*) = m_1)\\
&\times I(M_2(a^*, m_1) = m_2) + \sum_{m_2}\sum_{m_1}[Y(a, m_1, m_2) - Y(a, m_1, m_2^*) - Y(a^*, m_1, m_2)\\
&+ Y(a^*, m_1, m_2^*)] \times I(M_1(a^*) = m_1) \times I(M_2(a^*, m_1) = m_2) + Y(a, m_1^*, m_2^*) - Y(a^*, m_1^*, m_2^*)\\
= &\sum_{m_1}[Y(a, m_1, m_2^*) - Y(a^*, m_1, m_2^*) - Y(a, m_1^*, m_2^*) + Y(a^*, m_1^*, m_2^*)] \times I(M_1(a^*) = m_1)\\
&+ \sum_{m_2}\sum_{m_1}[Y(a, m_1, m_2) - Y(a, m_1, m_2^*) - Y(a^*, m_1, m_2) + Y(a^*, m_1, m_2^*)] \times I(M_1(a^*) = m_1)\\
&\times I(M_2(a^*, m_1) = m_2) + Y(a, m_1^*, m_2^*) - Y(a^*, m_1^*, m_2^*).\end{aligned}$$

According to the derivation above, the following formulas can be obtained:

$$\begin{aligned}\mathrm{CDE}(m_1^*, m_2^*) &= Y(a, m_1^*, m_2^*) - Y(a^*, m_1^*, m_2^*),\\
\mathrm{INT}_{\text{ref-AM}_1}(m_1^*, m_2^*) &= \sum_{m_1}[Y(a, m_1, m_2^*) - Y(a^*, m_1, m_2^*) - Y(a, m_1^*, m_2^*) + Y(a^*, m_1^*, m_2^*)] \times I(M_1(a^*) = m_1),\\
\mathrm{INT}_{\text{ref-AM}_2+AM_1M_2}(m_2^*) &= \sum_{m_2}\sum_{m_1}[Y(a, m_1, m_2) - Y(a, m_1, m_2^*) - Y(a^*, m_1, m_2) + Y(a^*, m_1, m_2^*)]\\
&\quad \times I(M_1(a^*) = m_1) \times I(M_2(a^*, m_1) = m_2).\end{aligned}$$

With a little mathematical derivation, $\mathrm{INT}_{\text{ref-AM}_1}$ and $\mathrm{INT}_{\text{ref-AM}_2+AM_1M_2}$ can be expressed in the form of the counterfactual formulas:

$$\begin{aligned}\mathrm{INT}_{\text{ref-AM}_1}(m_1^*, m_2^*) &= Y(a, M_1(a^*), m_2^*) - Y(a^*, M_1(a^*), m_2^*) - Y(a, m_1^*, m_2^*) + Y(a^*, m_1^*, m_2^*),\\
\mathrm{INT}_{\text{ref-AM}_2+AM_1M_2}(m_2^*) &= Y(a, M_1(a^*), M_2(a^*, M_1(a^*))) - Y(a, M_1(a^*), m_2^*)\\
&\quad - Y(a^*, M_1(a^*), M_2(a^*, M_1(a^*))) + Y(a^*, M_1(a^*), m_2^*).\end{aligned}$$

It is worth noting that it is very likely to encounter a variant of the problematic counterfactual formulas proposed by Avin et al. [15] when we try to separate $\mathrm{INT}_{\text{ref-AM}_2+AM_1M_2}(m_2^*)$ into $\mathrm{INT}_{\text{ref-AM}_2}(m_1^*, m_2^*)$ and $\mathrm{INT}_{\text{ref-AM}_1M_2}(m_1^*, m_2^*)$. The details of the issue are discussed in Supplementary material S4. We next present the process that separates $\mathrm{INT}_{\text{ref-AM}_2+AM_1M_2}(m_2^*)$ and ensures the identifiability in the mean time.

$$\begin{aligned}
&\mathrm{INT}_{\text{ref-}AM_2+AM_1M_2}(m_2^*)\\
&= \sum_{m_2}\sum_{m_1}[Y(a, m_1, m_2) - Y(a, m_1, m_2^*) - Y(a^*, m_1, m_2) + Y(a^*, m_1, m_2^*)] \times I(M_1(a^*) = m_1)\\
&\quad \times I(M_2(a^*, m_1) = m_2)\\
&= \sum_{m_2}\sum_{m_1}[Y(a, m_1, m_2) - Y(a, m_1, m_2^*) - Y(a^*, m_1, m_2) + Y(a^*, m_1, m_2^*) + Y(a^*, m_1^*, m_2^*)\\
&\quad - Y(a^*, m_1^*, m_2^*) + Y(a, m_1^*, m_2^*) - Y(a, m_1^*, m_2^*)] \times I(M_1(a^*) = m_1) \times I(M_2(a^*, m_1) = m_2)\\
&= \sum_{m_2}\sum_{m_1}[Y(a, m_1, m_2) - Y(a, m_1, m_2^*) - Y(a^*, m_1, m_2) + Y(a^*, m_1, m_2^*) + Y(a^*, m_1^*, m_2^*)\\
&\quad - Y(a^*, m_1^*, m_2^*) + Y(a, m_1^*, m_2^*) - Y(a, m_1^*, m_2^*)] \times I(M_1(a^*) = m_1) \times I(M_2(a^*, m_1) = m_2)\\
&\quad + \sum_{m_2}\sum_{m_1}[Y(a, m_1^*, m_2) - Y(a, m_1^*, m_2) + Y(a^*, m_1^*, m_2) - Y(a^*, m_1^*, m_2)] \times I(M_1(a^*) = m_1)\\
&\quad \times I(M_2(a^*, m_1^*) = m_2)\\
&= \sum_{m_2}\sum_{m_1}[Y(a, m_1^*, m_2) - Y(a^*, m_1^*, m_2)] \times I(M_1(a^*) = m_1) \times I(M_2(a^*, m_1^*) = m_2)\\
&\quad + \sum_{m_2}\sum_{m_1}[-Y(a, m_1^*, m_2^*) + Y(a^*, m_1^*, m_2^*)] \times I(M_1(a^*) = m_1) \times I(M_2(a^*, m_1) = m_2)\\
&\quad + \sum_{m_2}\sum_{m_1}[Y(a, m_1, m_2) - Y(a^*, m_1, m_2) - Y(a, m_1, m_2^*) + Y(a^*, m_1, m_2^*) + Y(a, m_1^*, m_2^*)\\
&\quad - Y(a^*, m_1^*, m_2^*)] \times I(M_1(a^*) = m_1) \times I(M_2(a^*, m_1) = m_2)\\
&\quad + \sum_{m_2}\sum_{m_1}[-Y(a, m_1^*, m_2) + Y(a^*, m_1^*, m_2)] \times I(M_1(a^*) = m_1) \times I(M_2(a^*, m_1^*) = m_2).
\end{aligned}$$

According to the derivation above, the following formulas can be obtained:

$$\begin{aligned}
\mathrm{INT}_{\text{ref-}AM_2}(m_1^*, m_2^*) &= \sum_{m_2}\sum_{m_1}[Y(a, m_1^*, m_2) - Y(a^*, m_1^*, m_2)] \times I(M_1(a^*) = m_1) \times I(M_2(a^*, m_1^*) = m_2)\\
&\quad + \sum_{m_2}\sum_{m_1}[-Y(a, m_1^*, m_2^*) + Y(a^*, m_1^*, m_2^*)] \times I(M_1(a^*) = m_1) \times I(M_2(a^*, m_1) = m_2),\\
\mathrm{INT}_{\text{ref-}AM_1M_2}(m_1^*, m_2^*) &= \sum_{m_2}\sum_{m_1}[Y(a, m_1, m_2) - Y(a^*, m_1, m_2) - Y(a, m_1, m_2^*) + Y(a^*, m_1, m_2^*) + Y(a, m_1^*, m_2^*)\\
&\quad - Y(a^*, m_1^*, m_2^*)] \times I(M_1(a^*) = m_1) \times I(M_2(a^*, m_1) = m_2)\\
&\quad + \sum_{m_2}\sum_{m_1}[-Y(a, m_1^*, m_2) + Y(a^*, m_1^*, m_2)] \times I(M_1(a^*) = m_1) \times I(M_2(a^*, m_1^*) = m_2).
\end{aligned}$$

$\mathrm{INT}_{\text{ref-}AM_2}$ and $\mathrm{INT}_{\text{ref-}AM_1M_2}$ can also be expressed in the form of the counterfactual formulas:

$$\begin{aligned}
\mathrm{INT}_{\text{ref-}AM_2}(m_1^*, m_2^*) &= Y(a, m_1^*, M_2(a^*, m_1^*)) - Y(a^*, m_1^*, M_2(a^*, m_1^*)) - Y(a, m_1^*, m_2^*) + Y(a^*, m_1^*, m_2^*),\\
\mathrm{INT}_{\text{ref-}AM_1M_2}(m_1^*, m_2^*) &= Y(a, M_1(a^*), M_2(a^*, M_1(a^*))) - Y(a^*, M_1(a^*), M_2(a^*, M_1(a^*)))\\
&\quad - Y(a, m_1^*, M_2(a^*, m_1^*)) + Y(a^*, m_1^*, M_2(a^*, m_1^*)) - Y(a, M_1(a^*), m_2^*) + Y(a^*, M_1(a^*), m_2^*)\\
&\quad + Y(a, m_1^*, m_2^*) - Y(a^*, m_1^*, m_2^*).
\end{aligned}$$

Therefore, PDE can be decomposed into the following components:

$$\mathrm{PDE} = \mathrm{CDE}(m_1^*, m_2^*) + \mathrm{INT}_{\text{ref-}AM_1}(m_1^*, m_2^*) + \mathrm{INT}_{\text{ref-}AM_2}(m_1^*, m_2^*) + \mathrm{INT}_{\text{ref-}AM_1M_2}(m_1^*, m_2^*).$$

TDE can be decomposed into the following components:

$$\begin{aligned}
\mathrm{TDE} &= \mathrm{PDE} + \mathrm{NatINT}_{AM_1} + \mathrm{NatINT}_{AM_2} + \mathrm{NatINT}_{AM_1M_2}\\
&= \mathrm{CDE}(m_1^*, m_2^*) + \mathrm{INT}_{\text{ref-}AM_1}(m_1^*, m_2^*) + \mathrm{INT}_{\text{ref-}AM_2}(m_1^*, m_2^*) + \mathrm{INT}_{\text{ref-}AM_1M_2}(m_1^*, m_2^*)\\
&\quad + \mathrm{NatINT}_{AM_1} + \mathrm{NatINT}_{AM_2} + \mathrm{NatINT}_{AM_1M_2}.
\end{aligned}$$

We next focus on NIE_{M_1} and decompose it into PIE_{M_1} and $\mathrm{NatINT}_{M_1M_2}$ by subtracting PIE_{M_1} from NIE_{M_1}:

$$\begin{aligned}\mathrm{NIE}_{M_1} - \mathrm{PIE}_{M_1} &= Y(a^*, M_1(a), M_2(a, M_1(a))) - Y(a^*, M_1(a^*), M_2(a, M_1(a^*)))\\ &\quad - Y(a^*, M_1(a), M_2(a^*, M_1(a))) + Y(a^*, M_1(a^*), M_2(a^*, M_1(a^*)))\\ &= \mathrm{NatINT}_{M_1M_2},\end{aligned}$$

where $\mathrm{NatINT}_{M_1M_2}$ is listed in Definition 3 and PIE_{M_1} satisfies the definition of a path-specific effect [17].

Therefore, NIE_{M_1} can be decomposed into the following components:

$$\mathrm{NIE}_{M_1} = \mathrm{PIE}_{M_1} + \mathrm{NatINT}_{M_1M_2}.$$

Combining all the derivations above, we have the decomposition of TE as follows:

$$\begin{aligned}\mathrm{TE} = &\ \mathrm{CDE}(m_1^*, m_2^*) + \mathrm{INT}_{\text{ref-}AM_1}(m_1^*, m_2^*) + \mathrm{INT}_{\text{ref-}AM_2}(m_1^*, m_2^*) + \mathrm{INT}_{\text{ref-}AM_1M_2}(m_1^*, m_2^*)\\ &+ \mathrm{NatINT}_{AM_1} + \mathrm{NatINT}_{AM_2} + \mathrm{NatINT}_{AM_1M_2} + \mathrm{NatINT}_{M_1M_2} + \mathrm{PIE}_{M_1} + \mathrm{SNIE}_{M_2}.\end{aligned}$$

We next present the interpretation for each component assuming binary A, M_1, and M_2 with the conditions $a = 1$, $a^* = 0$, $m_1^* = 0$, and $m_2^* = 0$ for illustration purpose. While other interpretations were proposed in the literature [7,9], our work represents a different and more flexible interpretation from the perspective of population averages which accounts for the distribution of the mediators in the causal structure.

S3.1 Controlled direct effect

With the specified conditions, the CDE can be written as:

$$\mathrm{CDE}(m_1^*, m_2^*) = Y(a, m_1^*, m_2^*) - Y(a^*, m_1^*, m_2^*) \quad \Rightarrow \quad \mathrm{CDE}(0, 0) = Y(1, 0, 0) - Y(0, 0, 0).$$

$\mathrm{CDE}(m_1^*, m_2^*)$ can be interpreted as the effect due to neither mediation nor interaction.

S3.2 Reference interaction effects

With the specified conditions, the reference interaction effect between A and M_1 can be written as:

$$\begin{aligned}\mathrm{INT}_{\text{ref-}AM_1}(m_1^*, m_2^*) &= \sum_{m_1}[Y(a, m_1, m_2^*) - Y(a^*, m_1, m_2^*) - Y(a, m_1^*, m_2^*) + Y(a^*, m_1^*, m_2^*)] \times I(M_1(a^*) = m_1)\\ \Rightarrow \ \mathrm{INT}_{\text{ref-}AM_1}(0, 0) &= \sum_{m_1}[Y(1, m_1, 0) - Y(0, m_1, 0) - Y(1, 0, 0) + Y(0, 0, 0)] \times I(M_1(0) = m_1)\\ &= [Y(1, 0, 0) - Y(0, 0, 0) - Y(1, 0, 0) + Y(0, 0, 0)] \times I(M_1(0) = 0)\\ &\quad + [Y(1, 1, 0) - Y(0, 1, 0) - Y(1, 0, 0) + Y(0, 0, 0)] \times I(M_1(0) = 1)\\ &= [Y(1, 1, 0) - Y(0, 1, 0) - Y(1, 0, 0) + Y(0, 0, 0)] \times I(M_1(0) = 1)\\ &= [Y(1, 1, 0) - Y(0, 1, 0) - Y(1, 0, 0) + Y(0, 0, 0)] \times M_1(0).\end{aligned}$$

$\mathrm{INT}_{\text{ref-}AM_1}(m_1^*, m_2^*)$ can be interpreted as the effect due to the interaction between A and M_1 only.

With the specified conditions, the reference interaction effect between A and M_2 can be written as:

$$
\begin{aligned}
&\mathrm{INT}_{\text{ref-}AM_2}(m_1^*, m_2^*)\\
&= \sum_{m_2}\sum_{m_1}[Y(a, m_1^*, m_2) - Y(a^*, m_1^*, m_2)] \times I(M_1(a^*) = m_1) \times I(M_2(a^*, m_1^*) = m_2)\\
&\quad + \sum_{m_2}\sum_{m_1}[-Y(a, m_1^*, m_2^*) + Y(a^*, m_1^*, m_2^*)] \times I(M_1(a^*) = m_1) \times I(M_2(a^*, m_1) = m_2)\\
\Rightarrow\ &\mathrm{INT}_{\text{ref-}AM_2}(0, 0)\\
&= \sum_{m_2}\sum_{m_1}[Y(1, 0, m_2) - Y(0, 0, m_2)] \times I(M_1(0) = m_1) \times I(M_2(0, 0) = m_2)\\
&\quad + \sum_{m_2}\sum_{m_1}[-Y(1, 0, 0) + Y(0, 0, 0)] \times I(M_1(0) = m_1) \times I(M_2(0, m_1) = m_2)\\
&= \sum_{m_2}[Y(1, 0, m_2) - Y(0, 0, m_2)] \times I(M_1(0) = 0) \times I(M_2(0, 0) = m_2)\\
&\quad + \sum_{m_2}[Y(1, 0, m_2) - Y(0, 0, m_2)] \times I(M_1(0) = 1) \times I(M_2(0, 0) = m_2)\\
&\quad + \sum_{m_2}[-Y(1, 0, 0) + Y(0, 0, 0)] \times I(M_1(0) = 0) \times I(M_2(0, 0) = m_2)\\
&\quad + \sum_{m_2}[-Y(1, 0, 0) + Y(0, 0, 0)] \times I(M_1(0) = 1) \times I(M_2(0, 1) = m_2)\\
&= [Y(1, 0, 0) - Y(0, 0, 0)] \times I(M_1(0) = 0) \times I(M_2(0, 0) = 0) + [Y(1, 0, 1) - Y(0, 0, 1)] \times I(M_1(0) = 0)\\
&\quad \times I(M_2(0, 0) = 1) + [Y(1, 0, 0) - Y(0, 0, 0)] \times I(M_1(0) = 1) \times I(M_2(0, 0) = 0)\\
&\quad + [Y(1, 0, 1) - Y(0, 0, 1)] \times I(M_1(0) = 1) \times I(M_2(0, 0) = 1) + [-Y(1, 0, 0) + Y(0, 0, 0)]\\
&\quad \times I(M_1(0) = 0) \times I(M_2(0, 0) = 0) + [-Y(1, 0, 0) + Y(0, 0, 0)] \times I(M_1(0) = 0) \times I(M_2(0, 0) = 1)\\
&\quad + [-Y(1, 0, 0) + Y(0, 0, 0)] \times I(M_1(0) = 1) \times I(M_2(0, 1) = 0) + [-Y(1, 0, 0) + Y(0, 0, 0)]\\
&\quad \times I(M_1(0) = 1) \times I(M_2(0, 1) = 1)\\
&= [Y(1, 0, 1) - Y(0, 0, 1) - Y(1, 0, 0) + Y(0, 0, 0)] \times I(M_1(0) = 0) \times I(M_2(0, 0) = 1)\\
&\quad + \{[Y(1, 0, 0) - Y(0, 0, 0)] \times I(M_2(0, 0) = 0) + [Y(1, 0, 1) - Y(0, 0, 1)] \times I(M_2(0, 0) = 1)\\
&\quad + [-Y(1, 0, 0) + Y(0, 0, 0)] \times I(M_2(0, 1) = 0) + [-Y(1, 0, 0) + Y(0, 0, 0)] \times I(M_2(0, 1) = 1)\}\\
&\quad \times I(M_1(0) = 1)\\
&= [Y(1, 0, 1) - Y(0, 0, 1) - Y(1, 0, 0) + Y(0, 0, 0)] \times I(M_1(0) = 0) \times I(M_2(0, 0) = 1)\\
&\quad + \{[Y(1, 0, 0) - Y(0, 0, 0)] \times [1 - I(M_2(0, 0) = 1)] + [Y(1, 0, 1) - Y(0, 0, 1)] \times I(M_2(0, 0) = 1)\\
&\quad + [-Y(1, 0, 0) + Y(0, 0, 0)] \times [1 - I(M_2(0, 1) = 1)] + [-Y(1, 0, 0) + Y(0, 0, 0)] \times I(M_2(0, 1) = 1)\}\\
&\quad \times I(M_1(0) = 1)\\
&= [Y(1, 0, 1) - Y(0, 0, 1) - Y(1, 0, 0) + Y(0, 0, 0)] \times I(M_1(0) = 0) \times I(M_2(0, 0) = 1)\\
&\quad + \{-[Y(1, 0, 0) - Y(0, 0, 0)] \times I(M_2(0, 0) = 1) + [Y(1, 0, 1) - Y(0, 0, 1)] \times I(M_2(0, 0) = 1)\}\\
&\quad \times I(M_1(0) = 1)\\
&= [Y(1, 0, 1) - Y(0, 0, 1) - Y(1, 0, 0) + Y(0, 0, 0)] \times I(M_1(0) = 0) \times I(M_2(0, 0) = 1)\\
&\quad + [Y(1, 0, 1) - Y(0, 0, 1) - Y(1, 0, 0) + Y(0, 0, 0)] \times I(M_1(0) = 1) \times I(M_2(0, 0) = 1)\\
&= [Y(1, 0, 1) - Y(0, 0, 1) - Y(1, 0, 0) + Y(0, 0, 0)] \times [1 - I(M_1(0) = 1)] \times I(M_2(0, 0) = 1)\\
&\quad + [Y(1, 0, 1) - Y(0, 0, 1) - Y(1, 0, 0) + Y(0, 0, 0)] \times I(M_1(0) = 1) \times I(M_2(0, 0) = 1)\\
&= [Y(1, 0, 1) - Y(0, 0, 1) - Y(1, 0, 0) + Y(0, 0, 0)] \times I(M_2(0, 0) = 1)\\
&= [Y(1, 0, 1) - Y(0, 0, 1) - Y(1, 0, 0) + Y(0, 0, 0)] \times M_2(0, 0),
\end{aligned}
$$

where the fifth equality follows by the fact that $I(M_2(0, 0) = 0) = 1 - I(M_2(0, 0) = 1)$ and $I(M_2(0, 1) = 0) = 1 - I(M_2(0, 1) = 1)$, the eighth equality follows by the fact that $I(M_1(0) = 0) = 1 - I(M_1(0) = 1)$ and the last equality follows by the fact that $I(M_2(0, 0) = 1) = M_2(0, 0)$.

$\mathrm{INT}_{\text{ref-}AM_2}(m_1^*, m_2^*)$ can be interpreted as the effect due to the interaction between A and M_2 only.

With the specified conditions, the reference interaction effect between A, M_1, and M_2 can be written as:

$$
\begin{aligned}
\mathrm{INT}_{\text{ref-}AM_1M_2}(m_1^*, m_2^*) &= \sum_{m_2}\sum_{m_1}[Y(a, m_1, m_2) - Y(a^*, m_1, m_2) - Y(a, m_1, m_2^*) + Y(a^*, m_1, m_2^*) \\
&\quad + Y(a, m_1^*, m_2^*) - Y(a^*, m_1^*, m_2^*)] \times I(M_1(a^*) = m_1) \times I(M_2(a^*, m_1) = m_2) \\
&\quad + \sum_{m_2}\sum_{m_1}[-Y(a, m_1^*, m_2) + Y(a^*, m_1^*, m_2)] \times I(M_1(a^*) = m_1) \times I(M_2(a^*, m_1^*) = m_2) \\
\Rightarrow \mathrm{INT}_{\text{ref-}AM_1M_2}(0, 0) &= \sum_{m_2}\sum_{m_1}[Y(1, m_1, m_2) - Y(0, m_1, m_2) - Y(1, m_1, 0) + Y(0, m_1, 0) + Y(1, 0, 0) - Y(0, 0, 0)] \\
&\quad \times I(M_1(0) = m_1) \times I(M_2(0, m_1) = m_2) \\
&\quad + \sum_{m_2}\sum_{m_1}[-Y(1, 0, m_2) + Y(0, 0, m_2)] \times I(M_1(0) = m_1) \times I(M_2(0, 0) = m_2) \\
&= \sum_{m_2}[Y(1, 0, m_2) - Y(0, 0, m_2) - Y(1, 0, 0) + Y(0, 0, 0) + Y(1, 0, 0) - Y(0, 0, 0)] \\
&\quad \times I(M_1(0) = 0) \times I(M_2(0, 0) = m_2) + \sum_{m_2}[Y(1, 1, m_2) - Y(0, 1, m_2) \\
&\quad - Y(1, 1, 0) + Y(0, 1, 0) + Y(1, 0, 0) - Y(0, 0, 0)] \times I(M_1(0) = 1) \times I(M_2(0, 1) = m_2) \\
&\quad + \sum_{m_2}[-Y(1, 0, m_2) + Y(0, 0, m_2)] \times I(M_1(0) = 0) \times I(M_2(0, 0) = m_2) \\
&\quad + \sum_{m_2}[-Y(1, 0, m_2) + Y(0, 0, m_2)] \times I(M_1(0) = 1) \times I(M_2(0, 0) = m_2) \\
&= [Y(1, 0, 0) - Y(0, 0, 0) - Y(1, 0, 0) + Y(0, 0, 0) + Y(1, 0, 0) - Y(0, 0, 0)] \\
&\quad \times I(M_1(0) = 0) \times I(M_2(0, 0) = 0) + [Y(1, 0, 1) - Y(0, 0, 1) - Y(1, 0, 0) \\
&\quad + Y(0, 0, 0) + Y(1, 0, 0) - Y(0, 0, 0)] \times I(M_1(0) = 0) \times I(M_2(0, 0) = 1) \\
&\quad + [Y(1, 1, 0) - Y(0, 1, 0) - Y(1, 1, 0) + Y(0, 1, 0) + Y(1, 0, 0) - Y(0, 0, 0)] \\
&\quad \times I(M_1(0) = 1) \times I(M_2(0, 1) = 0) + [Y(1, 1, 1) - Y(0, 1, 1) - Y(1, 1, 0) \\
&\quad + Y(0, 1, 0) + Y(1, 0, 0) - Y(0, 0, 0)] \times I(M_1(0) = 1) \times I(M_2(0, 1) = 1) \\
&\quad + [-Y(1, 0, 0) + Y(0, 0, 0)] \times I(M_1(0) = 0) \times I(M_2(0, 0) = 0) \\
&\quad + [-Y(1, 0, 1) + Y(0, 0, 1)] \times I(M_1(0) = 0) \times I(M_2(0, 0) = 1) \\
&\quad + [-Y(1, 0, 0) + Y(0, 0, 0)] \times I(M_1(0) = 1) \times I(M_2(0, 0) = 0) \\
&\quad + [-Y(1, 0, 1) + Y(0, 0, 1)] \times I(M_1(0) = 1) \times I(M_2(0, 0) = 1) \\
&= [Y(1, 0, 0) - Y(0, 0, 0)] \times I(M_1(0) = 1) \times I(M_2(0, 1) = 0) \\
&\quad + [Y(1, 1, 1) - Y(0, 1, 1) - Y(1, 1, 0) + Y(0, 1, 0) + Y(1, 0, 0) - Y(0, 0, 0)] \times I(M_1(0) = 1) \\
&\quad \times I(M_2(0, 1) = 1) + [-Y(1, 0, 0) + Y(0, 0, 0)] \times I(M_1(0) = 1) \times I(M_2(0, 0) = 0) \\
&\quad + [-Y(1, 0, 1) + Y(0, 0, 1)] \times I(M_1(0) = 1) \times I(M_2(0, 0) = 1) \\
&= [Y(1, 0, 0) - Y(0, 0, 0)] \times I(M_1(0) = 1) \times [1 - I(M_2(0, 1) = 1)] \\
&\quad + [Y(1, 1, 1) - Y(0, 1, 1) - Y(1, 1, 0) + Y(0, 1, 0) + Y(1, 0, 0) - Y(0, 0, 0)] \\
&\quad \times I(M_1(0) = 1) \times I(M_2(0, 1) = 1) + [-Y(1, 0, 0) + Y(0, 0, 0)] \times I(M_1(0) = 1) \\
&\quad \times [1 - I(M_2(0, 0) = 1)] + [-Y(1, 0, 1) + Y(0, 0, 1)] \times I(M_1(0) = 1) \times I(M_2(0, 0) = 1) \\
&= [Y(1, 1, 1) - Y(0, 1, 1) - Y(1, 1, 0) + Y(0, 1, 0)] \times I(M_1(0) = 1) \times I(M_2(0, 1) = 1) \\
&\quad + [-Y(1, 0, 1) + Y(0, 0, 1) + Y(1, 0, 0) - Y(0, 0, 0)] \times I(M_1(0) = 1) \times I(M_2(0, 0) = 1) \\
&= [Y(1, 1, 1) - Y(0, 1, 1) - Y(1, 1, 0) + Y(0, 1, 0)] \times M_1(0) \times M_2(0, 1) \\
&\quad + [-Y(1, 0, 1) + Y(0, 0, 1) + Y(1, 0, 0) - Y(0, 0, 0)] \times M_1(0) \times M_2(0, 0),
\end{aligned}
$$

where the fifth equality follows by the fact that $I(M_2(0, 0) = 0) = 1 - I(M_2(0, 0) = 1)$ and $I(M_2(0, 1) = 0) = 1 - I(M_2(0, 1) = 1)$, and the last equality follows by the fact that $I(M_1(0) = 1) = M_1(0)$, $I(M_2(0, 1) = 1) = M_2(0, 1)$ and $I(M_2(0, 0) = 1) = M_2(0, 0)$.

$\mathrm{INT}_{\text{ref-}AM_1M_2}(m_1^*, m_2^*)$ can be interpreted as the effect due to the interaction between A, M_1, and M_2 only.

S3.3 Natural MI effects

The natural MI effect between A and M_1 can be rewritten as:

$$\begin{aligned}
\text{NatINT}_{AM_1} &= Y(a, M_1(a), M_2(a^*, M_1(a))) - Y(a^*, M_1(a), M_2(a^*, M_1(a)))\\
&\quad - Y(a, M_1(a^*), M_2(a^*, M_1(a^*))) + Y(a^*, M_1(a^*), M_2(a^*, M_1(a^*)))\\
&= \sum_{m_2}\sum_{m_1} Y(a, m_1, m_2) I(M_1(a) = m_1) I(M_2(a^*, m_1) = m_2)\\
&\quad - \sum_{m_2}\sum_{m_1} Y(a^*, m_1, m_2) I(M_1(a) = m_1) I(M_2(a^*, m_1) = m_2)\\
&\quad - \sum_{m_2}\sum_{m_1} Y(a, m_1, m_2) I(M_1(a^*) = m_1) I(M_2(a^*, m_1) = m_2)\\
&\quad + \sum_{m_2}\sum_{m_1} Y(a^*, m_1, m_2) I(M_1(a^*) = m_1) I(M_2(a^*, m_1) = m_2)\\
&= \sum_{m_2}\sum_{m_1} [Y(a, m_1, m_2) - Y(a^*, m_1, m_2)] I(M_1(a) = m_1) I(M_2(a^*, m_1) = m_2)\\
&\quad - \sum_{m_2}\sum_{m_1} [Y(a, m_1, m_2) - Y(a^*, m_1, m_2)] I(M_1(a^*) = m_1) I(M_2(a^*, m_1) = m_2)\\
&= \sum_{m_2}\sum_{m_1} [Y(a, m_1, m_2) - Y(a^*, m_1, m_2)] \times [I(M_1(a) = m_1) - I(M_1(a^*) = m_1)]\\
&\quad \times I(M_2(a^*, m_1) = m_2)\\
&= \sum_{m_2}\sum_{m_1} [Y(a, m_1, m_2) I(M_2(a^*, m_1) = m_2) - Y(a^*, m_1, m_2) I(M_2(a^*, m_1) = m_2)]\\
&\quad \times [I(M_1(a) = m_1) - I(M_1(a^*) = m_1)]\\
&= \sum_{m_2}\sum_{m_1} [Y(a, m_1, m_2) I(M_2(a^*, m_1) = m_2) - Y(a^*, m_1, m_2) I(M_2(a^*, m_1) = m_2)\\
&\quad - Y(a, m_1^*, m_2) I(M_2(a^*, m_1^*) = m_2) + Y(a^*, m_1^*, m_2) I(M_2(a^*, m_1^*) = m_2)]\\
&\quad \times [I(M_1(a) = m_1) - I(M_1(a^*) = m_1)],
\end{aligned}$$

where the last equation follows by adding two extra terms which do not change the value of NatINT_{AM_1}.

With the specified conditions, NatINT_{AM_1} can be written as:

$$\begin{aligned}
\text{NatINT}_{AM_1} &= \sum_{m_2}\sum_{m_1} [Y(1, m_1, m_2) I(M_2(0, m_1) = m_2) - Y(0, m_1, m_2) I(M_2(0, m_1) = m_2)\\
&\quad - Y(1, 0, m_2) I(M_2(0, 0) = m_2) + Y(0, 0, m_2) I(M_2(0, 0) = m_2)] \times [I(M_1(1) = m_1) - I(M_1(0) = m_1)]\\
&= \sum_{m_2} [Y(1, 0, m_2) I(M_2(0, 0) = m_2) - Y(0, 0, m_2) I(M_2(0, 0) = m_2) - Y(1, 0, m_2) I(M_2(0, 0) = m_2)\\
&\quad + Y(0, 0, m_2) I(M_2(0, 0) = m_2)] \times [I(M_1(1) = 0) - I(M_1(0) = 0)]\\
&\quad + \sum_{m_2} [Y(1, 1, m_2) I(M_2(0, 1) = m_2) - Y(0, 1, m_2) I(M_2(0, 1) = m_2) - Y(1, 0, m_2) I(M_2(0, 0) = m_2)\\
&\quad + Y(0, 0, m_2) I(M_2(0, 0) = m_2)] \times [I(M_1(1) = 1) - I(M_1(0) = 1)]\\
&= \sum_{m_2} [Y(1, 1, m_2) I(M_2(0, 1) = m_2) - Y(0, 1, m_2) I(M_2(0, 1) = m_2) - Y(1, 0, m_2) I(M_2(0, 0) = m_2)\\
&\quad + Y(0, 0, m_2) I(M_2(0, 0) = m_2)] \times [I(M_1(1) = 1) - I(M_1(0) = 1)]\\
&= \sum_{m_2} [Y(1, 1, m_2) I(M_2(0, 1) = m_2) - Y(0, 1, m_2) I(M_2(0, 1) = m_2) - Y(1, 0, m_2) I(M_2(0, 0) = m_2)\\
&\quad + Y(0, 0, m_2) I(M_2(0, 0) = m_2)] \times [M_1(1) - M_1(0)],
\end{aligned}$$

where the indicator functions $I(M_2(0, 1) = m_2)$ and $I(M_2(0, 0) = m_2)$ indicate that M_2 is at its potential values $M_2(0, 1)$ and $M_2(0, 0)$, which may vary with respect to different individuals.

NatINT_{AM_1} can be interpreted as the effect due to the mediation through M_1 and the interaction between A and M_1 conditioning on the potential values of M_2 with the fixed reference level a^*.

The natural MI effect between A and M_2 can be rewritten as:

$$\begin{aligned}
\text{NatINT}_{AM_2} &= Y(a, M_1(a^*), M_2(a, M_1(a^*))) - Y(a^*, M_1(a^*), M_2(a, M_1(a^*)))\\
&\quad - Y(a, M_1(a^*), M_2(a^*, M_1(a^*))) + Y(a^*, M_1(a^*), M_2(a^*, M_1(a^*)))\\
&= \sum_{m_2}\sum_{m_1} Y(a, m_1, m_2) I(M_1(a^*) = m_1) I(M_2(a, m_1) = m_2)\\
&\quad - \sum_{m_2}\sum_{m_1} Y(a^*, m_1, m_2) I(M_1(a^*) = m_1) I(M_2(a, m_1) = m_2)\\
&\quad - \sum_{m_2}\sum_{m_1} Y(a, m_1, m_2) I(M_1(a^*) = m_1) I(M_2(a^*, m_1) = m_2)\\
&\quad + \sum_{m_2}\sum_{m_1} Y(a^*, m_1, m_2) I(M_1(a^*) = m_1) I(M_2(a^*, m_1) = m_2)\\
&= \sum_{m_2}\sum_{m_1} [Y(a, m_1, m_2) - Y(a^*, m_1, m_2)] I(M_1(a^*) = m_1) I(M_2(a, m_1) = m_2)\\
&\quad - \sum_{m_2}\sum_{m_1} [Y(a, m_1, m_2) - Y(a^*, m_1, m_2)] I(M_1(a^*) = m_1) I(M_2(a^*, m_1) = m_2)\\
&= \sum_{m_2}\sum_{m_1} [Y(a, m_1, m_2) - Y(a^*, m_1, m_2)] I(M_1(a^*) = m_1) \times [I(M_2(a, m_1) = m_2) - I(M_2(a^*, m_1) = m_2)]\\
&= \sum_{m_2}\sum_{m_1} [Y(a, m_1, m_2) I(M_1(a^*) = m_1) - Y(a^*, m_1, m_2) I(M_1(a^*) = m_1)]\\
&\quad \times [I(M_2(a, m_1) = m_2) - I(M_2(a^*, m_1) = m_2)]\\
&= \sum_{m_2}\sum_{m_1} [Y(a, m_1, m_2) I(M_1(a^*) = m_1) - Y(a^*, m_1, m_2) I(M_1(a^*) = m_1)\\
&\quad - Y(a, m_1, m_2^*) I(M_1(a^*) = m_1) + Y(a^*, m_1, m_2^*) I(M_1(a^*) = m_1)]\\
&\quad \times [I(M_2(a, m_1) = m_2) - I(M_2(a^*, m_1) = m_2)],
\end{aligned}$$

where the last equation follows by adding two extra terms which do not change the value of NatINT_{AM_2}.

With the specified conditions, NatINT_{AM_2} can be written as:

$$\begin{aligned}
\text{NatINT}_{AM_2} &= \sum_{m_2}\sum_{m_1} [Y(1, m_1, m_2) I(M_1(0) = m_1) - Y(0, m_1, m_2) I(M_1(0) = m_1)\\
&\quad - Y(1, m_1, 0) I(M_1(0) = m_1) + Y(0, m_1, 0) I(M_1(0) = m_1)]\\
&\quad \times [I(M_2(1, m_1) = m_2) - I(M_2(0, m_1) = m_2)]\\
&= \sum_{m_1} [Y(1, m_1, 0) I(M_1(0) = m_1) - Y(0, m_1, 0) I(M_1(0) = m_1)\\
&\quad - Y(1, m_1, 0) I(M_1(0) = m_1) + Y(0, m_1, 0) I(M_1(0) = m_1)] \times [I(M_2(1, m_1) = 0) - I(M_2(0, m_1) = 0)]\\
&\quad + \sum_{m_1} [Y(1, m_1, 1) I(M_1(0) = m_1) - Y(0, m_1, 1) I(M_1(0) = m_1) - Y(1, m_1, 0) I(M_1(0) = m_1)\\
&\quad + Y(0, m_1, 0) I(M_1(0) = m_1)] \times [I(M_2(1, m_1) = 1) - I(M_2(0, m_1) = 1)]\\
&= \sum_{m_1} [Y(1, m_1, 1) I(M_1(0) = m_1) - Y(0, m_1, 1) I(M_1(0) = m_1) - Y(1, m_1, 0) I(M_1(0) = m_1)\\
&\quad + Y(0, m_1, 0) I(M_1(0) = m_1)] \times [I(M_2(1, m_1) = 1) - I(M_2(0, m_1) = 1)]\\
&= \sum_{m_1} [Y(1, m_1, 1) I(M_1(0) = m_1) - Y(0, m_1, 1) I(M_1(0) = m_1) - Y(1, m_1, 0) I(M_1(0) = m_1)\\
&\quad + Y(0, m_1, 0) I(M_1(0) = m_1)] \times [M_2(1, m_1) - M_2(0, m_1)],
\end{aligned}$$

where the indicator function $I(M_1(0) = m_1)$ indicates that M_1 is at its potential value $M_1(0)$, which may vary with respect to different individuals.

NatINT_{AM_2} can be interpreted as the effect due to the mediation through M_2 and the interaction between A and M_2, conditioning on the potential value of M_1 with the fixed reference level a^*.

The natural MI effect between A, M_1, and M_2 can be rewritten as:

$$\begin{aligned}
\mathrm{NatINT}_{AM_1M_2} = {} & Y(a, M_1(a), M_2(a, M_1(a))) - Y(a^*, M_1(a), M_2(a, M_1(a))) - Y(a, M_1(a^*), M_2(a, M_1(a^*))) \\
& + Y(a^*, M_1(a^*), M_2(a, M_1(a^*))) - Y(a, M_1(a), M_2(a^*, M_1(a))) + Y(a^*, M_1(a), M_2(a^*, M_1(a))) \\
& + Y(a, M_1(a^*), M_2(a^*, M_1(a^*))) - Y(a^*, M_1(a^*), M_2(a^*, M_1(a^*))) \\
= {} & \sum_{m_2}\sum_{m_1} Y(a, m_1, m_2) I(M_1(a) = m_1) I(M_2(a, m_1) = m_2) \\
& - \sum_{m_2}\sum_{m_1} Y(a^*, m_1, m_2) I(M_1(a) = m_1) I(M_2(a, m_1) = m_2) \\
& - \sum_{m_2}\sum_{m_1} Y(a, m_1, m_2) I(M_1(a^*) = m_1) I(M_2(a, m_1) = m_2) \\
& + \sum_{m_2}\sum_{m_1} Y(a^*, m_1, m_2) I(M_1(a^*) = m_1) I(M_2(a, m_1) = m_2) \\
& - \sum_{m_2}\sum_{m_1} Y(a, m_1, m_2) I(M_1(a) = m_1) I(M_2(a^*, m_1) = m_2) \\
& + \sum_{m_2}\sum_{m_1} Y(a^*, m_1, m_2) I(M_1(a) = m_1) I(M_2(a^*, m_1) = m_2) \\
& + \sum_{m_2}\sum_{m_1} Y(a, m_1, m_2) I(M_1(a^*) = m_1) I(M_2(a^*, m_1) = m_2) \\
& - \sum_{m_2}\sum_{m_1} Y(a^*, m_1, m_2) I(M_1(a^*) = m_1) I(M_2(a^*, m_1) = m_2) \\
= {} & \sum_{m_2}\sum_{m_1} [Y(a, m_1, m_2) - Y(a^*, m_1, m_2)] I(M_1(a) = m_1) I(M_2(a, m_1) = m_2) \\
& - \sum_{m_2}\sum_{m_1} [Y(a, m_1, m_2) - Y(a^*, m_1, m_2)] I(M_1(a^*) = m_1) I(M_2(a, m_1) = m_2) \\
& - \sum_{m_2}\sum_{m_1} [Y(a, m_1, m_2) - Y(a^*, m_1, m_2)] I(M_1(a) = m_1) I(M_2(a^*, m_1) = m_2) \\
& + \sum_{m_2}\sum_{m_1} [Y(a, m_1, m_2) - Y(a^*, m_1, m_2)] I(M_1(a^*) = m_1) I(M_2(a^*, m_1) = m_2) \\
= {} & \sum_{m_2}\sum_{m_1} [Y(a, m_1, m_2) - Y(a^*, m_1, m_2)][I(M_1(a) = m_1) - I(M_1(a^*) = m_1)] \\
& \times [I(M_2(a, m_1) = m_2) - I(M_2(a^*, m_1) = m_2)]
\end{aligned}$$

$$\begin{aligned}
\mathrm{NatINT}_{AM_1M_2} = {} & \sum_{m_2}\sum_{m_1} [Y(a, m_1, m_2) - Y(a^*, m_1, m_2) - Y(a, m_1, m_2^*) + Y(a^*, m_1, m_2^*) + Y(a, m_1^*, m_2^*) \\
& - Y(a^*, m_1^*, m_2^*)] \times [I(M_1(a) = m_1) - I(M_1(a^*) = m_1)] \\
& \times [I(M_2(a, m_1) = m_2) - I(M_2(a^*, m_1) = m_2)] + \sum_{m_2}\sum_{m_1} [-Y(a, m_1^*, m_2) + Y(a^*, m_1^*, m_2)] \\
& \times [I(M_1(a) = m_1) - I(M_1(a^*) = m_1)] \times [I(M_2(a, m_1^*) = m_2) - I(M_2(a^*, m_1^*) = m_2)],
\end{aligned}$$

where the last equation follows by adding six extra terms which do not change the value of $\mathrm{NatINT}_{AM_1M_2}$.

With the specified conditions, $\mathrm{NatINT}_{AM_1M_2}$ can be written as:

$$\begin{aligned}
\text{NatINT}_{AM_1M_2} = &\sum_{m_2}\sum_{m_1}[Y(1, m_1, m_2) - Y(0, m_1, m_2) - Y(1, m_1, 0) + Y(0, m_1, 0) + Y(1, 0, 0) - Y(0, 0, 0)] \\
&\times [I(M_1(1) = m_1) - I(M_1(0) = m_1)] \times [I(M_2(1, m_1) = m_2) - I(M_2(0, m_1) = m_2)] \\
&+ \sum_{m_2}\sum_{m_1}[-Y(1, 0, m_2) + Y(0, 0, m_2)] \times [I(M_1(1) = m_1) - I(M_1(0) = m_1)] \\
&\times [I(M_2(1, 0) = m_2) - I(M_2(0, 0) = m_2)] \\
= &\sum_{m_2}[Y(1, 0, m_2) - Y(0, 0, m_2) - Y(1, 0, 0) + Y(0, 0, 0) + Y(1, 0, 0) - Y(0, 0, 0)] \\
&\times [I(M_1(1) = 0) - I(M_1(0) = 0)] \times [I(M_2(1, 0) = m_2) - I(M_2(0, 0) = m_2)] \\
&+ \sum_{m_2}[Y(1, 1, m_2) - Y(0, 1, m_2) - Y(1, 1, 0) + Y(0, 1, 0) + Y(1, 0, 0) - Y(0, 0, 0)] \\
&\times [I(M_1(1) = 1) - I(M_1(0) = 1)] \times [I(M_2(1, 1) = m_2) - I(M_2(0, 1) = m_2)] \\
&+ \sum_{m_2}[-Y(1, 0, m_2) + Y(0, 0, m_2)] \times [I(M_1(1) = 0) - I(M_1(0) = 0)] \\
&\times [I(M_2(1, 0) = m_2) - I(M_2(0, 0) = m_2)] \\
&+ \sum_{m_2}[-Y(1, 0, m_2) + Y(0, 0, m_2)] \times [I(M_1(1) = 1) - I(M_1(0) = 1)] \\
&\times [I(M_2(1, 0) = m_2) - I(M_2(0, 0) = m_2)] \\
= &\sum_{m_2}[Y(1, 1, m_2) - Y(0, 1, m_2) - Y(1, 1, 0) + Y(0, 1, 0) + Y(1, 0, 0) - Y(0, 0, 0)] \\
&\times [I(M_1(1) = 1) - I(M_1(0) = 1)] \times [I(M_2(1, 1) = m_2) - I(M_2(0, 1) = m_2)] \\
&+ \sum_{m_2}[-Y(1, 0, m_2) + Y(0, 0, m_2)] \times [I(M_1(1) = 1) - I(M_1(0) = 1)] \\
&\times [I(M_2(1, 0) = m_2) - I(M_2(0, 0) = m_2)] \\
= &[Y(1, 1, 0) - Y(0, 1, 0) - Y(1, 1, 0) + Y(0, 1, 0) + Y(1, 0, 0) - Y(0, 0, 0)] \\
&\times [I(M_1(1) = 1) - I(M_1(0) = 1)] \times [I(M_2(1, 1) = 0) - I(M_2(0, 1) = 0)] \\
&+ [Y(1, 1, 1) - Y(0, 1, 1) - Y(1, 1, 0) + Y(0, 1, 0) + Y(1, 0, 0) - Y(0, 0, 0)] \\
&\times [I(M_1(1) = 1) - I(M_1(0) = 1)] \times [I(M_2(1, 1) = 1) - I(M_2(0, 1) = 1)] \\
&+ [-Y(1, 0, 0) + Y(0, 0, 0)] \times [I(M_1(1) = 1) - I(M_1(0) = 1)] \\
&\times [I(M_2(1, 0) = 0) - I(M_2(0, 0) = 0)] + [-Y(1, 0, 1) + Y(0, 0, 1)] \\
&\times [I(M_1(1) = 1) - I(M_1(0) = 1)] \times [I(M_2(1, 0) = 1) - I(M_2(0, 0) = 1)]
\end{aligned}$$

$$\begin{aligned}
\text{NatINT}_{AM_1M_2} = &[Y(1,0,0) - Y(0,0,0)] \times [I(M_1(1)=1) - I(M_1(0)=1)] \\
&\times [I(M_2(1,1)=0) - I(M_2(0,1)=0)] \\
&+ [Y(1,1,1) - Y(0,1,1) - Y(1,1,0) + Y(0,1,0) + Y(1,0,0) - Y(0,0,0)] \\
&\times [I(M_1(1)=1) - I(M_1(0)=1)] \times [I(M_2(1,1)=1) - I(M_2(0,1)=1)] \\
&+ [-Y(1,0,0) + Y(0,0,0)] \times [I(M_1(1)=1) - I(M_1(0)=1)] \\
&\times [I(M_2(1,0)=0) - I(M_2(0,0)=0)] + [-Y(1,0,1) + Y(0,0,1)] \\
&\times [I(M_1(1)=1) - I(M_1(0)=1)] \times [I(M_2(1,0)=1) - I(M_2(0,0)=1)] \\
= &-[Y(1,0,0) - Y(0,0,0)] \times [I(M_1(1)=1) - I(M_1(0)=1)] \\
&\times [I(M_2(1,1)=1) - I(M_2(0,1)=1)] + [Y(1,1,1) - Y(0,1,1) - Y(1,1,0) + Y(0,1,0) \\
&+ Y(1,0,0) - Y(0,0,0)] \times [I(M_1(1)=1) - I(M_1(0)=1)] \times [I(M_2(1,1)=1) - I(M_2(0,1)=1)] \\
&+ [Y(1,0,0) - Y(0,0,0)] \times [I(M_1(1)=1) - I(M_1(0)=1)] \times [I(M_2(1,0)=1) - I(M_2(0,0)=1)] \\
&+ [-Y(1,0,1) + Y(0,0,1)] \times [I(M_1(1)=1) - I(M_1(0)=1)] \\
&\times [I(M_2(1,0)=1) - I(M_2(0,0)=1)] \\
= &[Y(1,1,1) - Y(0,1,1) - Y(1,1,0) + Y(0,1,0)] \times [I(M_1(1)=1) - I(M_1(0)=1)] \\
&\times [I(M_2(1,1)=1) - I(M_2(0,1)=1)] + [Y(1,0,0) - Y(0,0,0)] \times [I(M_1(1)=1) - I(M_1(0)=1)] \\
&\times [I(M_2(1,0)=1) - I(M_2(0,0)=1)] + [-Y(1,0,1) + Y(0,0,1)] \\
&\times [I(M_1(1)=1) - I(M_1(0)=1)] \times [I(M_2(1,0)=1) - I(M_2(0,0)=1)] \\
= &[Y(1,1,1) - Y(0,1,1) - Y(1,1,0) + Y(0,1,0)] \times [I(M_1(1)=1) - I(M_1(0)=1)] \\
&\times [I(M_2(1,1)=1) - I(M_2(0,1)=1)] - [Y(1,0,1) - Y(0,0,1) - Y(1,0,0) + Y(0,0,0)] \\
&\times [I(M_1(1)=1) - I(M_1(0)=1)] \times [I(M_2(1,0)=1) - I(M_2(0,0)=1)] \\
= &[Y(1,1,1) - Y(0,1,1) - Y(1,1,0) + Y(0,1,0)] \times [M_1(1) - M_1(0)] \\
&\times [M_2(1,1) - M_2(0,1)] + [-Y(1,0,1) + Y(0,0,1) + Y(1,0,0) - Y(0,0,0)] \\
&\times [M_1(1) - M_1(0)] \times [M_2(1,0) - M_2(0,0)],
\end{aligned}$$

where the six equality follows by the facts that $I(M_2(1,1)=0) = 1 - I(M_2(1,1)=1)$ and $I(M_2(0,1)=0) = 1 - I(M_2(0,1)=1)$.

$\text{NatINT}_{AM_1M_2}$ can be interpreted as the effect due to mediation through both M_1 and M_2, and the interaction between A, M_1, and M_2.

The natural MI effect between M_1 and M_2 can be rewritten as:

$$
\begin{aligned}
\text{NatINT}_{M_1M_2} &= Y(a^*, M_1(a), M_2(a, M_1(a))) - Y(a^*, M_1(a^*), M_2(a, M_1(a^*))) \\
&\quad - Y(a^*, M_1(a), M_2(a^*, M_1(a))) + Y(a^*, M_1(a^*), M_2(a^*, M_1(a^*))) \\
&= \sum_{m_2}\sum_{m_1} Y(a^*, m_1, m_2) I(M_1(a) = m_1) I(M_2(a, m_1) = m_2) \\
&\quad - \sum_{m_2}\sum_{m_1} Y(a^*, m_1, m_2) I(M_1(a^*) = m_1) I(M_2(a, m_1) = m_2) \\
&\quad - \sum_{m_2}\sum_{m_1} Y(a^*, m_1, m_2) I(M_1(a) = m_1) I(M_2(a^*, m_1) = m_2) \\
&\quad + \sum_{m_2}\sum_{m_1} Y(a^*, m_1, m_2) I(M_1(a^*) = m_1) I(M_2(a^*, m_1) = m_2) \\
&= \sum_{m_2}\sum_{m_1} Y(a^*, m_1, m_2)[I(M_1(a) = m_1) - I(M_1(a^*) = m_1)] I(M_2(a, m_1) = m_2) \\
&\quad - \sum_{m_2}\sum_{m_1} Y(a^*, m_1, m_2)[I(M_1(a) = m_1) - I(M_1(a^*) = m_1)] I(M_2(a^*, m_1) = m_2) \\
&= \sum_{m_2}\sum_{m_1} Y(a^*, m_1, m_2)[I(M_1(a) = m_1) - I(M_1(a^*) = m_1)] \times [I(M_2(a, m_1) = m_2) - I(M_2(a^*, m_1) = m_2)] \\
&= \sum_{m_2}\sum_{m_1} [Y(a^*, m_1, m_2) - Y(a^*, m_1, m_2^*) + Y(a^*, m_1^*, m_2^*)] \times [I(M_1(a) = m_1) - I(M_1(a^*) = m_1)] \\
&\quad \times [I(M_2(a, m_1) = m_2) - I(M_2(a^*, m_1) = m_2)] + \sum_{m_2}\sum_{m_1}[-Y(a^*, m_1^*, m_2)] \\
&\quad \times [I(M_1(a) = m_1) - I(M_1(a^*) = m_1)] \times [I(M_2(a, m_1^*) = m_2) - I(M_2(a^*, m_1^*) = m_2)],
\end{aligned}
$$

where the last equality follows by adding three extra terms which do not change the value of $\text{NatINT}_{M_1M_2}$.

With the specified conditions, $\text{NatINT}_{M_1M_2}$ can be written as:

$$
\begin{aligned}
\text{NatINT}_{M_1M_2} &= \sum_{m_2}\sum_{m_1} [Y(0, m_1, m_2) - Y(0, m_1, 0) + Y(0, 0, 0)] \times [I(M_1(1) = m_1) - I(M_1(0) = m_1)] \\
&\quad \times [I(M_2(1, m_1) = m_2) - I(M_2(0, m_1) = m_2)] + \sum_{m_2}\sum_{m_1}[-Y(0, 0, m_2)] \\
&\quad \times [I(M_1(1) = m_1) - I(M_1(0) = m_1)] \times [I(M_2(1, 0) = m_2) - I(M_2(0, 0) = m_2)] \\
&= \sum_{m_2} [Y(0, 0, m_2) - Y(0, 0, 0) + Y(0, 0, 0)] \times [I(M_1(1) = 0) - I(M_1(0) = 0)] \\
&\quad \times [I(M_2(1, 0) = m_2) - I(M_2(0, 0) = m_2)] + \sum_{m_2} [Y(0, 1, m_2) - Y(0, 1, 0) + Y(0, 0, 0)] \\
&\quad \times [I(M_1(1) = 1) - I(M_1(0) = 1)] \times [I(M_2(1, 1) = m_2) - I(M_2(0, 1) = m_2)] \\
&\quad + \sum_{m_2}[-Y(0, 0, m_2)] \times [I(M_1(1) = 0) - I(M_1(0) = 0)] \times [I(M_2(1, 0) = m_2) - I(M_2(0, 0) = m_2)] \\
&\quad + \sum_{m_2}[-Y(0, 0, m_2)] \times [I(M_1(1) = 1) - I(M_1(0) = 1)] \times [I(M_2(1, 0) = m_2) - I(M_2(0, 0) = m_2)] \\
&= \sum_{m_2} [Y(0, 1, m_2) - Y(0, 1, 0) + Y(0, 0, 0)] \times [I(M_1(1) = 1) - I(M_1(0) = 1)] \\
&\quad \times [I(M_2(1, 1) = m_2) - I(M_2(0, 1) = m_2)] \\
&\quad + \sum_{m_2}[-Y(0, 0, m_2)] \times [I(M_1(1) = 1) - I(M_1(0) = 1)] \times [I(M_2(1, 0) = m_2) - I(M_2(0, 0) = m_2)] \\
&= [Y(0, 1, 0) - Y(0, 1, 0) + Y(0, 0, 0)] \times [I(M_1(1) = 1) - I(M_1(0) = 1)] \\
&\quad \times [I(M_2(1, 1) = 0) - I(M_2(0, 1) = 0)] \\
&\quad + [Y(0, 1, 1) - Y(0, 1, 0) + Y(0, 0, 0)] \times [I(M_1(1) = 1) - I(M_1(0) = 1)] \\
&\quad \times [I(M_2(1, 1) = 1) - I(M_2(0, 1) = 1)] + [-Y(0, 0, 0)] \times [I(M_1(1) = 1) - I(M_1(0) = 1)] \\
&\quad \times [I(M_2(1, 0) = 0) - I(M_2(0, 0) = 0)] + [-Y(0, 0, 1)] \\
&\quad \times [I(M_1(1) = 1) - I(M_1(0) = 1)] \times [I(M_2(1, 0) = 1) - I(M_2(0, 0) = 1)]
\end{aligned}
$$

$$\begin{aligned}\text{NatINT}_{M_1M_2} &= [-Y(0,0,0)] \times [I(M_1(1)=1) - I(M_1(0)=1)] \times [I(M_2(1,1)=1) - I(M_2(0,1)=1)]\\ &\quad + [Y(0,1,1) - Y(0,1,0) + Y(0,0,0)] \times [I(M_1(1)=1) - I(M_1(0)=1)]\\ &\quad \times [I(M_2(1,1)=1) - I(M_2(0,1)=1)] + [Y(0,0,0)] \times [I(M_1(1)=1) - I(M_1(0)=1)]\\ &\quad \times [I(M_2(1,0)=1) - I(M_2(0,0)=1)] + [-Y(0,0,1)] \times [I(M_1(1)=1) - I(M_1(0)=1)]\\ &\quad \times [I(M_2(1,0)=1) - I(M_2(0,0)=1)]\\ &= [Y(0,1,1) - Y(0,1,0)] \times [I(M_1(1)=1) - I(M_1(0)=1)]\\ &\quad \times [I(M_2(1,1)=1) - I(M_2(0,1)=1)] + [-Y(0,0,1) + Y(0,0,0)]\\ &\quad \times [I(M_1(1)=1) - I(M_1(0)=1)] \times [I(M_2(1,0)=1) - I(M_2(0,0)=1)]\\ &= [Y(0,1,1) - Y(0,1,0)] \times [M_1(1) - M_1(0)] \times [M_2(1,1) - M_2(0,1)]\\ &\quad + [-Y(0,0,1) + Y(0,0,0)] \times [M_1(1) - M_1(0)] \times [M_2(1,0) - M_2(0,0)],\end{aligned}$$

where the fifth equality follows by the facts that $I(M_2(1,1)=0) = 1 - I(M_2(1,1)=1)$ and $I(M_2(0,1)=0) = 1 - I(M_2(0,1)=1)$.

$\text{NatINT}_{M_1M_2}$ can be interpreted as the effect due to mediation through both M_1 and M_2, and the interaction between M_1 and M_2. Since the interaction is not involved with the change in exposure A, the interpretation can be simply put as the effect due to the mediation through both M_1 and M_2 only.

S3.4 PIE through M_1

The PIE through M_1 can be rewritten as:

$$\begin{aligned}\text{PIE}_{M_1} &= Y(a^*, M_1(a), M_2(a^*, M_1(a))) - Y(a^*, M_1(a^*), M_2(a^*, M_1(a^*)))\\ &= \sum_{m_2}\sum_{m_1} Y(a^*, m_1, m_2) I(M_1(a) = m_1) I(M_2(a^*, m_1) = m_2)\\ &\quad - \sum_{m_2}\sum_{m_1} Y(a^*, m_1, m_2) I(M_1(a^*) = m_1) I(M_2(a^*, m_1) = m_2)\\ &= \sum_{m_2}\sum_{m_1} Y(a^*, m_1, m_2) \times [I(M_1(a) = m_1) - I(M_1(a^*) = m_1)] \times I(M_2(a^*, m_1) = m_2).\end{aligned}$$

With the specified conditions, PIE_{M_1} can be written as:

$$\begin{aligned}\text{PIE}_{M_1} &= \sum_{m_2}\sum_{m_1} Y(0, m_1, m_2) \times [I(M_1(1) = m_1) - I(M_1(0) = m_1)] \times I(M_2(0, m_1) = m_2)\\ &= \sum_{m_2} Y(0,0,m_2) \times [I(M_1(1) = 0) - I(M_1(0) = 0)] \times I(M_2(0,0) = m_2)\\ &\quad + \sum_{m_2} Y(0,1,m_2) \times [I(M_1(1) = 1) - I(M_1(0) = 1)] \times I(M_2(0,1) = m_2)\\ &= -\sum_{m_2} Y(0,0,m_2) \times [I(M_1(1) = 1) - I(M_1(0) = 1)] \times I(M_2(0,0) = m_2)\\ &\quad + \sum_{m_2} Y(0,1,m_2) \times [I(M_1(1) = 1) - I(M_1(0) = 1)] \times I(M_2(0,1) = m_2)\\ &= \sum_{m_2} [Y(0,1,m_2) I(M_2(0,1) = m_2) - Y(0,0,m_2) I(M_2(0,0) = m_2)] \times [I(M_1(1) = 1) - I(M_1(0) = 1)]\\ &= \sum_{m_2} [Y(0,1,m_2) I(M_2(0,1) = m_2) - Y(0,0,m_2) I(M_2(0,0) = m_2)] \times [M_1(1) - M_1(0)],\end{aligned}$$

where the third equation follows by the facts that $I(M_1(1)=0) = 1 - I(M_1(1)=1)$ and $I(M_1(0)=0) = 1 - I(M_1(0)=1)$ and the indicator functions, $I(M_2(0,1) = m_2)$ and $I(M_2(0,0) = m_2)$, indicate that M_2 is at its potential values, which may vary with respect to different individuals.

PIE_{M_1} can be interpreted as the effect due to the mediation through M_1 only, conditioning on the potential values of M_2 with the fixed reference level a^*.

S3.5 Seminatural indirect effect through M_2

The seminatural indirect effect through M_2 can be rewritten as:

$$\begin{aligned}\text{SNIE}_{M_2} &= Y(a^*, M_1(a^*), M_2(a, M_1(a^*))) - Y(a^*, M_1(a^*), M_2(a^*, M_1(a^*)))\\ &= \sum_{m_2}\sum_{m_1} Y(a^*, m_1, m_2) I(M_1(a^*) = m_1) I(M_2(a, m_1) = m_2)\\ &\quad - \sum_{m_2}\sum_{m_1} Y(a^*, m_1, m_2) I(M_1(a^*) = m_1) I(M_2(a^*, m_1) = m_2)\\ &= \sum_{m_2}\sum_{m_1} Y(a^*, m_1, m_2) \times I(M_1(a^*) = m_1) \times [I(M_2(a, m_1) = m_2) - I(M_2(a^*, m_1) = m_2)].\end{aligned}$$

With the specified conditions, SNIE_{M_2} can be written as:

$$\begin{aligned}\text{SNIE}_{M_2} &= \sum_{m_2}\sum_{m_1} Y(0, m_1, m_2) \times I(M_1(0) = m_1) \times [I(M_2(1, m_1) = m_2) - I(M_2(0, m_1) = m_2)]\\ &= \sum_{m_1} Y(0, m_1, 0) \times I(M_1(0) = m_1) \times [I(M_2(1, m_1) = 0) - I(M_2(0, m_1) = 0)]\\ &\quad + \sum_{m_1} Y(0, m_1, 1) \times I(M_1(0) = m_1) \times [I(M_2(1, m_1) = 1) - I(M_2(0, m_1) = 1)]\\ &= -\sum_{m_1} Y(0, m_1, 0) \times I(M_1(0) = m_1) \times [I(M_2(1, m_1) = 1) - I(M_2(0, m_1) = 1)]\\ &\quad + \sum_{m_1} Y(0, m_1, 1) \times I(M_1(0) = m_1) \times [I(M_2(1, m_1) = 1) - I(M_2(0, m_1) = 1)]\\ &= \sum_{m_1} [Y(0, m_1, 1) \times I(M_1(0) = m_1) - Y(0, m_1, 0) \times I(M_1(0) = m_1)]\\ &\quad \times [I(M_2(1, m_1) = 1) - I(M_2(0, m_1) = 1)]\\ &= \sum_{m_1} [Y(0, m_1, 1) \times I(M_1(0) = m_1) - Y(0, m_1, 0) \times I(M_1(0) = m_1)] \times [M_2(1, m_1) - M_2(0, m_1)],\end{aligned}$$

where the third equation follows by the facts that $I(M_2(1, m_1) = 0) = 1 - I(M_2(1, m_1) = 1)$ and $I(M_2(0, m_1) = 0) = 1 - I(M_2(0, m_1) = 1)$ and the indicator functions, $I(M_1(0) = m_1)$, indicates that M_1 is at its potential values, which may vary with respect to different individuals.

SNIE_{M_2} can be interpreted as the effect due to the partial mediation through M_2 only [20], conditioning on the potential values of M_1 with the fixed reference level a^*.

S4 Potential non-identifiability issue of $\text{INT}_{\text{ref-}AM_2}(m_1^*, m_2^*)$ and $\text{INT}_{\text{ref-}AM_1M_2}(m_1^*, m_2^*)$ in a sequential two-mediator scenario

The process that separates $\text{INT}_{\text{ref-}AM_2+AM_1M_2}(m_2^*)$ into $\text{INT}_{\text{ref-}AM_2}(m_1^*, m_2^*)$ and $\text{INT}_{\text{ref-}AM_1M_2}(m_1^*, m_2^*)$ is presented in Supplementary material S3. In this Supplementary material, we would like to show a different process that leads to a variant of the problematic counterfactual formulas proposed by Avin et al. [15]. The corresponding causal structure of a sequential two-mediator scenario is shown in Figure 5.

Proof. From Supplementary material S3, we have the expression of $\text{INT}_{\text{ref-}AM_2+AM_1M_2}(m_2^*)$ in general form:

$$\begin{aligned}
\text{INT}_{\text{ref-}AM_2+AM_1M_2}(m_2^*) &= \sum_{m_2}\sum_{m_1}[Y(a, m_1, m_2) - Y(a, m_1, m_2^*) - Y(a^*, m_1, m_2) + Y(a^*, m_1, m_2^*)] \\
&\quad \times I(M_1(a^*) = m_1) \times I(M_2(a^*, m_1) = m_2) \\
&= \sum_{m_2}\sum_{m_1}[Y(a, m_1, m_2) - Y(a, m_1, m_2^*) - Y(a^*, m_1, m_2) + Y(a^*, m_1, m_2^*) \\
&\quad + Y(a^*, m_1^*, m_2^*) - Y(a^*, m_1^*, m_2^*) + Y(a, m_1^*, m_2^*) - Y(a, m_1^*, m_2^*) \\
&\quad + Y(a^*, m_1^*, m_2) - Y(a^*, m_1^*, m_2) + Y(a, m_1^*, m_2) - Y(a, m_1^*, m_2)] \times I(M_1(a^*) = m_1) \\
&\quad \times I(M_2(a^*, m_1) = m_2) \\
&= \sum_{m_2}\sum_{m_1}[Y(a, m_1^*, m_2) - Y(a^*, m_1^*, m_2) - Y(a, m_1^*, m_2^*) + Y(a^*, m_1^*, m_2^*)] \\
&\quad \times I(M_1(a^*) = m_1) \times I(M_2(a^*, m_1) = m_2) \\
&\quad + \sum_{m_2}\sum_{m_1}[Y(a, m_1, m_2) - Y(a^*, m_1, m_2) - Y(a, m_1^*, m_2) + Y(a^*, m_1^*, m_2) \\
&\quad - Y(a, m_1, m_2^*) + Y(a^*, m_1, m_2^*) + Y(a, m_1^*, m_2^*) - Y(a^*, m_1^*, m_2^*)] \\
&\quad \times I(M_1(a^*) = m_1) \times I(M_2(a^*, m_1) = m_2),
\end{aligned}$$

where the second equality follows by adding and subtracting the same counterfactual formulas.

Therefore, we have the following formulas:

$$\begin{aligned}
\text{INT}_{\text{ref-}AM_2}(m_1^*, m_2^*) &= \sum_{m_2}\sum_{m_1}[Y(a, m_1^*, m_2) - Y(a^*, m_1^*, m_2) - Y(a, m_1^*, m_2^*) + Y(a^*, m_1^*, m_2^*)] \\
&\quad \times I(M_1(a^*) = m_1) \times I(M_2(a^*, m_1) = m_2), \\
\text{INT}_{\text{ref-}AM_1M_2}(m_1^*, m_2^*) &= \sum_{m_2}\sum_{m_1}[Y(a, m_1, m_2) - Y(a^*, m_1, m_2) - Y(a, m_1^*, m_2) + Y(a^*, m_1^*, m_2) \\
&\quad - Y(a, m_1, m_2^*) + Y(a^*, m_1, m_2^*) + Y(a, m_1^*, m_2^*) - Y(a^*, m_1^*, m_2^*)] \\
&\quad \times I(M_1(a^*) = m_1) \times I(M_2(a^*, m_1) = m_2).
\end{aligned}$$

It can be seen that both formulas include the following term:

$$\sum_{m_2}\sum_{m_1} Y(a, m_1^*, m_2) \times I(M_1(a^*) = m_1) \times I(M_2(a^*, m_1) = m_2),$$

which can be rewritten as the counterfactual formula $Y(a, m_1^*, M_2(a^*, M_1(a^*)))$ and graphically illustrated in Figure 6a.

Note that m_1^* is a fixed and arbitrary reference level of M_1. Let us consider an instance that there exists $a^{**} \neq a^*$ such that $M_1(a^{**}) = m_1^*$. In this case, the counterfactual formula can be rewritten as $Y(a, M_1(a^{**}), M_2(a^*, M_1(a^*)))$, where M_1 is being activated by two different values of exposure A in the kite graph formed up by the path $A \to M_1 \to Y$ and the path $A \to M_1 \to M_2 \to Y$ in Figure 5. Avin et al. [15] showed that such counterfactual formulas are non-identifiable and referred to them as problematic counterfactual formulas. Because the instance cannot be ruled out in general, $Y(a, m_1^*, M_2(a^*, M_1(a^*)))$ is non-identifiable. Therefore, $\text{INT}_{\text{ref-}AM_2}(m_1^*, m_2^*)$ and $\text{INT}_{\text{ref-}AM_1M_2}(m_1^*, m_2^*)$ obtained by using the process in this appendix cannot be identified with non-parametric models.

S5 Linear regression models with continuous outcome and continuous mediators in a sequential two-mediator scenario

Suppose we have a directed acyclic graph as shown in Figure 5. Assume the following linear models for Y, M_2, and M_1 are correctly specified:

$$E[Y|A, M_1, M_2, C] = \theta_0 + \theta_1 A + \theta_2 M_1 + \theta_3 M_2 + \theta_4 AM_1 + \theta_5 AM_2 + \theta_6 M_1 M_2 + \theta_7 AM_1 M_2 + \theta_8' C,$$
$$E[M_2|A, M_1, C] = \beta_0 + \beta_1 A + \beta_2 M_1 + \beta_3 AM_1 + \beta_4' C,$$
$$E[M_1|A, C] = \gamma_0 + \gamma_1 A + \gamma_2' C,$$

where C is a sufficient confounding set that satisfies the identification assumptions $(A1)$–(A6); ϵ_Y, ϵ_{M_2} and ϵ_{M_1} denote independent random error terms for Y, M_2, and M_1, followed by $N(0, \sigma_Y^2)$, $N(0, \sigma_{M_2}^2)$, and $N(0, \sigma_{M_1}^2)$, respectively. According to Supplementary material S3, the TE can be decomposed into the following components:

$$\text{TE} = \text{CDE}(m_1^*, m_2^*) + \text{INT}_{\text{ref-}AM_1}(m_1^*, m_2^*) + \text{INT}_{\text{ref-}AM_2}(m_1^*, m_2^*) + \text{INT}_{\text{ref-}AM_1M_2}(m_1^*, m_2^*) + \text{NatINT}_{AM_1} + \text{NatINT}_{AM_2} + \text{NatINT}_{AM_1M_2} + \text{NatINT}_{M_1M_2} + \text{PIE}_{M_1} + \text{SNIE}_{M_2}.$$

The expected value of each component conditional on the sufficient confounding set is presented in the following.

S5.1 CDE

$$\begin{aligned}
\text{CDE}(m_1^*, m_2^*) &= Y(a, m_1^*, m_2^*) - Y(a^*, m_1^*, m_2^*) \\
\Rightarrow \quad E[\text{CDE}(m_1^*, m_2^*)|c] &= E[Y(a, m_1^*, m_2^*) - Y(a^*, m_1^*, m_2^*)|c] \\
&= E[Y(a, m_1^*, m_2^*)|c] - E[Y(a^*, m_1^*, m_2^*)|c] \\
&= E[Y(a, m_1^*, m_2^*)|a, c] - E[Y(a^*, m_1^*, m_2^*)|a^*, c] \quad \text{by } A1 \\
&= E[Y(a, m_1^*, m_2^*)|a, m_1^*, m_2^*, c] - E[Y(a^*, m_1^*, m_2^*)|a^*, m_1^*, m_2^*, c] \quad \text{by } A2 \\
&= E[Y|a, m_1^*, m_2^*, c] - E[Y|a^*, m_1^*, m_2^*, c] \quad \text{by consistency} \\
&= (\theta_0 + \theta_1 a + \theta_2 m_1^* + \theta_3 m_2^* + \theta_4 a m_1^* + \theta_5 a m_2^* + \theta_6 m_1^* m_2^* + \theta_7 a m_1^* m_2^* + \theta_8' c) \\
&\quad - (\theta_0 + \theta_1 a^* + \theta_2 m_1^* + \theta_3 m_2^* + \theta_4 a^* m_1^* + \theta_5 a^* m_2^* + \theta_6 m_1^* m_2^* + \theta_7 a^* m_1^* m_2^* + \theta_8' c) \\
&= (\theta_1 a + \theta_4 a m_1^* + \theta_5 a m_2^* + \theta_7 a m_1^* m_2^*) - (\theta_1 a^* + \theta_4 a^* m_1^* + \theta_5 a^* m_2^* + \theta_7 a^* m_1^* m_2^*) \\
&= \theta_1(a - a^*) + \theta_4 m_1^*(a - a^*) + \theta_5 m_2^*(a - a^*) + \theta_7 m_1^* m_2^*(a - a^*) \\
&= (\theta_1 + \theta_4 m_1^* + \theta_5 m_2^* + \theta_7 m_1^* m_2^*)(a - a^*).
\end{aligned}$$

S5.2 Reference interaction effect between *A* and M_1

We first consider M_1 as a categorical random variable.

$$\text{INT}_{\text{ref-}AM_1}(m_1^*, m_2^*) = \sum_{m_1} [Y(a, m_1, m_2^*) - Y(a^*, m_1, m_2^*) - Y(a, m_1^*, m_2^*) + Y(a^*, m_1^*, m_2^*)] \times I(M_1(a^*) = m_1)$$

$$\Rightarrow E\left[\text{INT}_{\text{ref-}AM_1}(m_1^*, m_2^*)|c\right]$$
$$= E\left[\sum_{m_1}[Y(a, m_1, m_2^*) - Y(a^*, m_1, m_2^*) - Y(a, m_1^*, m_2^*) + Y(a^*, m_1^*, m_2^*)] \times I(M_1(a^*) = m_1)|c\right]$$
$$= \sum_{m_1} E[[Y(a, m_1, m_2^*) - Y(a^*, m_1, m_2^*) - Y(a, m_1^*, m_2^*) + Y(a^*, m_1^*, m_2^*)] \times I(M_1(a^*) = m_1)|c]$$
$$= \sum_{m_1} E[Y(a, m_1, m_2^*) - Y(a^*, m_1, m_2^*) - Y(a, m_1^*, m_2^*) + Y(a^*, m_1^*, m_2^*)|c] \times E[I(M_1(a^*) = m_1)|c] \quad \text{by } A4$$
$$= \sum_{m_1} E[Y(a, m_1, m_2^*) - Y(a^*, m_1, m_2^*) - Y(a, m_1^*, m_2^*) + Y(a^*, m_1^*, m_2^*)|c] \times \Pr(M_1(a^*) = m_1|c)$$
$$= \sum_{m_1} E[Y(a, m_1, m_2^*) - Y(a^*, m_1, m_2^*) - Y(a, m_1^*, m_2^*) + Y(a^*, m_1^*, m_2^*)|c] \times \Pr(M_1(a^*) = m_1|a^*, c) \quad \text{by } A3$$
$$= \sum_{m_1} E[Y(a, m_1, m_2^*) - Y(a^*, m_1, m_2^*) - Y(a, m_1^*, m_2^*) + Y(a^*, m_1^*, m_2^*)|c]$$
$$\times \Pr(M_1 = m_1|a^*, c) \quad \text{by consistency}$$
$$= \sum_{m_1} E[Y(a, m_1, m_2^*)|c]\Pr(M_1 = m_1|a^*, c) - \sum_{m_1} E[Y(a^*, m_1, m_2^*)|c]\Pr(M_1 = m_1|a^*, c)$$
$$- \sum_{m_1} E[Y(a, m_1^*, m_2^*)|c]\Pr(M_1 = m_1|a^*, c) + \sum_{m_1} E[Y(a^*, m_1^*, m_2^*)|c]\Pr(M_1 = m_1|a^*, c)$$
$$= \sum_{m_1} E[Y(a, m_1, m_2^*)|a, m_1, m_2^*, c]\Pr(M_1 = m_1|a^*, c) - \sum_{m_1} E[Y(a^*, m_1, m_2^*)|a^*, m_1, m_2^*, c]\Pr(M_1 = m_1|a^*, c)$$
$$- \sum_{m_1} E[Y(a, m_1^*, m_2^*)|a, m_1^*, m_2^*, c]\Pr(M_1 = m_1|a^*, c) + \sum_{m_1} E[Y(a^*, m_1^*, m_2^*)|a^*, m_1^*, m_2^*, c]\Pr(M_1$$
$$= m_1|a^*, c) \quad \text{by A1 A2}$$
$$= \sum_{m_1} E[Y|a, m_1, m_2^*, c]\Pr(M_1 = m_1|a^*, c) - \sum_{m_1} E[Y|a^*, m_1, m_2^*, c]\Pr(M_1 = m_1|a^*, c)$$
$$- \sum_{m_1} E[Y|a, m_1^*, m_2^* \, c]\Pr(M_1 = m_1|a^*, c) + \sum_{m_1} E[Y|a^*, m_1^*, m_2^*, c]\Pr(M_1 = m_1|a^*, c) \ \text{by consistency}$$

We next extend the formula to consider a continuous M_1.

$$E\left[\text{INT}_{\text{ref-}AM_1}(m_1^*, m_2^*)|c\right]$$
$$= \int_{m_1} E[Y|a, m_1, m_2^*, c]d\Pr(M_1 = m_1|a^*, c) - \int_{m_1} E[Y|a^*, m_1, m_2^*, c]d\Pr(M_1 = m_1|a^*, c)$$
$$- \int_{m_1} E[Y|a, m_1^*, m_2^*, c]d\Pr(M_1 = m_1|a^*, c) + \int_{m_1} E[Y|a^*, m_1^*, m_2^*, c]d\Pr(M_1 = m_1|a^*, c)$$
$$= \int_{m_1} (\theta_0 + \theta_1 a + \theta_2 m_1 + \theta_3 m_2^* + \theta_4 a m_1 + \theta_5 a m_2^* + \theta_6 m_1 m_2^* + \theta_7 a m_1 m_2^* + \theta_8' c)d\Pr(M_1 = m_1|a^*, c)$$
$$- \int_{m_1} (\theta_0 + \theta_1 a^* + \theta_2 m_1 + \theta_3 m_2^* + \theta_4 a^* m_1 + \theta_5 a^* m_2^* + \theta_6 m_1 m_2^* + \theta_7 a^* m_1 m_2^* + \theta_8' c)d\Pr(M_1 = m_1|a^*, c)$$
$$- \int_{m_1} (\theta_0 + \theta_1 a + \theta_2 m_1^* + \theta_3 m_2^* + \theta_4 a m_1^* + \theta_5 a m_2^* + \theta_6 m_1^* m_2^* + \theta_7 a m_1^* m_2^* + \theta_8' c)d\Pr(M_1 = m_1|a^*, c)$$

$$+\int_{m_1}(\theta_0+\theta_1a^*+\theta_2m_1^*+\theta_3m_2^*+\theta_4a^*m_1^*+\theta_5a^*m_2^*+\theta_6m_1^*m_2^*+\theta_7a^*m_1^*m_2^*+\theta_8'c)\mathrm{dPr}(M_1=m_1|a^*,c)$$
$$\begin{aligned}
&=(\theta_0+\theta_1a+\theta_3m_2^*+\theta_5am_2^*+\theta_8'c)+(\theta_2+\theta_4a+\theta_6m_2^*+\theta_7am_2^*)\times(\gamma_0+\gamma_1a^*+\gamma_2'c)\\
&\quad-(\theta_0+\theta_1a^*+\theta_3m_2^*+\theta_5a^*m_2^*+\theta_8'c)-(\theta_2+\theta_4a^*+\theta_6m_2^*+\theta_7a^*m_2^*)\times(\gamma_0+\gamma_1a^*+\gamma_2'c)\\
&\quad-(\theta_0+\theta_1a+\theta_2m_1^*+\theta_3m_2^*+\theta_4am_1^*+\theta_5am_2^*+\theta_6m_1^*m_2^*+\theta_7am_1^*m_2^*+\theta_8'c)\\
&\quad+(\theta_0+\theta_1a^*+\theta_2m_1^*+\theta_3m_2^*+\theta_4a^*m_1^*+\theta_5a^*m_2^*+\theta_6m_1^*m_2^*+\theta_7a^*m_1^*m_2^*+\theta_8'c)\\
&=(\gamma_0+\gamma_1a^*+\gamma_2'c-m_1^*)\times(\theta_4+\theta_7m_2^*)\times(a-a^*).
\end{aligned}$$

S5.3 Reference interaction effect between *A* and M_2

From Supplementary material S3, we know the $\mathrm{INT}_{\text{ref-}AM_2}(m_1^*, m_2^*)$ in general form:

$$\begin{aligned}
\mathrm{INT}_{\text{ref-}AM_2}(m_1^*,m_2^*)=&\sum_{m_2}\sum_{m_1}[Y(a,m_1^*,m_2)-Y(a^*,m_1^*,m_2)]\times I(M_1(a^*)=m_1)\times I(M_2(a^*,m_1^*)=m_2)\\
&+\sum_{m_2}\sum_{m_1}[-Y(a,m_1^*,m_2^*)+Y(a^*,m_1^*,m_2^*)]\times I(M_1(a^*)=m_1)\times I(M_2(a^*,m_1)=m_2).
\end{aligned}$$

We can split $\mathrm{INT}_{\text{ref-}AM_2}(m_1^*, m_2^*)$ into two parts and let

$$(1)=\sum_{m_2}\sum_{m_1}[Y(a,m_1^*,m_2)-Y(a^*,m_1^*,m_2)]\times I(M_1(a^*)=m_1)\times I(M_2(a^*,m_1^*)=m_2)$$

and

$$(2)=\sum_{m_2}\sum_{m_1}[-Y(a,m_1^*,m_2^*)+Y(a^*,m_1^*,m_2^*)]\times I(M_1(a^*)=m_1)\times I(M_2(a^*,m_1)=m_2).$$

It can be seen that $(2)=-Y(a,m_1^*,m_2^*)+Y(a^*,m_1^*,m_2^*)$, which is the additive inverse of $\mathrm{CDE}(m_1^*, m_2^*)$. Therefore, we have

$$\begin{aligned}
E\left[\mathrm{INT}_{\text{ref-}AM_2}(m_1^*,m_2^*)|c\right]&=E[(1)+(2)|c]\\
&=E[(1)|c]+E[(2)|c]\\
&=E[(1)|c]-E[-(2)|c]\\
&=E[(1)|c]-E[\mathrm{CDE}(m_1^*,m_2^*)|c]\\
&=E[(1)|c]-(\theta_1+\theta_4m_1^*+\theta_5m_2^*+\theta_7m_1^*m_2^*)(a-a^*).
\end{aligned}$$

The formula of $E[(1)|c]$ can be found by the following derivation:

$$
\begin{aligned}
E[(1)|c] &= E\left[\sum_{m_2}\sum_{m_1}[Y(a, m_1^*, m_2) - Y(a^*, m_1^*, m_2)] \times I(M_1(a^*) = m_1) \times I(M_2(a^*, m_1^*) = m_2)|c\right] \\
&= \sum_{m_2}\sum_{m_1}E[[Y(a, m_1^*, m_2) - Y(a^*, m_1^*, m_2)] \times I(M_1(a^*) = m_1) \times I(M_2(a^*, m_1^*) = m_2)|c] \\
&= \sum_{m_2}\sum_{m_1}E[Y(a, m_1^*, m_2) - Y(a^*, m_1^*, m_2)|c] \times E[I(M_1(a^*) = m_1)|c] \times E[I(M_2(a^*, m_1^*) = m_2)|c] \text{ by A4 A6} \\
&= \sum_{m_2}\sum_{m_1}E[Y(a, m_1^*, m_2) - Y(a^*, m_1^*, m_2)|c] \times \Pr(M_1(a^*) = m_1|c) \times \Pr(M_2(a^*, m_1^*) = m_2|c) \\
&= \sum_{m_2}\sum_{m_1}E[Y(a, m_1^*, m_2) - Y(a^*, m_1^*, m_2)|c] \\
&\quad \times \Pr(M_1(a^*) = m_1|a^*, c) \times \Pr(M_2(a^*, m_1^*) = m_2|a^*, m_1^*, c) \text{ by A3 A5} \\
&= \sum_{m_2}\sum_{m_1}E[Y(a, m_1^*, m_2) - Y(a^*, m_1^*, m_2)|c] \times \Pr(M_1 = m_1|a^*, c) \times \Pr(M_2 = m_2|a^*, m_1^*, c) \text{ by consistency} \\
&= \sum_{m_2}\sum_{m_1}E[Y(a, m_1^*, m_2)|c] \times \Pr(M_1 = m_1|a^*, c) \times \Pr(M_2 = m_2|a^*, m_1^*, c) \\
&\quad - \sum_{m_2}\sum_{m_1}E[Y(a^*, m_1^*, m_2)|c] \times \Pr(M_1 = m_1|a^*, c) \times \Pr(M_2 = m_2|a^*, m_1^*, c) \\
&= \sum_{m_2}\sum_{m_1}E[Y(a, m_1^*, m_2)|a, m_1^*, m_2, c] \times \Pr(M_1 = m_1|a^*, c) \times \Pr(M_2 = m_2|a^*, m_1^*, c) \\
&\quad - \sum_{m_2}\sum_{m_1}E[Y(a^*, m_1^*, m_2)|a^*, m_1^*, m_2, c] \times \Pr(M_1 = m_1|a^*, c) \times \Pr(M_2 = m_2|a^*, m_1^*, c) \; by \; A1 \; A2 \\
&= \sum_{m_2}\sum_{m_1}E[Y|a, m_1^*, m_2, c] \times \Pr(M_1 = m_1|a^*, c) \times \Pr(M_2 = m_2|a^*, m_1^*, c) \\
&\quad - \sum_{m_2}\sum_{m_1}E[Y|a^*, m_1^*, m_2, c] \times \Pr(M_1 = m_1|a^*, c) \times \Pr(M_2 = m_2|a^*, m_1^*, c) \text{ by consistency} \\
&= \int_{m_2}\int_{m_1}E[Y|a, m_1^*, m_2, c]d\Pr(M_1 = m_1|a^*, c)d\Pr(M_2 = m_2|a^*, m_1^*, c) \\
&\quad - \int_{m_2}\int_{m_1}E[Y|a^*, m_1^*, m_2, c]d\Pr(M_1 = m_1|a^*, c)d\Pr(M_2 = m_2|a^*, m_1^*, c)
\end{aligned}
$$

$$= \int_{m_2} E[Y|a, m_1^*, m_2, c] \int_{m_1} \mathrm{dPr}(M_1 = m_1|a^*, c)\mathrm{dPr}(M_2 = m_2|a^*, m_1^*, c)$$
$$- \int_{m_2} E[Y|a^*, m_1^*, m_2, c] \int_{m_1} \mathrm{dPr}(M_1 = m_1|a^*, c)\mathrm{dPr}(M_2 = m_2|a^*, m_1^*, c)$$
$$= \int_{m_2} E[Y|a, m_1^*, m_2, c]\mathrm{dPr}(M_2 = m_2|a^*, m_1^*, c) - \int_{m_2} E[Y|a^*, m_1^*, m_2, c]\mathrm{dPr}(M_2 = m_2|a^*, m_1^*, c)$$
$$= \int_{m_2} (\theta_0 + \theta_1 a + \theta_2 m_1^* + \theta_3 m_2 + \theta_4 a m_1^* + \theta_5 a m_2 + \theta_6 m_1^* m_2 + \theta_7 a m_1^* m_2 + \theta_8' c)\mathrm{dPr}(M_2 = m_2|a^*, m_1^*, c)$$
$$- \int_{m_2} (\theta_0 + \theta_1 a^* + \theta_2 m_1^* + \theta_3 m_2 + \theta_4 a^* m_1^* + \theta_5 a^* m_2 + \theta_6 m_1^* m_2 + \theta_7 a^* m_1^* m_2 + \theta_8' c)\mathrm{dPr}(M_2 = m_2|a^*, m_1^*, c)$$
$$= (\theta_0 + \theta_1 a + \theta_2 m_1^* + \theta_4 a m_1^* + \theta_8' c) + (\theta_3 + \theta_5 a + \theta_6 m_1^* + \theta_7 a m_1^*) \int_{m_2} m_2 \mathrm{dPr}(M_2 = m_2|a^*, m_1^*, c)$$
$$- (\theta_0 + \theta_1 a^* + \theta_2 m_1^* + \theta_4 a^* m_1^* + \theta_8' c) + (\theta_3 + \theta_5 a^* + \theta_6 m_1^* + \theta_7 a^* m_1^*) \int_{m_2} m_2 \mathrm{dPr}(M_2 = m_2|a^*, m_1^*, c)$$
$$= (\theta_1 + \theta_4 m_1^*)a + (\theta_3 + \theta_5 a + \theta_6 m_1^* + \theta_7 a m_1^*)(\beta_0 + \beta_1 a^* + \beta_2 m_1^* + \beta_3 a^* m_1^* + \beta_4' c)$$
$$- (\theta_1 + \theta_4 m_1^*)a^* - (\theta_3 + \theta_5 a^* + \theta_6 m_1^* + \theta_7 a^* m_1^*)(\beta_0 + \beta_1 a^* + \beta_2 m_1^* + \beta_3 a^* m_1^* + \beta_4' c)$$
$$= [(\theta_1 + \theta_4 m_1^*) + (\theta_5 + \theta_7 m_1^*)(\beta_0 + \beta_1 a^* + \beta_2 m_1^* + \beta_3 a^* m_1^* + \beta_4' c)](a - a^*).$$

Therefore, $E[\mathrm{INT}_{\text{ref-}AM_2}(m_1^*, m_2^*)|c]$ can be found by the following derivation:

$$E\left[\mathrm{INT}_{\text{ref-}AM_2}(m_1^*, m_2^*)|c\right] = E[(1)|c] - (\theta_1 + \theta_4 m_1^* + \theta_5 m_2^* + \theta_7 m_1^* m_2^*)(a - a^*)$$
$$= (\theta_5 + \theta_7 m_1^*)(\beta_0 + \beta_1 a^* + \beta_2 m_1^* + \beta_3 a^* m_1^* + \beta_4' c - m_2^*)(a - a^*).$$

S5.4 Reference interaction effect between A, M_1, and M_2

We first find the formula of $E[\mathrm{INT}_{\text{ref-}AM_2+AM_1M_2}(m_2^*)|c]$:

$$\mathrm{INT}_{\text{ref-}AM_2+AM_1M_2}(m_2^*)$$
$$= \sum_{m_2}\sum_{m_1} [Y(a, m_1, m_2) - Y(a, m_1, m_2^*) - Y(a^*, m_1, m_2) + Y(a^*, m_1, m_2^*)] \times I(M_1(a^*) = m_1)$$
$$\times I(M_2(a^*, m_1) = m_2)$$

$$
\begin{aligned}
&\Rightarrow E\Big[\mathrm{INT}_{\text{ref-}AM_2+AM_1M_2}(m_2^*)|c\Big]\\
&= E\Bigg[\sum_{m_2}\sum_{m_1}[Y(a,m_1,m_2)-Y(a,m_1,m_2^*)-Y(a^*,m_1,m_2)+Y(a^*,m_1,m_2^*)]\times I(M_1(a^*)=m_1)\\
&\quad\times I(M_2(a^*,m_1)=m_2)\Bigg|\,c\Bigg]\\
&= \sum_{m_2}\sum_{m_1}E[[Y(a,m_1,m_2)-Y(a,m_1,m_2^*)-Y(a^*,m_1,m_2)+Y(a^*,m_1,m_2^*)]\times I(M_1(a^*)=m_1)\\
&\quad\times I(M_2(a^*,m_1)=m_2)|c]\\
&= \sum_{m_2}\sum_{m_1}E[Y(a,m_1,m_2)-Y(a,m_1,m_2^*)-Y(a^*,m_1,m_2)+Y(a^*,m_1,m_2^*)|c]\times E[I(M_1(a^*)=m_1)|c]\\
&\quad\times E[I(M_2(a^*,m_1)=m_2)|c]\text{ by }A4\ A6\\
&= \sum_{m_2}\sum_{m_1}E[Y(a,m_1,m_2)-Y(a,m_1,m_2^*)-Y(a^*,m_1,m_2)+Y(a^*,m_1,m_2^*)|c]\times \Pr(M_1(a^*)=m_1|c)\\
&\quad\times \Pr(M_2(a^*,m_1)=m_2|c)\\
&= \sum_{m_2}\sum_{m_1}E[Y(a,m_1,m_2)-Y(a,m_1,m_2^*)-Y(a^*,m_1,m_2)+Y(a^*,m_1,m_2^*)|c]\times \Pr(M_1(a^*)=m_1|a^*,c)\\
&\quad\times \Pr(M_2(a^*,m_1)=m_2)|a^*,m_1,c\text{ by }A3\ A5\\
&= \sum_{m_2}\sum_{m_1}E[Y(a,m_1,m_2)-Y(a,m_1,m_2^*)-Y(a^*,m_1,m_2)+Y(a^*,m_1,m_2^*)|c]\times \Pr(M_1=m_1|a^*,c)\\
&\quad\times \Pr(M_2=m_2|a^*,m_1,c)\text{ by consistency}\\
&= \sum_{m_2}\sum_{m_1}E[Y(a,m_1,m_2)|c]\times \Pr(M_1=m_1|a^*,c)\times \Pr(M_2=m_2|a^*,m_1,c)\\
&\quad-\sum_{m_2}\sum_{m_1}E[Y(a,m_1,m_2^*)|c]\times \Pr(M_1=m_1|a^*,c)\times \Pr(M_2=m_2|a^*,m_1,c)\\
&\quad-\sum_{m_2}\sum_{m_1}E[Y(a^*,m_1,m_2)|c]\times \Pr(M_1=m_1|a^*,c)\times \Pr(M_2=m_2|a^*,m_1,c)\\
&\quad+\sum_{m_2}\sum_{m_1}E[Y(a^*,m_1,m_2^*)|c]\times \Pr(M_1=m_1|a^*,c)\times \Pr(M_2=m_2|a^*,m_1,c)\\
&= \sum_{m_2}\sum_{m_1}E[Y(a,m_1,m_2)|a,m_1,m_2,c]\times \Pr(M_1=m_1|a^*,c)\times \Pr(M_2=m_2|a^*,m_1,c)\\
&\quad-\sum_{m_2}\sum_{m_1}E[Y(a,m_1,m_2^*)|a,m_1,m_2^*,c]\times \Pr(M_1=m_1|a^*,c)\times \Pr(M_2=m_2|a^*,m_1,c)\\
&\quad-\sum_{m_2}\sum_{m_1}E[Y(a^*,m_1,m_2)|a^*,m_1,m_2,c]\times \Pr(M_1=m_1|a^*,c)\times \Pr(M_2=m_2|a^*,m_1,c)\\
&\quad+\sum_{m_2}\sum_{m_1}E[Y(a^*,m_1,m_2^*)|a^*,m_1,m_2^*,c]\times \Pr(M_1=m_1|a^*,c)\times \Pr(M_2=m_2|a^*,m_1,c)\text{ by }A1\ A2
\end{aligned}
$$

$$
\begin{aligned}
&= \sum_{m_2}\sum_{m_1} E[Y|a, m_1, m_2, c] \times \Pr(M_1 = m_1|a^*, c) \times \Pr(M_2 = m_2|a^*, m_1, c) \\
&\quad - \sum_{m_2}\sum_{m_1} E[Y|a, m_1, m_2^*, c] \times \Pr(M_1 = m_1|a^*, c) \times \Pr(M_2 = m_2|a^*, m_1, c) \\
&\quad - \sum_{m_2}\sum_{m_1} E[Y|a^*, m_1, m_2, c] \times \Pr(M_1 = m_1|a^*, c) \times \Pr(M_2 = m_2|a^*, m_1, c) \\
&\quad + \sum_{m_2}\sum_{m_1} E[Y|a^*, m_1, m_2^*, c] \times \Pr(M_1 = m_1|a^*, c) \times \Pr(M_2 = m_2|a^*, m_1, c) \text{ by consistency} \\
&= \int_{m_2}\int_{m_1} E[Y|a, m_1, m_2, c] \mathrm{d}\Pr(M_1 = m_1|a^*, c) \mathrm{d}\Pr(M_2 = m_2|a^*, m_1, c) \\
&\quad - \int_{m_2}\int_{m_1} E[Y|a, m_1, m_2^*, c] \mathrm{d}\Pr(M_1 = m_1|a^*, c) \mathrm{d}\Pr(M_2 = m_2|a^*, m_1, c) \\
&\quad - \int_{m_2}\int_{m_1} E[Y|a^*, m_1, m_2, c] \mathrm{d}\Pr(M_1 = m_1|a^*, c) \mathrm{d}\Pr(M_2 = m_2|a^*, m_1, c) \\
&\quad + \int_{m_2}\int_{m_1} E[Y|a^*, m_1, m_2^*, c] \mathrm{d}\Pr(M_1 = m_1|a^*, c) \mathrm{d}\Pr(M_2 = m_2|a^*, m_1, c) \\
&= \int_{m_1}\int_{m_2} E[Y|a, m_1, m_2, c] \mathrm{d}\Pr(M_2 = m_2|a^*, m_1, c) \mathrm{d}\Pr(M_1 = m_1|a^*, c) \\
&\quad - \int_{m_1}\int_{m_2} E[Y|a, m_1, m_2^*, c] \mathrm{d}\Pr(M_2 = m_2|a^*, m_1, c) \mathrm{d}\Pr(M_1 = m_1|a^*, c) \\
&\quad - \int_{m_1}\int_{m_2} E[Y|a^*, m_1, m_2, c] \mathrm{d}\Pr(M_2 = m_2|a^*, m_1, c) \mathrm{d}\Pr(M_1 = m_1|a^*, c) \\
&\quad + \int_{m_1}\int_{m_2} E[Y|a^*, m_1, m_2^*, c] \mathrm{d}\Pr(M_2 = m_2|a^*, m_1, c) \mathrm{d}\Pr(M_1 = m_1|a^*, c) \\
&= \int_{m_1}\int_{m_2} (\theta_0 + \theta_1 a + \theta_2 m_1 + \theta_3 m_2 + \theta_4 a m_1 + \theta_5 a m_2 + \theta_6 m_1 m_2 + \theta_7 a m_1 m_2 \\
&\quad + \theta_8' c) \mathrm{d}\Pr(M_2 = m_2|a^*, m_1, c) \mathrm{d}\Pr(M_1 = m_1|a^*, c) \\
&\quad - \int_{m_1}\int_{m_2} (\theta_0 + \theta_1 a + \theta_2 m_1 + \theta_3 m_2^* + \theta_4 a m_1 + \theta_5 a m_2^* + \theta_6 m_1 m_2^* + \theta_7 a m_1 m_2^* \\
&\quad + \theta_8' c) \mathrm{d}\Pr(M_2 = m_2|a^*, m_1, c) \mathrm{d}\Pr(M_1 = m_1|a^*, c) \\
&\quad - \int_{m_1}\int_{m_2} (\theta_0 + \theta_1 a^* + \theta_2 m_1 + \theta_3 m_2 + \theta_4 a^* m_1 + \theta_5 a^* m_2 + \theta_6 m_1 m_2 + \theta_7 a^* m_1 m_2 \\
&\quad + \theta_8' c) \mathrm{d}\Pr(M_2 = m_2|a^*, m_1, c) \mathrm{d}\Pr(M_1 = m_1|a^*, c)
\end{aligned}
$$

$$
\begin{aligned}
&+ \int_{m_1}\int_{m_2} (\theta_0 + \theta_1 a^* + \theta_2 m_1 + \theta_3 m_2^* + \theta_4 a^* m_1 + \theta_5 a^* m_2^* + \theta_6 m_1 m_2^* + \theta_7 a^* m_1 m_2^* \\
&+ \theta_8' c)\mathrm{dPr}(M_2 = m_2|a^*, m_1, c)\mathrm{dPr}(M_1 = m_1|a^*, c) \\
= &\int_{m_1} [(\theta_0 + \theta_1 a + \theta_2 m_1 + \theta_4 a m_1 + \theta_8' c) + (\theta_3 + \theta_5 a + \theta_6 m_1 + \theta_7 a m_1) \\
&\times (\beta_0 + \beta_1 a^* + \beta_2 m_1 + \beta_3 a^* m_1 + \beta_4' c)]\mathrm{dPr}(M_1 = m_1|a^*, c) \\
&- \int_{m_1} (\theta_0 + \theta_1 a + \theta_2 m_1 + \theta_3 m_2^* + \theta_4 a m_1 + \theta_5 a m_2^* + \theta_6 m_1 m_2^* + \theta_7 a m_1 m_2^* + \theta_8' c)\mathrm{dPr}(M_1 = m_1|a^*, c) \\
&- \int_{m_1} [(\theta_0 + \theta_1 a^* + \theta_2 m_1 + \theta_4 a^* m_1 + \theta_8' c) + (\theta_3 + \theta_5 a^* + \theta_6 m_1 + \theta_7 a^* m_1) \\
&\times (\beta_0 + \beta_1 a^* + \beta_2 m_1 + \beta_3 a^* m_1 + \beta_4' c)]\mathrm{dPr}(M_1 = m_1|a^*, c) \\
&+ \int_{m_1} (\theta_0 + \theta_1 a^* + \theta_2 m_1 + \theta_3 m_2^* + \theta_4 a^* m_1 + \theta_5 a^* m_2^* + \theta_6 m_1 m_2^* + \theta_7 a^* m_1 m_2^* + \theta_8' c)\mathrm{dPr}(M_1 = m_1|a^*, c) \\
= &(\theta_0 + \theta_1 a + \theta_8' c) + (\theta_3 + \theta_5 a)(\beta_0 + \beta_1 a^* + \beta_4' c) + (\theta_2 + \theta_4 a)(\gamma_0 + \gamma_1 a^* + \gamma_2' c) \\
&+ (\theta_6 + \theta_7 a)(\beta_0 + \beta_1 a^* + \beta_4' c)(\gamma_0 + \gamma_1 a^* + \gamma_2' c) \\
&+ (\theta_3 + \theta_5 a)(\beta_2 + \beta_3 a^*)(\gamma_0 + \gamma_1 a^* + \gamma_2' c) + (\theta_6 + \theta_7 a)(\beta_2 + \beta_3 a^*)[\sigma_{M_1}^2 + (\gamma_0 + \gamma_1 a^* + \gamma_2' c)^2] \\
&- (\theta_0 + \theta_1 a + \theta_3 m_2^* + \theta_5 a m_2^* + \theta_8' c) - (\theta_2 + \theta_4 a + \theta_6 m_2^* + \theta_7 a m_2^*)(\gamma_0 + \gamma_1 a^* + \gamma_2' c) \\
&- (\theta_0 + \theta_1 a^* + \theta_8' c) - (\theta_3 + \theta_5 a^*)(\beta_0 + \beta_1 a^* + \beta_4' c) - (\theta_2 + \theta_4 a^*)(\gamma_0 + \gamma_1 a^* + \gamma_2' c) \\
&- (\theta_6 + \theta_7 a^*)(\beta_0 + \beta_1 a^* + \beta_4' c)(\gamma_0 + \gamma_1 a^* + \gamma_2' c) - (\theta_3 + \theta_5 a^*)(\beta_2 + \beta_3 a^*)(\gamma_0 + \gamma_1 a^* + \gamma_2' c) \\
&- (\theta_6 + \theta_7 a^*)(\beta_2 + \beta_3 a^*)[\sigma_{M_1}^2 + (\gamma_0 + \gamma_1 a^* + \gamma_2' c)^2] + (\theta_0 + \theta_1 a^* + \theta_3 m_2^* + \theta_5 a^* m_2^* + \theta_8' c) \\
&+ (\theta_2 + \theta_4 a^* + \theta_6 m_2^* + \theta_7 a^* m_2^*)(\gamma_0 + \gamma_1 a^* + \gamma_2' c) \\
= &\theta_1(a - a^*) + \theta_5(\beta_0 + \beta_1 a^* + \beta_4' c)(a - a^*) + \theta_4(\gamma_0 + \gamma_1 a^* + \gamma_2' c)(a - a^*) \\
&+ \theta_7(\beta_0 + \beta_1 a^* + \beta_4' c)(\gamma_0 + \gamma_1 a^* + \gamma_2' c)(a - a^*) + \theta_5(\beta_2 + \beta_3 a^*)(\gamma_0 + \gamma_1 a^* + \gamma_2' c)(a - a^*) \\
&+ \theta_7(\beta_2 + \beta_3 a^*)[\sigma_{M_1}^2 + (\gamma_0 + \gamma_1 a^* + \gamma_2' c)^2](a - a^*) - (\theta_1 + \theta_5 m_2^*)(a - a^*) \\
&- (\theta_4 + \theta_7 m_2^*)(\gamma_0 + \gamma_1 a^* + \gamma_2' c)(a - a^*) \\
= &\{\theta_1 + \theta_5(\beta_0 + \beta_1 a^* + \beta_4' c) + \theta_7(\beta_0 + \beta_1 a^* + \beta_4' c)(\gamma_0 + \gamma_1 a^* + \gamma_2' c) \\
&+ \theta_5(\beta_2 + \beta_3 a^*)(\gamma_0 + \gamma_1 a^* + \gamma_2' c) + \theta_7(\beta_2 + \beta_3 a^*)[\sigma_{M_1}^2 + (\gamma_0 + \gamma_1 a^* + \gamma_2' c)^2] \\
&- (\theta_1 + \theta_5 m_2^*) - \theta_7 m_2^*(\gamma_0 + \gamma_1 a^* + \gamma_2' c)\}(a - a^*).
\end{aligned}
$$

We next find the formula of $E[\mathrm{INT}_{\text{ref-}AM_1M_2}(m_1^*, m_2^*)|c]$ by subtracting $E[\mathrm{INT}_{\text{ref-}AM_2}(m_1^*, m_2^*)|c]$ from $E[\mathrm{INT}_{\text{ref-}AM_2+\mathrm{AM}_1M_2}(m_2^*)|c]$:

$$
\begin{aligned}
&E\Big[\mathrm{INT}_{\text{ref-}AM_1M_2}(m_1^*, m_2^*)|c\Big] \\
&= E\Big[\mathrm{INT}_{\text{ref-}AM_2+AM_1M_2}(m_2^*)|c\Big] - E\Big[\mathrm{INT}_{\text{ref-}AM_2}(m_1^*, m_2^*)|c\Big] \\
&= \{\theta_1 + \theta_7(\beta_0 + \beta_1 a^* + \beta_4' c)(\gamma_0 + \gamma_1 a^* + \gamma_2' c) + \theta_5(\beta_2 + \beta_3 a^*)(\gamma_0 + \gamma_1 a^* + \gamma_2' c) \\
&\quad + \theta_7(\beta_2 + \beta_3 a^*)[\sigma_{M_1}^2 + (\gamma_0 + \gamma_1 a^* + \gamma_2' c)^2] - (\theta_1 + \theta_5 m_2^*) - \theta_7 m_2^*(\gamma_0 + \gamma_1 a^* + \gamma_2' c) \\
&\quad - \theta_5(\beta_2 m_1^* + \beta_3 a^* m_1^* - m_2^*) - \theta_7 m_1^*(\beta_0 + \beta_1 a^* + \beta_2 m_1^* + \beta_3 a^* m_1^* + \beta_4' c - m_2^*)\}(a - a^*).
\end{aligned}
$$

S5.5 Natural MI effects

We derive the expected value of each interaction effect.

$$Y(a, M_1(a), M_2(a, M_1(a))) = \sum_{m_2}\sum_{m_1} Y(a, m_1, m_2) \times I(M_1(a) = m_1) \times I(M_2(a, m_1) = m_2)$$

$$\begin{aligned}
&\Rightarrow E[Y(a, M_1(a), M_2(a, M_1(a)))|c] \\
&= E\left[\sum_{m_2}\sum_{m_1} Y(a, m_1, m_2) \times I(M_1(a) = m_1) \times I(M_2(a, m_1) = m_2) \middle| c\right] \\
&= \sum_{m_2}\sum_{m_1} E[Y(a, m_1, m_2) \times I(M_1(a) = m_1) \times I(M_2(a, m_1) = m_2)|c] \\
&= \sum_{m_2}\sum_{m_1} E[Y(a, m_1, m_2)|c]E[I(M_1(a) = m_1)|c]E[I(M_2(a, m_1) = m_2)|c] \text{ by } A4\ A6 \\
&= \sum_{m_2}\sum_{m_1} E[Y(a, m_1, m_2)|c]\Pr(M_1(a) = m_1|c)\Pr(M_2(a, m_1) = m_2|c) \\
&= \sum_{m_2}\sum_{m_1} E[Y(a, m_1, m_2)|c]\Pr(M_1(a) = m_1|a, c)\Pr(M_2(a, m_1) = m_2|a, m_1, c) \text{ by } A3\ A5 \\
&= \sum_{m_2}\sum_{m_1} E[Y(a, m_1, m_2)|c]\Pr(M_1 = m_1|a, c)\Pr(M_2 = m_2|a, m_1, c) \text{ by consistency} \\
&= \sum_{m_2}\sum_{m_1} E[Y(a, m_1, m_2)|a, m_1, m_2, c]\Pr(M_1 = m_1|a, c)\Pr(M_2 = m_2|a, m_1, c) \text{ by } A1\ A2 \\
&= \sum_{m_2}\sum_{m_1} E[Y|a, m_1, m_2, c]\Pr(M_1 = m_1|a, c)\Pr(M_2 = m_2|a, m_1, c) \text{ by consistency} \\
&= \int_{m_2}\int_{m_1} E[Y|a, m_1, m_2, c]d\Pr(M_1 = m_1|a, c)d\Pr(M_2 = m_2|a, m_1, c) \\
&= \int_{m_2}\int_{m_1} (\theta_0 + \theta_1 a + \theta_2 m_1 + \theta_3 m_2 + \theta_4 a m_1 + \theta_5 a m_2 + \theta_6 m_1 m_2 + \theta_7 a m_1 m_2 \\
&\quad + \theta_8' c)d\Pr(M_1 = m_1|a, c)d\Pr(M_2 = m_2|a, m_1, c) \\
&= \int_{m_1}\int_{m_2} (\theta_0 + \theta_1 a + \theta_2 m_1 + \theta_3 m_2 + \theta_4 a m_1 + \theta_5 a m_2 + \theta_6 m_1 m_2 + \theta_7 a m_1 m_2 \\
&\quad + \theta_8' c)d\Pr(M_2 = m_2|a, m_1, c)d\Pr(M_1 = m_1|a, c) \\
&= \int_{m_1}\int_{m_2} [(\theta_0 + \theta_1 a + \theta_2 m_1 + \theta_4 a m_1 + \theta_8' c) + (\theta_3 + \theta_5 a + \theta_6 m_1 \\
&\quad + \theta_7 a m_1) m_2] d\Pr(M_2 = m_2|a, m_1, c)d\Pr(M_1 = m_1|a, c) \\
&= \int_{m_1} [(\theta_0 + \theta_1 a + \theta_2 m_1 + \theta_4 a m_1 + \theta_8' c) + (\theta_3 + \theta_5 a + \theta_6 m_1 + \theta_7 a m_1) \\
&\quad \times (\beta_0 + \beta_1 a + \beta_2 m_1 + \beta_3 a m_1 + \beta_4' c)]d\Pr(M_1 = m_1|a, c) \\
&= \int_{m_1} [(\theta_0 + \theta_1 a + \theta_8' c) + (\theta_2 + \theta_4 a) m_1 \\
&\quad + (\theta_3 + \theta_5 a + (\theta_6 + \theta_7 a) m_1)(\beta_0 + \beta_1 a + \beta_4' c + (\beta_2 + \beta_3 a) m_1)]d\Pr(M_1 = m_1|a, c) \\
&= (\theta_0 + \theta_1 a + \theta_8' c) + (\theta_3 + \theta_5 a)(\beta_0 + \beta_1 a + \beta_4' c) \\
&\quad + (\theta_2 + \theta_4 a)(\gamma_0 + \gamma_1 a + \gamma_2' c) + (\theta_6 + \theta_7 a)(\beta_0 + \beta_1 a + \beta_4' c)(\gamma_0 + \gamma_1 a + \gamma_2' c) \\
&\quad + (\theta_3 + \theta_5 a)(\beta_2 + \beta_3 a)(\gamma_0 + \gamma_1 a + \gamma_2' c) + (\theta_6 + \theta_7 a)(\beta_2 + \beta_3 a)[\sigma_{M_1}^2 + (\gamma_0 + \gamma_1 a + \gamma_2' c)^2]. \qquad (W1)
\end{aligned}$$

Similarly, we can obtain the following expected values for the rest of the counterfactual formulas.

$$E[Y(a, M_1(a), M_2(a^*, M_1(a)))|c] = (\theta_0 + \theta_1 a + \theta_8' c) + (\theta_3 + \theta_5 a)(\beta_0 + \beta_1 a^* + \beta_4' c) + (\theta_2 + \theta_4 a)(\gamma_0 + \gamma_1 a + \gamma_2' c) + (\theta_6 + \theta_7 a)(\beta_0 + \beta_1 a^* + \beta_4' c)(\gamma_0 + \gamma_1 a + \gamma_2' c) + (\theta_3 + \theta_5 a)(\beta_2 + \beta_3 a^*)(\gamma_0 + \gamma_1 a + \gamma_2' c) + (\theta_6 + \theta_7 a)(\beta_2 + \beta_3 a^*)[\sigma_{M_1}^2 + (\gamma_0 + \gamma_1 a + \gamma_2' c)^2], \quad (W2)$$

$$E[Y(a, M_1(a^*), M_2(a, M_1(a^*)))|c] = (\theta_0 + \theta_1 a + \theta_8' c) + (\theta_3 + \theta_5 a)(\beta_0 + \beta_1 a + \beta_4' c) + (\theta_2 + \theta_4 a)(\gamma_0 + \gamma_1 a^* + \gamma_2' c) + (\theta_6 + \theta_7 a)(\beta_0 + \beta_1 a + \beta_4' c)(\gamma_0 + \gamma_1 a^* + \gamma_2' c) + (\theta_3 + \theta_5 a)(\beta_2 + \beta_3 a)(\gamma_0 + \gamma_1 a^* + \gamma_2' c) + (\theta_6 + \theta_7 a)(\beta_2 + \beta_3 a)[\sigma_{M_1}^2 + (\gamma_0 + \gamma_1 a^* + \gamma_2' c)^2], \quad (W3)$$

$$E[Y(a^*, M_1(a), M_2(a, M_1(a)))|c] = (\theta_0 + \theta_1 a^* + \theta_8' c) + (\theta_3 + \theta_5 a^*)(\beta_0 + \beta_1 a + \beta_4' c) + (\theta_2 + \theta_4 a^*)(\gamma_0 + \gamma_1 a + \gamma_2' c) + (\theta_6 + \theta_7 a^*)(\beta_0 + \beta_1 a + \beta_4' c)(\gamma_0 + \gamma_1 a + \gamma_2' c) + (\theta_3 + \theta_5 a^*)(\beta_2 + \beta_3 a)(\gamma_0 + \gamma_1 a + \gamma_2' c) + (\theta_6 + \theta_7 a^*)(\beta_2 + \beta_3 a) \times [\sigma_{M_1}^2 + (\gamma_0 + \gamma_1 a + \gamma_2' c)^2], \quad (W4)$$

$$E[Y(a^*, M_1(a^*), M_2(a, M_1(a^*)))|c] = (\theta_0 + \theta_1 a^* + \theta_8' c) + (\theta_3 + \theta_5 a^*)(\beta_0 + \beta_1 a + \beta_4' c) + (\theta_2 + \theta_4 a^*)(\gamma_0 + \gamma_1 a^* + \gamma_2' c) + (\theta_6 + \theta_7 a^*)(\beta_0 + \beta_1 a + \beta_4' c)(\gamma_0 + \gamma_1 a^* + \gamma_2' c) + (\theta_3 + \theta_5 a^*)(\beta_2 + \beta_3 a)(\gamma_0 + \gamma_1 a^* + \gamma_2' c) + (\theta_6 + \theta_7 a^*)(\beta_2 + \beta_3 a) [\sigma_{M_1}^2 + (\gamma_0 + \gamma_1 a^* + \gamma_2' c)^2], \quad (W5)$$

$$E[Y(a^*, M_1(a), M_2(a^*, M_1(a)))|c] = (\theta_0 + \theta_1 a^* + \theta_8' c) + (\theta_3 + \theta_5 a^*)(\beta_0 + \beta_1 a^* + \beta_4' c) + (\theta_2 + \theta_4 a^*)(\gamma_0 + \gamma_1 a + \gamma_2' c) + (\theta_6 + \theta_7 a^*)(\beta_0 + \beta_1 a^* + \beta_4' c)(\gamma_0 + \gamma_1 a + \gamma_2' c) + (\theta_3 + \theta_5 a^*)(\beta_2 + \beta_3 a^*)(\gamma_0 + \gamma_1 a + \gamma_2' c) + (\theta_6 + \theta_7 a^*)(\beta_2 + \beta_3 a^*) [\sigma_{M_1}^2 + (\gamma_0 + \gamma_1 a + \gamma_2' c)^2], \quad (W6)$$

$$E[Y(a, M_1(a^*), M_2(a^*, M_1(a^*)))|c] = (\theta_0 + \theta_1 a + \theta_8' c) + (\theta_3 + \theta_5 a)(\beta_0 + \beta_1 a^* + \beta_4' c) + (\theta_2 + \theta_4 a)(\gamma_0 + \gamma_1 a^* + \gamma_2' c) + (\theta_6 + \theta_7 a)(\beta_0 + \beta_1 a^* + \beta_4' c)(\gamma_0 + \gamma_1 a^* + \gamma_2' c) + (\theta_3 + \theta_5 a)(\beta_2 + \beta_3 a^*)(\gamma_0 + \gamma_1 a^* + \gamma_2' c) + (\theta_6 + \theta_7 a)(\beta_2 + \beta_3 a^*) [\sigma_{M_1}^2 + (\gamma_0 + \gamma_1 a^* + \gamma_2' c)^2], \quad (W7)$$

$$E[Y(a^*, M_1(a^*), M_2(a^*, M_1(a^*)))|c] = (\theta_0 + \theta_1 a^* + \theta_8' c) + (\theta_3 + \theta_5 a^*)(\beta_0 + \beta_1 a^* + \beta_4' c) + (\theta_2 + \theta_4 a^*)(\gamma_0 + \gamma_1 a^* + \gamma_2' c) + (\theta_6 + \theta_7 a^*)(\beta_0 + \beta_1 a^* + \beta_4' c)(\gamma_0 + \gamma_1 a^* + \gamma_2' c) + (\theta_3 + \theta_5 a^*)(\beta_2 + \beta_3 a^*)(\gamma_0 + \gamma_1 a^* + \gamma_2' c) + (\theta_6 + \theta_7 a^*)(\beta_2 + \beta_3 a^*) [\sigma_{M_1}^2 + (\gamma_0 + \gamma_1 a^* + \gamma_2' c)^2]. \quad (W8)$$

The formulas of natural MI effects can be obtained as follows:

$$E\left[\text{NatINT}_{AM_1}|c\right] = (W2) - (W6) - (W7) + (W8) = [\theta_4\gamma_1 + \theta_7\gamma_1(\beta_0 + \beta_1 a^* + \beta_4' c) + \theta_5\gamma_1(\beta_2 + \beta_3 a^*) + 2\theta_7\gamma_1(\beta_2 + \beta_3 a^*)(\gamma_0 + \gamma_2' c) + \theta_7\gamma_1^2(\beta_2 + \beta_3 a^*)(a + a^*)](a - a^*)^2,$$

$$E\left[\text{NatINT}_{AM_2}|c\right] = (W3) - (W5) - (W7) + (W8) = [\theta_5\beta_1 + \theta_7\beta_1(\gamma_0 + \gamma_1 a^* + \gamma_2' c) + \theta_5\beta_3(\gamma_0 + \gamma_1 a^* + \gamma_2' c) + \theta_7\beta_3[\sigma_{M_1}^2 + (\gamma_0 + \gamma_1 a^* + \gamma_2' c)^2]](a - a^*)^2,$$

$$E\left[\text{NatINT}_{AM_1M_2}|c\right] = (W1) - (W4) - (W3) + (W5) - (W2) + (W6) + (W7) - (W8) = [\theta_7\beta_1\gamma_1 + \theta_5\beta_3\gamma_1 + 2\theta_7\beta_3\gamma_1(\gamma_0 + \gamma_2' c) + \theta_7\beta_3\gamma_1^2(a + a^*)](a - a^*)^3,$$

$$E\left[\text{NatINT}_{M_1M_2}|c\right] = (W4) - (W5) - (W6) + (W8)$$
$$= [\beta_1\gamma_1(\theta_6 + \theta_7a^*) + \beta_3\gamma_1(\theta_3 + \theta_5a^*)$$
$$+ 2\beta_3\gamma_1(\theta_6 + \theta_7a^*)(\gamma_0 + \gamma_2'c) + \beta_3\gamma_1^2(\theta_6 + \theta_7a^*)(a + a^*)](a - a^*)^2.$$

S5.6 PIE through M_1

The PIE through M_1 can be obtained by the following derivation:

$$\text{PIE}_{M_1} = Y(a^*, M_1(a), M_2(a^*, M_1(a))) - Y(a^*, M_1(a^*), M_2(a^*, M_1(a^*)))$$
$$\Rightarrow E\left[\text{PIE}_{M_1}|c\right] = E[Y(a^*, M_1(a), M_2(a^*, M_1(a))) - Y(a^*, M_1(a^*), M_2(a^*, M_1(a^*)))|c]$$
$$= E[Y(a^*, M_1(a), M_2(a^*, M_1(a)))|c] - E[Y(a^*, M_1(a^*), M_2(a^*, M_1(a^*)))|c]$$
$$= (W6) - (W8)$$
$$= [\gamma_1(\theta_2 + \theta_4a^*) + \gamma_1(\theta_6 + \theta_7a^*)(\beta_0 + \beta_1a^* + \beta_4'c) + \gamma_1(\theta_3 + \theta_5a^*)(\beta_2 + \beta_3a^*)$$
$$+ 2\gamma_1(\theta_6 + \theta_7a^*)(\beta_2 + \beta_3a^*)(\gamma_0 + \gamma_2'c)+\gamma_1^2(\theta_6 + \theta_7a^*)(\beta_2 + \beta_3a^*)(a + a^*)](a - a^*).$$

S5.7 Seminatural indirect effect through M_2

The seminatural indirect effect through M_2 can be obtained by the following derivation:

$$\text{SNIE}_{M_2} = Y(a^*, M_1(a^*), M_2(a, M_1(a^*))) - Y(a^*, M_1(a^*), M_2(a^*, M_1(a^*)))$$
$$\Rightarrow E\left[\text{SNIE}_{M_2}|c\right] = E[Y(a^*, M_1(a^*), M_2(a, M_1(a^*))) - Y(a^*, M_1(a^*), M_2(a^*, M_1(a^*)))|c]$$
$$= E[Y(a^*, M_1(a^*), M_2(a, M_1(a^*)))|c] - E[Y(a^*, M_1(a^*), M_2(a^*, M_1(a^*)))|c]$$
$$= (W5) - (W8)$$
$$= [\beta_1(\theta_3 + \theta_5a^*) + \beta_1(\theta_6 + \theta_7a^*)(\gamma_0 + \gamma_1a^* + \gamma_2'c) + \beta_3(\theta_3 + \theta_5a^*)(\gamma_0 + \gamma_1a^* + \gamma_2'c)$$
$$+ \beta_3(\theta_6 + \theta_7a^*)[\sigma_{M_1}^2 + (\gamma_0 + \gamma_1a^* + \gamma_2'c)^2]](a - a^*).$$

S5.8 TE

$$\text{TE} = Y(a, M_1(a), M_2(a, M_1(a))) - Y(a^*, M_1(a^*), M_2(a^*, M_1(a^*)))$$
$$\Rightarrow E[TE|c] = E[Y(a, M_1(a), M_2(a, M_1(a))) - Y(a^*, M_1(a^*), M_2(a^*, M_1(a^*)))|c]$$
$$= E[Y(a, M_1(a), M_2(a, M_1(a)))|c] - E[Y(a^*, M_1(a^*), M_2(a^*, M_1(a^*)))|c]$$
$$= (W1) - (W8)$$
$$= [\theta_1 + \theta_5(\beta_0 + \beta_4'c) + \beta_1\theta_3 + \theta_4(\gamma_0 + \gamma_2'c) + \gamma_1\theta_2$$
$$+ \theta_7(\beta_0 + \beta_4'c)(\gamma_0 + \gamma_2'c) + \beta_1\theta_6(\gamma_0 + \gamma_2'c) + \gamma_1\theta_6(\beta_0 + \beta_4'c) + \theta_5\beta_2(\gamma_0 + \gamma_2') + \theta_3\beta_3(\gamma_0 + \gamma_2'c)$$
$$+ \theta_3\beta_2\gamma_1 + \theta_7\beta_2\sigma_{M_1}^2 + \theta_6\beta_3\sigma_{M_1}^2 + \theta_7\beta_2(\gamma_0 + \gamma_2'c)^2 + \theta_6\beta_3(\gamma_0 + \gamma_2'c)^2 + 2\gamma_1\theta_6\beta_2(\gamma_0 + \gamma_2'c)](a - a^*)$$
$$+ [\beta_1\theta_5 + \gamma_1\theta_4 + \beta_1\theta_7(\gamma_0 + \gamma_2'c) + \gamma_1\theta_7(\beta_0 + \beta_4'c) + \gamma_1\beta_1\theta_6 + \theta_5\beta_3(\gamma_0 + \gamma_2'c)$$
$$+ \theta_5\beta_2\gamma_1 + \theta_3\beta_3\gamma_1 + \theta_7\beta_3\sigma_{M_1}^2 + \theta_7\beta_3(\gamma_0 + \gamma_2'c)^2$$
$$+ 2\gamma_1\theta_7\beta_2(\gamma_0 + \gamma_2'c) + 2\gamma_1\theta_6\beta_3(\gamma_0 + \gamma_2'c) + \theta_6\beta_2\gamma_1^2](a^2 - a^{*2})$$
$$+ [\gamma_1\beta_1\theta_7 + \theta_5\beta_3\gamma_1 + 2\gamma_1\theta_7\beta_3(\gamma_0 + \gamma_2'c) + \theta_7\beta_2\gamma_1^2 + \theta_6\beta_3\gamma_1^2](a^3 - a^{*3}) + \theta_7\beta_3\gamma_1^2(a^4 - a^{*4}).$$

References

[1] VanderWeele TJ, Vansteelandt S. Mediation analysis with multiple mediators. Epidemiol Methods. 2014;2(1):95–115.
[2] VanderWeele TJ, Vansteelandt S, Robins JM. Effect decomposition in the presence of an exposure-induced mediator-outcome confounder. Epidemiology. 2014;25(2):300–6.
[3] Daniel RM, De Stavola BL, Cousens SN, Vansteelandt S. Causal mediation analysis with multiple mediators. Biometrics. 2015;71(1):1–14.
[4] Steen J, Loeys T, Moerkerke B, Vansteelandt S. Flexible mediation analysis with multiple mediators. Am J Epidemiol. 2017;186(2):184–93.
[5] Mittinty MN, Lynch JW, Forbes AB, Gurrin LC. Effect decomposition through multiple causally nonordered mediators in the presence of exposure-induced mediator-outcome confounding. Stat Med. 2019;38(26):5085–102.
[6] VanderWeele TJ. A three-way decomposition of a total effect into direct, indirect, and interactive effects. Epidemiology. 2013;24(2):224–32.
[7] VanderWeele TJ. A unification of mediation and interaction: a 4-way decomposition. Epidemiology. 2014;25(5):749–61.
[8] VanderWeele TJ. Explanation in Causal Inference: Methods for Mediation and Interaction. New York: Oxford University Press; 2015.
[9] Bellavia A, Valeri L. Decomposition of the total effect in the presence of multiple mediators and interactions. Am J Epidemiol. 2018;187(6):1311–8.
[10] Taguri M, Featherstone J, Cheng J. Causal mediation analysis with multiple causally non-ordered mediators. Stat Methods Med Res. 2018;27(1):3–19.
[11] Rothman KJ, Greenland S, Lash TL. Concepts of interaction. In: Modern epidemiology. Chapter 5, 3rd ed. Philadelphia, PA: Lippincott Williams and Wilkins. p. 2008:71–84.
[12] Hosmer DW, Lemeshow S. Confidence interval estimation of interaction. Epidemiology. 1992;3(5):452–56.
[13] VanderWeele TJ, Tchetgen Tchetgen EJ. Mediation analysis with time varying exposures and mediators. J R Statist Soc B. 2017;79(3):917–38.
[14] Daniel RM, De Stavola BL, Cousens SN. Gformula: Estimating causal effects in the presence of time-varying confounding or mediation using the g-computation formula. Stata J. 2011;11(4):479–517.
[15] Avin C, Shpitser I, Pearl J. Identifiability of path-specific effects. In: Proceedings of the International Joint Conferences on Artificial Intelligence. Edinburgh, Schotland; 2005. p. 357–63.
[16] Robins JM, Greenland S. Identifiability and exchangeability for direct and indirect effects. Epidemiology. 1992;3(2):143–55.
[17] Pearl J. Direct and indirect effects. In: Proceedings of the Seventeenth Conference on Uncertainty in Artificial Intelligence. San Francisco, CA: Morgan Kaufmann Publishers Inc; 2001. p. 411–20.
[18] Robins JM. Semantics of causal DAG models and the identification of direct and indirect effects. In: Green JP, Hjort NL, Richardson S, eds. Highly structured stochastic systems. New York: Oxford University Press; 2003. p. 70–81.
[19] Pearl J. Interpretation and identification of causal mediation. Psychol Methods. 2014;19(4):459–81.
[20] Huber M. Identifying causal mechanisms in experiments (primarily) based on inverse probability weighting (Technical Report). St. Gallen, Switzerland: University of St. Gallen, Department of Economics; 2012.
[21] VanderWeele TJ. Policy-relevant proportions for direct effects. Epidemiology. 2013;24(1):175–6.
[22] VanderWeele TJ, Vansteelandt S. Conceptual issues concerning mediation, interventions and composition. Stat Its Interface. 2009;2(4):457–68.
[23] Robins JM, Richardson TS. Alternative graphical causal models and the identification of direct effects. In: Shrout P, eds. Causality and psychopathology: finding the determinants of disorders and their cures. New York: Oxford University Press; 2010.
[24] Valeri L, VanderWeele TJ. Mediation analysis allowing for exposure-mediator interactions and causal interpretation: Theoretical assumptions and implementation with SAS and SPSS macros. Psychol Methods. 2013;18(2):137–50.
[25] Conigrave KM, Davies P, Haber P, Whitfield JB. Traditional markers of excessive alcohol use. Addiction. 2003;98(Suppl 2):31–43.
[26] Lim JS, Yang JH, Chun BY, Kam S, Jacobs, Jr. DR, Lee DH. Is serum gamma-glutamyltransferase inversely associated with serum antioxidants as a marker of oxidative stress? Free Radic Biol Med. 2004;37(7):1018–23.
[27] Lee DH, Blomhoff R, Jacobs, Jr. DR Is serum gamma glutamyltransferase a marker of oxidative stress? Free Radic Res. 2004;38(6):535–9.
[28] Colicchio P, Tarantino G, delGenio F, Sorrentino P, Saldalamacchia G, Finelli C, et al. Non-alcoholic fatty liver disease in young adult severely obese non-diabetic patients in South Italy. Ann Nutr Metab. 2005;49(5):289–95.
[29] Daeppen JB, Smith TL, Schuckit MA. Influence of age and body mass index on gamma-glutamyltransferase activity: A 15-year follow-up evaluation in a community sample. Alcohol Clin Exp Res. 1998;22(4):941–4.
[30] Zhang H, Forman HJ. Redox regulation of gamma-glutamyl transpeptidase. Am J Respir Cell Mol Biol. 2009;41(5):509–15.
[31] Fentiman IS. Gamma-glutamyl transferase: risk and prognosis of cancer. Br J Cancer. 2012;106(9):1467–8.
[32] Jiang S, Jiang D, Tao Y. Role of gamma-glutamyltransferase in cardiovascular diseases. Exp Clin Cardiol. 2013;18(1):53–6.

[33] Baros AM, Wright TM, Latham PK, Miller PM, Anton RF. Alcohol consumption, % CDT, GGT and blood pressure change during alcohol treatment. Alcohol Alcohol. 2008;43(2):192–7.
[34] Song SH, Kwak IS, Kim YJ, Kim SJ, Lee SB, Lee DW. Can gamma-glutamyltransferase be an additional marker of arterial stiffness? Circ J. 2007;71(11):1715–20.
[35] Saijo Y, Utsugi M, Yoshioka E, Horikawa N, Sato T, Gong Y. The relationship of gamma-glutamyltransferase to C-reactive protein and arterial stiffness. Nutr Metab Cardiovasc Dis. 2008;18(3):211–9.
[36] Tsai J, Ford ES, Zhao G, Li C, Greenlund KJ, Croft JB. Co-occurrence of obesity and patterns of alcohol use associated with elevated serum hepatic enzymes in US adults. J Behav Med. 2012;35(2):200–10.
[37] Puukka K, Hietala J, Koivisto H, Anttila P, Bloigu R, Niemelä O. Additive effects of moderate drinking and obesity on serum gamma-glutamyl transferase activity. Am J Clin Nutr. 2006;83(6):1351–4.
[38] Stranges S, Trevisan M, Dorn JM, Dmochowski J, Donahue RP. Body fat distribution, liver enzymes, and risk of hypertension: evidence from the western New York study. Hypertension. 2005;46(5):1186–93.
[39] Leon DA, Saburova L, Tomkins S, Andreev E, Kiryanov N, McKee M, et al. Hazardous alcohol drinking and premature mortality in Russia: A population based case-control study. Lancet. 2007;369(9578):2001–9.
[40] U.S. Department of Agriculture and U.S. Department of Health and Human Services. 2020-2025 Dietary Guidelines for Americans. 9th Edition, Washington, DC; 2020.
